# Enhanced 4Pi single-molecule localization microscopy with coherent pupil based localization

**DOI:** 10.1038/s42003-020-0908-2

**Published:** 2020-05-08

**Authors:** Sheng Liu, Fang Huang

**Affiliations:** 1 Weldon School of Biomedical Engineering, Purdue University, West Lafayette, IN USA; 2 Purdue Institute for Integrative Neuroscience, Purdue University, West Lafayette, IN USA; 3 Purdue Institute of Inflammation, Immunology and Infectious Disease, Purdue University, West Lafayette, IN USA

**Keywords:** Optical imaging, Super-resolution microscopy

## Abstract

Over the last decades, super-resolution techniques have revolutionized the field of fluorescence microscopy. Among them, interferometric or 4Pi microscopy methods exhibit supreme resolving power in the axial dimension. Combined with single-molecule detection/localization and adaptive optics, current 4Pi microscopy methods enabled 10–15 nm isotropic 3D resolution throughout whole cells. However, further improving the achieved 3D resolution poses challenges arising from the complexity of single-molecule emission patterns generated by these coherent single-molecule imaging systems. These complex emission patterns render a large portion of information carrying photons unusable. Here, we introduce a localization algorithm that achieves the theoretical precision limit for a 4Pi based single-molecule switching nanoscopy (4Pi-SMSN) system, and demonstrate improvements in localization precision, accuracy as well as stability comparing with state-of-the-art 4Pi-SMSN methods.

## Abstract (web summary)

To further improve localisation precision and biases in all the three dimensions of 4Pi single molecule localisation microscopy, Huang and Liu develop a method based on coherent pupil functions and an algorithm to extract the position information content at the theoretical information limit while dynamically compensating the temperature-induced cavity drift.

## Introduction

The achievable resolution of a far-field fluorescence microscope was constrained by the diffraction limit of light, approximately 200–300 nm in the lateral direction and 500–700 nm in the axial direction. Over the last decades, substantial efforts were made to overcome this resolution limit. Based on confocal and widefield microscopy geometries respectively, 4Pi (type A-C)^1–3^ and I^n^M^4–6^ methods use coherent illumination and/or detection based on two opposing objectives to improve the resolution by 3–7 fold in the axial dimension^7^. Combining coherent detection and the stochastic switching of single molecules with high-precision localization, 4Pi (or interferometric) based single-molecule switching nanoscopy (SMSN) techniques, such as iPALM^8,9^ and 4Pi-SMSN^10^, allow another 5–10 fold improvement in the axial resolution^8,11^ in comparison with conventional 3D SMSN. Further incorporating adaptive optics and interferometry-specific algorithm design, W-4PiSMSN^12^ allowed high-resolution reconstruction of the whole mammalian cell (up to ~9 μm) without deterioration of resolution throughout the imaging depth.

The resolution enhancing capacity of the interferometric systems comes from their complex point spread function (PSF) patterns, thereafter, referred as 4PiPSF. One of its distinct features is the rapid intensity modulation within the pattern center along the depth (axial) direction which, in turn, improves the axial resolution when combined with confocal, stimulated emission depletion microscopy and single-molecule localization microscopy methods. Aside from the center peak, the complexities of 4PiPSF majorly arise from the features such as rings and lobes at its periphery. Depending on the axial position of the single emitter, these features contains up to 80% of the entire 4PiPSF energy (Methods). Theoretical precision calculations based on the Fisher information theory suggested that better localization precision can be obtained in both axial and lateral directions^11,13^ when compared with other incoherent dual-objective systems^14^. To date, these complex features remain largely unexplored when localizing single-molecule emission patterns from 4Pi systems, due to the challenge in modeling this complex pattern with high accuracy. An accurate 4PiPSF model must be able to take into account of the static imperfections of the interferometric single-molecule imaging system, such as aberrations and transmission variances in both interferometric arms, partial coherence due to the broadband emission spectra and the dynamic changes of the system, such as the temperature-dependent changes of the interferometric cavity length.

To overcome those difficulties, we introduce a method based on coherent pupil functions to allow accurate modeling of 4PiPSFs and develop an localization algorithm to extract the position information content at the theoretical information limit while, at the same time, dynamically compensates the temperature-induced cavity drift. The confluence of these new analytical methods improves both localization precision and bias in all three dimensions compared with existing 4Pi-SMSN systems^10,12^ and at the same time extends imaging volume in the axial direction.

## Results

### 4PiPSF modeling with coherent phase-retrieved pupils

The hallmark of a 4PiPSF generated by an interferometric microscopy system is the distinct multi-lobe PSF in both axial and lateral dimensions. Currently, extracting single-molecule axial positions from the center lobes (including center peak intensity and intensities falls within a empirically defined ring region) has been the major focus in 4Pi single-molecule localization analysis^8,10,12^, discarding or underutilizing substantial amount of information-carrying photons among the lateral side lobes. At the same time, when pin-pointing molecular centers at the lateral dimension (i.e., localization in *x*, *y*) the interferometric features of the 4PiPSFs have been, to a large extent, ignored^8,12^. As a result, no lateral resolution enhancement is reported comparing with conventional PSFs^8,12^.

To allow extracting information contained within the complex interferometric PSF, we describe here a PSF model based on two coherent pupil functions for 4Pi single-molecule imaging systems^10,12^. This method allowed us to create a realistic model of the interferometric emission patterns taking into account of the independent wave front distortions from the two interference arms, the wavelength-dependent coherence modulation and the spontaneous interferometric path length drift.

**Fig. 1:**
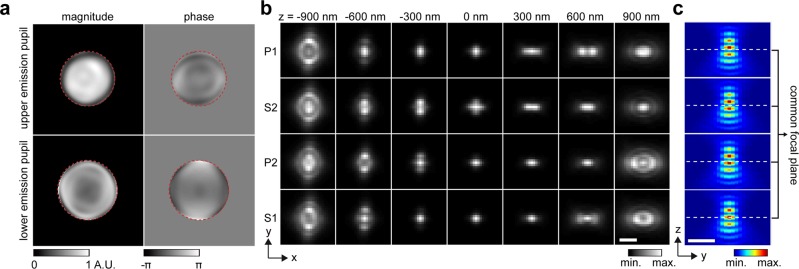
Coherent, phase-retrieved pupil based 4PiPSF model. **a** Pupil functions of upper and lower emission paths, independently retrieved by imaging a fluorescence bead on the bottom cover glass. Numerical apertures (NA) of 1.3 and 1.4 (the objective NA), defining the cutoff frequency in the Fourier space (red dash circles), were used during the phase retrieval of the upper and lower emission pupils. The slight shrinking of the upper emission-pupil size is caused by index mismatch aberration (Supplementary Note 6). Astigmatism aberrations (the 5th Zernike polynomial, Wyant order)^33^ with amplitudes of 2 and −1.5 (unit: λ/2π) were applied to the upper and lower deformable mirrors respectively (Methods). **b** PR-4PiPSF models at various axial positions. Each axial position including four PSF patterns with different polarizations and phases (channels P1, S2, P2, and S1). **c** PR-4PiPSF models in the *y*–*z* plane corresponding to the four channels in **b**. Scale bars: 1 µm in **b**, **c**.

Let $h_{\mathrm{A}}$ and $h_{\mathrm{B}}$ be a pair of pupil functions of the 4Pi system, representing the wave fields at the pupil planes of the upper and lower interference paths respectively (Fig. 1). The two pupil functions, retrieved independently through a phase retrieval (PR) algorithm^15–17^, allowed us to incorporate aberrations for both interference paths. Interferometric PSFs (termed as 4PiPSF) are generated from the superposition of the wave fields, $h_{\mathrm{A}}$ and $h_{\mathrm{B}}$. Four superposed wave fields with different polarizations and phase shifts can be written as,

$$h_{{\mathrm{S}}1} = I_th_AD\left( { - z} \right)e^{i\pi } + h_{\mathrm{B}}D\left( z \right)e^{i(\varphi _{{\mathrm{sp}}} + \varphi _0)},$$

$$h_{{\mathrm{S}}2} = I_{\mathrm{t}}h_{\mathrm{A}}D\left( { - z} \right) + h_{\mathrm{B}}D\left( z \right)e^{i(\varphi _{{\mathrm{sp}}} + \varphi _0)},$$

$$h_{{\mathrm{P}}1} = I_{\mathrm{t}}h_{\mathrm{A}}D\left( { - z} \right) + h_{\mathrm{B}}D\left( z \right)e^{i(\pi + \varphi _0)},$$

1 $$h_{{\mathrm{P}}2} = I_{\mathrm{t}}h_{\mathrm{A}}D\left( { - z} \right) + h_{\mathrm{B}}D\left( z \right)e^{i\varphi _0}.$$

Here, we assume $h_{\mathrm{A}}$ and $h_{\mathrm{B}}$ are independent against polarization directions, where $\varphi _{{\mathrm{sp}}} = 2\pi \delta L(n_{\mathrm{s}} - n_{\mathrm{p}})/\lambda$, with $n_{\mathrm{s}}$ and $n_{\mathrm{p}}$ the refractive indices of s- and p-polarized light in quartz, and $\delta L$ the quartz thickness difference between the two interference paths (Supplementary Note 1). $\varphi _0$ is the phase difference between the two interference arms when the single emitter is in the common focus of the two objectives and thereafter we refer $\varphi _0$ as the cavity phase. The defocus term $D\left( z \right)$ equals to ${\mathrm{exp}}(ik_zz)$, with $k_z$ being the $z$ (axial) component of the wave vector and $z$ is the relative axial position of the single emitter (axial distance to the common focus of the two objective lenses). A factor $I_{\mathrm{t}}$, referred as the intensity ratio, was introduced to account for the transmission efficiency difference between the two interference paths. The value of $I_{\mathrm{t}}$ was estimated for each experiment (Supplementary Notes 1–2, Supplementary Figs. 1–2).

We can then write out the coherent PSF, $\mu _{{\mathrm{I}}m}$, in terms of Fourier transform of each superposed wave field,

2 $$\mu _{{\mathrm{I}}m}\left( {x,y,z} \right) = \left| {{\cal{F}}[h_m(k_x,k_y)]} \right|^2,m \in \left( {{\mathrm{S}}1,{\mathrm{S}}2,{\mathrm{P}}1,{\mathrm{P}}2} \right),$$

where subscript I indicates interference and Eq. 2 assumes that the wave fields $h_{\mathrm{A}}$ and $h_{\mathrm{B}}$ are perfectly coherent. However, because of the finite spectral width of the emission filter, the estimated coherence length of the emission light is small, ~7.5 µm (with an emission filter of 700/50 nm, center wavelength/band width). Therefore, a slight change of the optical path length difference (OPD) between the two interference arms will result in a moderate reduction of the modulation depth—the peak to valley contrast in an interferometric PSF and the degree of this reduction is wavelength dependent. To this end, we assume $h_{\mathrm{A}}$ and $h_{\mathrm{B}}$ are partially coherent and the incoherent part will produce a conventional incoherent PSF described as $\mu _{\mathrm{W}}$,

3 $$\mu _{\mathrm{W}}\left( {x,y,z} \right) = \left| {{\cal{F}}[I_{\mathrm{t}}h_{\mathrm{A}}(k_x,k_y)D\left( { - z} \right)]} \right|^2 + \left| {{\cal{F}}[h_{\mathrm{B}}(k_x,k_y)D\left( z \right)]} \right|^2,$$

with subscript W indicates wide-field in the sense of conventional microscope. Therefore, the final PSF is a combination of the interferometric PSF and the conventional PSF with an empirical factor $a \in [0,1]$, describing the coherence strength (Supplementary Note 2 and Supplementary Fig. 2),

4 $$\mu _{m0}\left( {x,y,z} \right) = a\mu _{{\mathrm{I}}m}\left( {x,y,z} \right) + \left( {1 - a} \right)\mu _{\mathrm{W}}\left( {x,y,z} \right),$$

where $\mu _0$ represents the normalized PSF (sum of the PSF intensities in s- or p-polarization equals to 1). By adding a total photon count of $I$ and a background of $b$, the PR-4PiPSF model is,

5 $$\mu _m\left( {x,y,z} \right) = I_m\mu _{m0}\left( {x,y,z} \right) + b_m,$$

which includes quadruple PSFs for each emitter (Fig. 1). Here all $I_m$ and $b_m$ were considered as independent to account for the difference in transmission efficiency between the two polarizations and the emission paths after the beam splitter^12^. We found that PR-4PiPSFs can produce relatively uniform resolutions within a large $a$ range (0.5–1), an attractive feature for thicker specimens, where $a$ is depth dependent (Supplementary Fig. 2 and Supplementary Note 2).

**Fig. 2:**
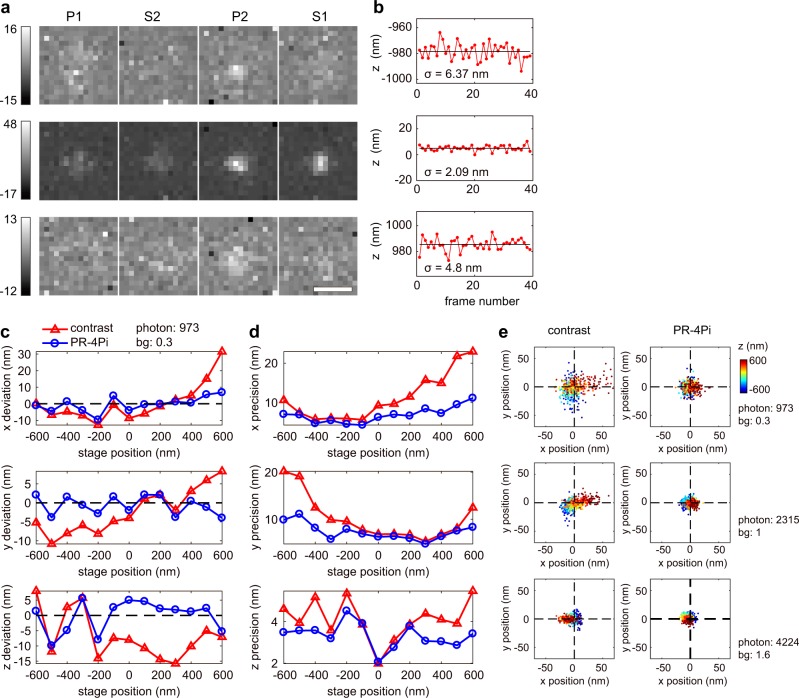
Comparison of localization results of bead data using PR-4Pi and contrast methods. **a** Examples of experimental bead images with relatively low detected photons at various axial positions in **b**, each contains PSF patterns from four channels (P1, S2, P2, and S1). The pixel values represent the number of photon–electrons corrupted with sCMOS readout noise^20^. **b** Axial localization results of the corresponding bead data in **a**. Notice that in spite of the low photon budget, PR-4Pi localization method achieves a precision ~5 nm. The estimation results fluctuated around the mean (solid black line) with a standard deviation of $\sigma$. **c** Localization deviations in $x$, $y$, and $z$, mean (*n* = 39). **d** Localization precisions in $x$, $y$, and $z$, s.d. (*n* = 39). **e** Scatter plots of lateral localization results from different bead data. The PR-4Pi method shows obvious improvement in both localization accuracy and precision. Contrast method in this work refers to the algorithm developed in ref. ^12^ that extracts the axial positions using the Gaussian-weighted 0th central moment (M0, Supplementary Note 4 and Supplementary Table 1). Scale bar: 1 µm in **a**. Sample: 40 nm dark red bead, photon: median total photon per objective (*n* = 819), bg: median background photon per pixel (*n* = 819). Photon and background were estimated from PR-4Pi method.

As a demonstration of the accuracy of the PR-4PiPSF model, we tested it with the experimental PSFs obtained by imaging 40 nm beads attached on the coverslip surface. We pin-pointed 3D positions of isolated beads with various photon counts and compared with the position readout from a high-precision piezo stage (±0.5 nm close-loop) (Fig. 2, Supplementary Figs. 3–5). We found that comparing with ideal-4PiPSF model (where $h_{\mathrm{A}}$ and $h_{\mathrm{B}}$ are unaberrated pupil functions, with $I_{\mathrm{t}}$ and $a$ equal to 1, Supplementary Fig. 3), PR-4PiPSF model based localization results in comparable localization accuracy (bias) in the lateral dimension and improved localization accuracy in the axial dimension (Supplementary Fig. 4). When comparing with contrast-based method (referring to the method using incoherent PSF models for lateral localization and PSF’s central moment for axial localization^12^), both ideal- and PR-4PiPSF models show improved localization accuracies in both lateral and axial directions (Supplementary Fig. 4). We also notice that the localization deviations (bias) caused by contrast-based method are bead specific (Fig. 2, Supplementary Figs. 4–5, all bead data were collected from the same sample), especially in the lateral dimension, making it difficult to perform post acquisition correction due to the random nature of such deviations.

### Information content within a 4PiPSF

Given the ability to generate a realistic 4PiPSF model, we are able to quantitatively investigate the information content within the 4PiPSF pattern recorded on our microscope using Fisher information matrix^18,19^. By quantifying information content pixel by pixel within the 4PiPSF (as similarly shown previously^11^ for ideal PSFs), we found previous 4Pi-SMSN localization analysis either discarded the additional information from interferometric detection (as for lateral localization, Supplementary Note 3 includes derivation of a general case for such information gain) or incorporated only part of it (as for the axial localization that uses 0th and/or 3rd central moments of the PSFs, Supplementary Figs. 6–7). Incorporating a comprehensive and accurate PSF model allows us to further improve localization accuracy and precision in three dimensions.

To quantify the information content of 4PiPSFs, we calculated the Fisher information matrix, which quantifies the lower bound of the localization precision for an unbiased estimator: the Cramér–Rao lower bound (CRLB)^19^,

6 $${\mathrm{var}}\left( {\theta _i} \right) \ge [F\left( \theta \right)^{ - 1}]_{ii},$$

where *F* is the Fisher information matrix, $\theta$ is a vector of estimation parameters, *i* denotes the index of each parameter.

Incorporating Poisson noise and the readout noise from an sCMOS camera, a numerical calculation of each element in Fisher information matrix is^20^,

7 $$F_{ij} = \mathop {\sum }\limits_m \mathop {\sum }\limits_q \frac{1}{{\mu _{mq} + \gamma _{mq}}}\frac{{\Delta \mu _{mq}}}{{\Delta \theta _{mi}}}\frac{{\Delta \mu _{mq}}}{{\Delta \theta _{mj}}},(\theta _i,\theta _j) \in (x,y,z,I_m,b_m),$$

where *q* is the pixel index and *m* is the index of the quadruple 4PiPSFs, the factor$\gamma$ equals to $\sigma ^2/g^2$, where $\sigma ^2$ is the variance of the readout noise per pixel and *g* is the gain of each pixel.

In comparison with incoherent dual-objective PSFs^14^, we observed the predicted information in a 4PiPSF increase significantly along the axial dimension (17–160 folds at 1000 total emitted photon and 10 background photons) and a relatively large information gain at the out-of-focus regions along the lateral dimension (5–10 folds at 1000 total emitted photon and 10 background photon) (Supplementary Figs. 8–9). Comparing to an ideal-4PiPSF (Supplementary Fig. 3), the information contained in a realistic PSF model as PR-4PiPSF, is slightly lower (Supplementary Fig. 9).

This observed information-content boost predicts and quantifies the improvement in the achievable localization precisions in all three dimensions by using 4PiPSFs. Existing 4Pi localization methods obtain the lateral position estimation by either merging the multichannel 4PiPSFs into incoherent dual-objective PSFs^8,9,12^ or modifying the PSFs with a Gaussian mask^10^ and applying a Gaussian or Airy disk PSF model on the merged or modified PSFs (Supplementary Table 1). These methods result in reduction of information since the interference pattern is not fully considered leading to up to twofold worse localization precisions when compared with the information limit, of which the deterioration is increasingly pronounced when emitters are located away from the common focus of the two objective lenses (Supplementary Figs. 6–7). As for axial localization, previously, position estimations were extracted from intensity contrast calculated from PSFs at different detection channels and such calculation often involves summing over all or partial pixel values (e.g., using the central moments, Supplementary Table 1) within the PSF of each channel. We found that these methods perform well within a small distance (±300 nm) from the common focus region but result in partial information loss, where the achieved precision (uncertainty value) and bias of these methods increase rapidly with the axial position of a single emitter (Supplementary Figs. 6–7).

In order to utilize the complete information contained within the interferometric PSF pattern and minimize localization biases, we combined our coherent pupil based 4PiPSFs model with a maximum likelihood estimator with the appropriate noise model^20^ (sCMOS noise model in our case) for three dimensional localization of 4PiPSF patterns (termed as PR-4Pi algorithm, Supplementary Notes 4–5, Supplementary Table 2). We demonstrated in the following sections that the PR-4Pi algorithm achieve the theoretical precision limit (defined by CRLB) of our 4Pi-SMSN system in both axial and lateral dimensions.

As a demonstration of PR-4Pi localization algorithm, first we tested the algorithm through a set of simulated 4PiPSFs. While the localization precisions using conventional intensity-contrast based methods varies drastically in different axial position, we found PR-4Pi algorithm provides localization precisions consistently approaching the theoretical information limit calculated by CRLB. A detailed characterization of PR-4Pi algorithm and other existing 4Pi localization algorithms are shown in Supplementary Fig. 6 and Supplementary Table 1. For fair comparison with existing 4Pi algorithms based on Gaussian mask for lateral and central moments for axial localizations^10^, we generate 4PiPSFs without astigmatism modification (Supplementary Figs. 6, 8, 10, astigmatism modification^9,12^ were used to reduce localization artifacts). We found that for lateral localization, Gaussian mask method performs worse than using incoherent PSFs (contrast-based method, Supplementary Fig. 6). And for axial localization, combining 0th and 3rd central moments^10^ achieves better precision at out-of-focus region than using only the 0th moment (contrast-based method), but still fail to achieve the theoretical limit (CRLB) when considering equal split of photon energy through the four detected channels (five estimation parameters include: *x*, *y*, *z*, *I,* and *b*, Supplementary Fig. 6 and Supplementary Note 4). Interestingly, when considering independent intensity and background for each channel (11 estimation parameters), the CRLB for axial estimation increases, which results from an anticorrelation between estimations on intensity/background and axial positions with astigmatism modification (Supplementary Note 4 and Supplementary Fig. 7). Nonetheless, in both cases, PR-4Pi algorithm achieves CRLB within the entire tested range (see Supplementary Note 6, Supplementary Figs. 11–15 for robustness test of PR-4Pi algorithm on various conditions).

Further, we tested localizations on 40-nm bead data with a photon range of 470 to 5700 per objective lens and a background range of 0–4 photons per pixel (see Supplementary Fig. 16 for theoretical precisions at various photon and background levels). Being consistent with our simulated results, PR-4Pi pin-points the 3D position of a single bead with improved precision (1–3.5 fold within 2-µm axial range, quantified from localization results of 17 bead datasets) and bias than contrast-based method (Supplementary Figs. 17–18). We found the bias caused by inaccurate PSF model is rather random between different beads instead of consistent, and therefore impedes the possibility of calibrate a systematic bias for post-analysis correction (Fig. 2E and Supplementary Figs. 4–5). Agreeing with simulation results, PR-4Pi with 11 estimation parameters (independent background and intensity estimations for each quadrant, Supplementary Note 4) worsens the axial localization precision at near focus region, while, PR-4Pi with five estimation parameters (single background and single intensity estimations for all quadrants, Supplementary Note 4) worsens the axial localization bias at out-of-focus region (Supplementary Figs. 17–18). Therefore, we propose to use PR-4Pi with five parameters when the axial localization range is less than 1.2 µm.

### Continuous cavity phase measurement

Another key feature in 4Pi/interferometric systems is the cavity phase drift. The cavity phase describes the OPD between the two interference arms when the emitter is in focus at the common focal plane of the two objectives. The cavity phase, $\varphi _0$, modulates with path length fluctuations and the changes of refractive indices of materials which photons travel through along the interference arms (~600 mm for each arm in our system). In turn, cavity phase drift changes 4PiPSF from one pattern to another rapidly. We found the fluctuation of the room temperature (RT) lead to obvious drift in $\varphi _0$ (Supplementary Note 7 and Supplementary Fig. 19). The cavity phase drift (i.e., $\varphi _0$ drift) is different from sample drift. It is a unique challenge for interferometric systems. In comparison with commonly observed sample axial drift which would make in-focused object out of focus, the cavity phase drift simply shifts the energy distribution between the four detected quadrants while an in-focus emitter remains in-focus. Here we examine the properties of cavity phase and propose a cavity-phase drift-correction method.

The cavity phase drift, left uncorrected, degrades the axial resolution achievable in a 4Pi systems and creates image artifacts in the final reconstruction. We found a 0.04 rad drift per second will cause noticeable axial-resolution deterioration in 10 s (equivalent to ~18 nm axial drift, Supplementary Fig. 20), mandating a correction per 10 s to avoid resolution deterioration. This is especially challenging for the previous W-4PiSMSN method as the number of emitters collected within 10 s is not sufficient to correctly perform, ridge-finding, phase unwrapping and subsequent drift correction^12^. In contrast, pupil-based method allows bypassing these problems by first, no longer relying on phase unwrapping to recover axial positions and second, automatically compensating the cavity phase drift during the regression step (Supplementary Note 4 and Supplementary Figs. 1, 21).

We are able to obtain reliable calibration of $\varphi _0$ from each 10-s data batch, so that a unique PR-4PiPSF model for a specific segment of time was generated to account for the $\varphi _0$ drift. This constricted the axial-resolution deterioration within 5 nm on average (Supplementary Fig. 20). In short, within a short segment of blinking dataset, total (or interference) phase $\varphi$ and shape metric $\sigma _s$ of each emitter were measured. Assuming linear dependences of $\varphi$ with the axial position of the molecule as well as the shape metric $\sigma _s$ within a 2π range, the cavity phase $\varphi _0$ was estimated as the averaged interference phase $\varphi$ at the common focus plane of the two objectives. For single-section imaging, assuming a smooth change of the cavity phase during imaging, the estimated $\varphi _0$ over time were interpolated with a smooth spline to eliminate abrupt transitions generated by estimation uncertainties and the interpolated values were incorporated into the PSF model during the regression step (Supplementary Note 4 and Supplementary Fig. 1).

For data acquired at multiple optical sections for large volumetric 3D imaging, the scanning of the specimen to image different optical sections in the axial direction also changes the cavity phase. Assuming the scanning step size $d$ is a constant during the data acquisition, this additional phase can be calculated from,

8 $$\varphi _d = \frac{{4\pi {\mathrm{n}}_{{\mathrm{imm}}}dN_{{\mathrm{step}}}}}{{\lambda _0}}\left( {1 - \frac{{n_{{\mathrm{med}}}^2}}{{n_{{\mathrm{imm}}}^2}}} \right),$$

where $\lambda _0$ is the emission wavelength in air, and $N_{{\mathrm{step}}}$ is the step number, which is indexed from 0. We would like to note that when the refractive index of the immersion media $n_{{\mathrm{imm}}}$ matches the one of the sample media $n_{{\mathrm{med}}}$, sample scanning in axial direction no longer causes cavity phase change. Equation 8 allows us to quantitatively connect the cavity phases obtained at different optical sections and therefore, results in a continuous phase curve which improves the robustness of cavity phase calibration (Supplementary Fig. 1). Together, we are able to calibrate cavity phase based on short single-molecule blinking dataset (~10 s) with high consistency across multiple optical sections throughout a cell. We found that for both single-section and multi-section imaging the calibrated curves match well with the estimated $\varphi _0$ and are robust to the estimation noise (Supplementary Note 4 and Supplementary Fig. 1).

We tested the reliability of our cavity phase measurement by localizing bead images at various $z$ positions (Supplementary Fig. 22). An incorrect cavity phase can result in nearly 50% ghost images and when using an ideal-4PiPSF model (Supplementary Fig. 3), in spite of cavity phase values, the smallest ghost-image percentage is nearly 20% of the entire localizations. In contrast, we found that using a correctly retrieved cavity phase together with PR-4Pi algorithm, the percentage of ghost images is limited to 5–10% for both bead and cell imaging. For imaging in biological specimens, additional ghost reduction procedures further reduce it to ~2% (Supplementary Note 4, Supplementary Fig. 1 and Supplementary Table 1).

### Imaging of various cellular structures

Next, we applied the developed PR-4Pi localization algorithm to resolve a variety of cellular structures and in vitro specimens using fluorescent labels with different photon budgets. For single optical-section data, a total of 40–360 distinct PR-4PiPSFs, each representing the concurrent state during a 10 s imaging period, were generated for localization analysis (Supplementary Fig. 1). For multi-section volumetric 4Pi-SMSN imaging, 120–320 distinct PR-4PiPSFs (10 s per PSF) were used to obtain 3–4 optical volumes spaced 500–800 nm apart (Supplementary Fig. 1). These time dependent 4PiPSFs allow capturing the dynamic states of the interferometric system with high accuracy, and therefore pin-pointing single-molecule interferometric patterns with high precision and fidelity.

**Fig. 3:**
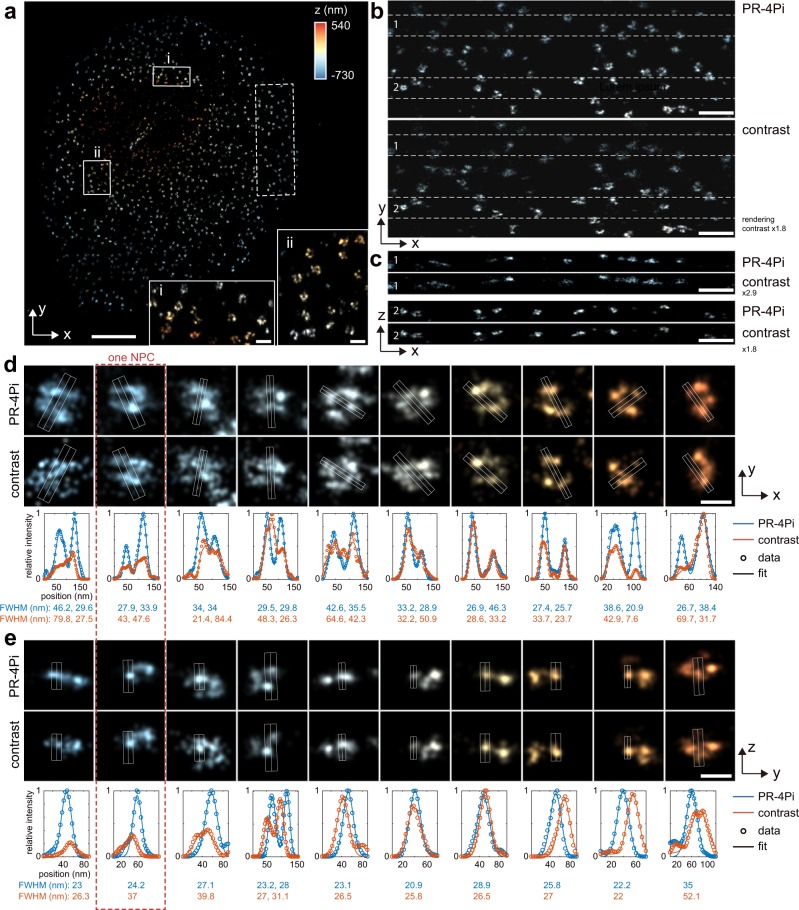
SMSN reconstruction of immune-labeled Nup98 protein in COS-7 cells. **a** Lateral view of SMSN reconstruction from the PR-4Pi method. **b** 700-nm thick *x*–y slice of selected region in **a** (white dash box). **c** 300-nm thick *x*–*z* slices of selected regions in **b** (between white dash lines). **d**, **e** Selected single nuclear pore complexes (NPC) in the *x*–*y* plane and *y*–*z* plane and their profiles along the central line, obtained by averaging the pixel values within the white box along the orthogonal dimension of the central line. Each peak was fitted by a 1D Gaussian with a width of $\sigma$, and the FWHM was calculated from 2.355$\sigma$. One column as indicated by the red dash box shows the same NPC in its lateral and axial views. The sample was imaged at a single-optical section with 90,000 frames under epi illumination. Scale bar: 2 µm in **a**, 200 nm in inlets of **a**, 500 nm in **b**, **c**, 100 nm in **d**, **e**.

**Fig. 4:**
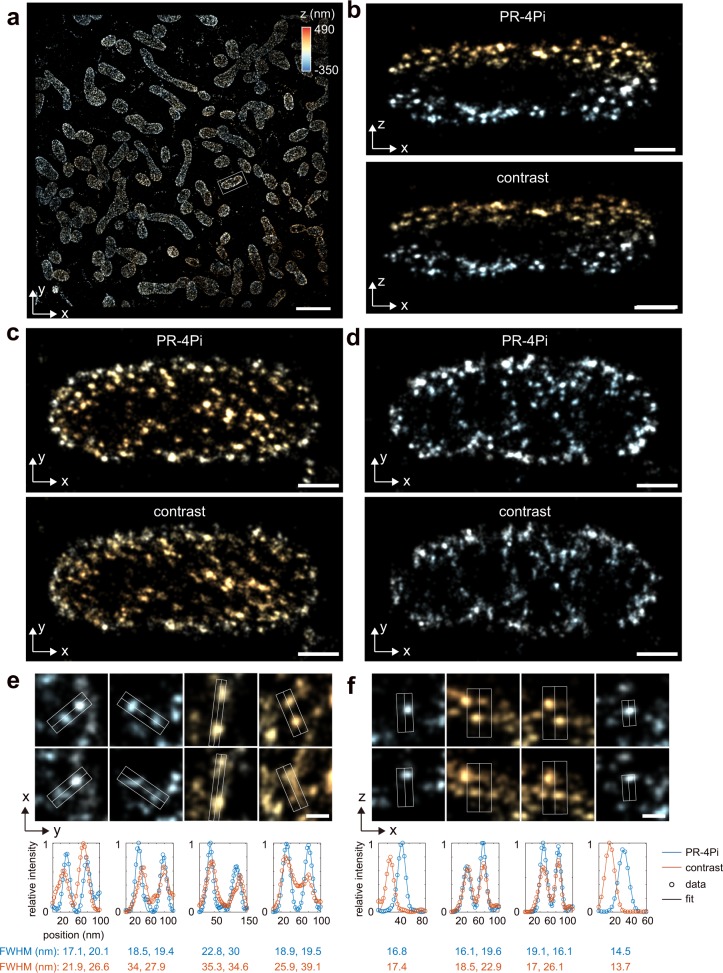
SMSN reconstruction of immune-labeled TOM20 protein in COS-7 cells. **a** Lateral view of SMSN reconstruction from PR-4Pi method. **b**, **c** 200-nm thick *x*–*z* and *x*–*y* slices of one mitochondria in **a** (white box), reconstructed from PR-4Pi and contrast methods. **d**, **e** Selected single clusters in the *x*–*y* plane and *x*–*z* plane and their profiles along the central line, obtained by averaging the pixel values within the white box along the orthogonal dimension of the central line. Each profile was fitted by a 1D Gaussian with a width of $\sigma$, and the FWHM was calculated from 2.355$\sigma$. The sample was imaged at a single-optical section with 180,000 frames under epi illumination. Scale bars: 2 µm in **a**, 200 nm in **b**–**d**, 50 nm in **e**, **f**.

We imaged immune-labeled Nup98 protein at the top nucleus envelop in COS-7 cells. From profiles of multiple nucleus pore complexes (NPC), we found that the PR-4Pi improves the sharpness of resolved structure in both lateral and axial directions (Fig. 3). We observe this improvement is more obvious with increased axial distance from the focal plane (e.g., localizations with blue color, Fig. 3) especially along the lateral dimension. This improved precision as well as its dependence on the axial position of a single molecule is in agreement with our simulation results and experimental results based on beads (Fig. 2, Supplementary Figs. 4–7). Similarly, the super-resolution reconstruction of immune-labeled TOM20 protein in COS-7 cells also shows improved lateral sharpness of resolved clusters using PR-4Pi (example profiles and quantifications are shown in Fig. 4). In comparison, contrast-based method shows increased blurriness in one of the lateral dimensions both above and below the focal plane (*z* = 0 nm), which is caused by the lateral stretching of the incoherent astigmatism PSFs at out-of-focus regions^21^ (validations of drift-correction performance is shown in Supplementary Fig. 23). Importantly, PR-4Pi also results in higher number of localizations at the out-of-focus region because of its realistic modeling of the 4Pi emission patterns (~80% more localizations for Nup98 within the axial range of −700 to −300 nm, blue color in Fig. 3).

To evaluate the performance of PR-4Pi for low photon budget emitters such as photo-switchable/convertible proteins, we imaged purified mEos3.2 proteins on coverslips (Supplementary Fig. 24). The median photon count of localized mEos3.2 emission events is 358 ± 140 (median ± s.t.d.) per objective with a median background count of 1.6 ± 0.5 photon (median ± s.t.d.) per pixel. At such low photon condition, we found that even though most localized emitters are in focus, PR-4Pi still shows improved localization precision in both axial and lateral dimensions (10% (*n* = 28) and 22% (*n* = 40), Supplementary Fig. 25, narrower profiles in lateral and axial direction, examples are shown in Supplementary Fig. 24). This suggests PR-4Pi is robust for imaging single-molecule probes with low photon budget.

To provide quantitative measurements on these resolved volumes, we measured the full width at half maximum (FWHM) from Gaussian fit of 30 to 70 profiles of isolated clusters from each of the reconstructed images (Supplementary Fig. 25). We found that PR-4Pi localization of mEos3.2 on cover glass approaches the equivalent resolution predicted from the CRLB outputs of localized emission events. Assuming that each cluster represents a single mEos3.2 molecules with a diameter of ~5 nm, well below the CRLB predicated resolution of PR-4Pi results (*xy*: 27.7 ± 5 nm, *z*: 22.4 ± 5.6 nm, median ± s.d., *n* = 88056 (number of localizations)), the measured FWHM values (*xy*: 29.7 ± 3 nm (*n* = 28), *z*: 24.7 ± 2 nm (*n* = 40), median ± s.d.) of PR-4Pi localization of mEos3.2 suggest that the achieved 3D resolution is mainly limited by the low photon count emitted by the fluorescent protein. For PR-4Pi reconstructions of TOM20 and Nup98, we found the measured FWHMs are much larger than the predicated resolutions (FWHMs) from CRLB (Supplementary Fig. 25). This discrepancy can be caused by two potential reasons: each cluster may contain multiple single molecules and the linker size using primary and secondary antibodies is larger than the achievable resolution. As for the TOM20 reconstruction, assuming a linker size^22^ of ~17.5 nm, we found that the FWHMs (*xy*: 17.1 ± 3.3 nm (*n* = 58), *z*: 16.1 ± 2.5 nm (*n* = 66), median ± s.d.) of individual clusters from PR-4Pi are close to the combined antibody size, suggesting that the 3D resolution of PR-4Pi achieved resolving TOM20 clusters is mainly limited by the size of labeling antibodies. We further quantified the resolution of PR-4Pi reconstructed images using the recently published method based on decorrelation analysis^23^ (Supplementary Fig. 26). The resolution is calculated by 2 × SMSN pixel size/maximum frequency^23^, where SMSN pixel size is the pixel size of the SMSN reconstructed images. We found that the resolution obtained from decorrelation analysis is slightly smaller than the FWHMs from CRLB (Supplementary Fig. 25), which may be caused by the difference in resolution definitions between the two methods. For all specimens quantified with decorrelation analysis, axial cross sections achieved 3–5 nm smaller resolution than that of the lateral cross sections (Supplementary Fig. 26).

## Discussion

In this work, we developed a coherent, phase-retrieved pupil based single-molecule localization algorithm for 4Pi-SMSN systems. We demonstrated PR-4PiPSF allows us to accurately model the complex single-molecule emission patterns generated from 4Pi/interferometric single-molecule imaging systems and subsequently allows us to fully extract the information carried by the detected photons. Comparing with existing 4Pi single-molecule localization algorithms, we demonstrated that PR-4Pi allows further improvement on both precision and bias in all three dimensions. We demonstrated the performance of PR-4Pi in a variety of cellular structures and in vitro specimens by resolving mitochondria networks and nuclear pore complexes in mammalian cells and individual fluorescent proteins in vitro.

The demonstrated ability to fully extract molecular position information generated by 4Pi single-molecule systems could potentially enable high-precision localization with low photon budget (150–600 photons per emission per objective), a typical condition for live cell single-molecule experiments with fluorescent proteins and high speed acquisition^20,24^. In synergy with the rapid development of novel probes^25–27^, sample preparation techniques^28,29^, labeling methods^30,31^ and PSF engineering methods^22,32^, we hope this approach allows efficient use of detected photons to overcome the practical barriers of achievable resolution in super-resolution experiments in both fixed and living specimens.

## Methods

### Sample preparation

Before single-molecule imaging, round coverslip (25-mm diameter) containing immune-stained COS-7 cells was incubated with 200 µL bead dilution, containing 100 nm crimson bead (custom-designed, Invitrogen) diluted to 1:10^6^ in PBS, for 10 min. Then the coverslip was washed three times with PBS and placed on a custom-made sample holder. Then 150 µL imaging buffer (10% (w/v) glucose in 50 mM Tris (JT4109-02, VWR), 50 mM NaCl (S271-500, Fisher Scientific), 10 mM MEA (M6500-25G, Sigma-Aldrich), 50 mM BME (M3148-25ML, Sigma-Aldrich), 2 mM COT (138924-1 G, Sigma-Aldrich), 2.5 mM PCA (37580-25G-F, Sigma-Aldrich), and 50 nM PCD (P8279-25UN, Sigma-Aldrich), pH 8.0) was added on top of the coverslip. Then a cleaned coverslip of the same size was carefully placed on top of it and the excessive buffer was removed with Kimwipes. The sample was sealed with two-component silicone sealant (Picodent Twinsil, Picodent, Germany).

For beads imaging, a cleaned round coverslip (25-mm diameter) was incubated with 200 µL bead dilution, containing 40 nm dark red bead (F8789, Invitrogen) diluted to 1:10^7^ in PBS, for 10 min. Then the sample was rinsed with PBS, drained and placed on a custom-made sample holder. Then 10 µL PBS was added on top of the coverslip and a second pre-cleaned coverslip was placed on top and the sample was sealed with two-component silicone sealant (Picodent Twinsil, Picodent, Germany).

### Immunofluorescence labeling of TOM20

COS-7 cells (CRL-1651, ATCC) were seeded on 25-mm diameter coverslips (CSHP-No1.5-25, Bioscience Tools, San Diego, CA) 1–2 days before immunofluorescence labeling. Cells were first rinsed one time with pre-warmed (at 37 °C) phosphate buffered saline (PBS, 806552-500 ML, Sigma-Aldrich) and then fixed for 15 min at RT with pre-warmed (at 37 °C) 3% paraformaldehyde (PFA, 15710, Electron Microscopy Sciences, Hatfield, PA) and 0.5% glutaraldehyde (GA, Electron Microscopy Sciences, 16019, Hatfield, PA) in PBS. Cells were then washed twice with PBS and treated for 10 min in freshly prepared fluorescence quenching buffer (0.1% sodium borohydride (NaBH_4_, 452882-25 G, Sigma-Aldrich) in PBS). After fluorescence quenching, cells were washed three times with PBS and treated for 10 min with 10 mM Tris (pH 7.3, JT4109-02, VWR). Cells were then rinsed three times with PBS and permeabilized with blocking buffer (3% bovine serum albumin (BSA, 001-000-162, Jackson ImmunoResearch) and 0.2% Triton X-100 (X100, Sigma-Aldrich) in PBS) for 30 min, gently rocking at RT. After blocking, cells were incubated with anti-TOMM20 primary antibody (sc-11415, Santa Cruz Biotechnology), diluted to 1:500 in 1% BSA and 0.2% Triton X-100 in PBS, at 4 °C for overnight. Cells were then washed three times each time for 10–15 min with wash buffer (0.05% Triton X-100 in PBS) and incubated with secondary antibody conjugated with Alexa Fluor 647 (A21245, Life Technologies, Grand Island, NY), diluted to 1:500 in 1% BSA and 0.2% Triton X-100 in PBS, at RT for 4–5 h. After incubation with secondary antibody, cells were washed three times each time for 10–15 min with wash buffer. And then cells were post-fixed with 4% PFA in PBS for 10 min. After post-fixation, cells were rinsed three times with PBS and stored in PBS at 4 °C until they were imaged.

### Immunofluorescence labeling of Nup98

COS-7 cell were seeded on 25-mm diameter coverslips 1–2 days before immunofluorescence labeling. Cells were first rinsed one time with pre-warmed (at 37 °C) PBS and then extracted for 60 s at RT with pre-warmed (at 37 °C) 0.6% PFA and 0.1% GA and 0.25% Triton X-100 in PBS. Cells were then fixed for 15 min at RT with pre-warmed (at 37 °C) 3% PFA and 0.1% GA in PBS. After fixation, cells were washed twice with PBS and treated for 10 min in freshly prepared fluorescence quenching buffer (0.1% NaBH_4_ in PBS). After fluorescence quenching, cells were washed three times with PBS and treated for 10 min with 10 mM Tris (pH 7.3). Cells were then rinsed three times with PBS and permeabilized with blocking buffer (3% BSA and 0.2% Triton X-100 in PBS) for 30 min, gently rocking at RT. After blocking, cells were incubated with anti-NUP98 primary antibody (2598, Cell Signaling Technology), diluted to 1:200 in 1% BSA and 0.2% Triton X-100 in PBS, at 4 °C for overnight. Cells were then washed three times each time for 10–15 min with wash buffer (0.05% Triton X-100 in PBS) and incubated with secondary antibody conjugated with Alexa Fluor 647 (A21245, Life Technologies, Grand Island, NY), diluted to 1:200 in 1% BSA and 0.2% Triton X-100 in PBS, at RT for 4–5 h. After incubation with secondary antibody, cells were washed three times each time for 10–15 min with wash buffer. And then cells were post-fixed with 4% PFA in PBS for 10 min. After post-fixation, cells were rinsed three times with PBS and stored in PBS at 4 °C until they were imaged.

### Preparation of mEos3.2 on cover glass

First 25-mm diameter coverslips were cleaned as follows: first sequentially sonicated in nanopure water, 100% ethanol (04-355-223, Fisher Scientific) and 1 M potassium hydroxide (KOH, P250-500, Fisher Scientific), each for 10–15 min; then thoroughly rinsed six times with nanopure water, and dried on lens paper and stored in parafilm sealed 100-mm petri dish. Then, cleaned coverslip was incubated with 1 mg/mL biotin-BSA (29130, Thermo Scientific) in nanopure water at RT for 5 h. Coverslip was then washed three times with PBS and incubated with 0.1 mg/mL streptavidin (85878-1MG, Sigma-Aldrich) in nanopure water at RT for 1 h. Coverslip was then washed three times PBS and incubated with purified mEos3.2 (from Pollard’s lab, Yale University), diluted to 1:1000 in 0.2 mg/mL biotin-BSA in nanopure water, at RT for 15 min. After incubation with mEos3.2, coverslip was washed three times with PBS and stored in PBS at 4 °C until it was imaged.

### Data acquisition

All data were acquired on a custom-built 4Pi-SMSN microscope, constructed from the previous design^12^. In short, emitted photons were collected through two opposing high-NA oil immersion objectives (Olympus UPLSAPO 100XO, 1.4 NA) and received by a sCMOS camera (Orca-Flash4.0v2, Hamamastu, Japan). One quad-band dichroic/filter pair (FF01-446/523/600/677-25, Di01-R405/488/561/635-17.5×24, Semrock) was installed immediately after each objective for reflection of excitation lasers and initial filtering of fluorescence emission.

The SMSN data were collected with the following procedure: (1) Apply astigmatism to each DM, amplitudes of 2 and −1.5 (unit: λ/2π) for the 5th Zernike polynomials (Wyant order)^33^ were used for the upper and lower DMs respectively. (2) Collect a stack of incoherent PSF images through the upper and lower objectives independently by imaging a bead on the bottom coverslip at *z* positions from −1 to 1 µm, with a step size of 100 nm, a frame rate of 1 Hz and taking one frame per axial position. Those PSF data were used to generate phase-retrieved pupil functions of upper and lower emission paths. (3) Align the *x*, *y*, and *z* positions of both objectives by centering and focusing the beads images from those objectives, and then collect a stack of interference PSF images by imaging a bead on the bottom coverslip at *z* positions from −1 to 1 µm, with a step size of 10 nm, a frame rate of 1 Hz and taking one frame per axial position. This PSF dataset was used for the estimation of the quartz induced phase shift $\Delta \varphi _{{\mathrm{sp}}}$, emission wavelength $\lambda$, intensity ratio $I_{\mathrm{t}}$ and shape metric of infocus PSF $\sigma _{s0}$ (Supplementary Notes 1, 4). (4) Focus on a cell that took up greater than 70% of the field of view and collect 40 frames of cell images through only the lower objective at a frame rate of 10 Hz. This cell data was used for quadrant alignment (Supplementary Note 4). Steps 1 to 4 were usually repeated for each sample to ensure a realistic modeling of the 4PiPSF (see phase retrieval section in Supplementary Note 4) and an accurate alignment of the four quadrants. 40 nm dark red beads (F8789, Invitrogen) excited with a 642 nm laser were used for Alexa Fluor 647 tagged samples and the fluorescence was filtered through an emission filter (ET700/75 m, Chroma) installed on a filter wheel before the camera. And 40 nm red beads (F8793, Invitrogen) excited with a 561 nm laser were used for mEos3.2 sample and the fluorescence was filtered through an emission filter (FF01-600/52-25, Semrock) installed on a filter wheel before the camera.

For imaging of Alexa Fluor 647 tagged sample (TOM20 and Nup98): (5) Locate a region of interest and realign the objectives with a 642 nm laser (2RU-VFL-P-2000-642-B1R, MPB Communications Inc.) at an excitation intensity of 90 W cm^-2^. (6) Collect blinking data with a laser intensity of 2–7 kW cm^−2^ under epi illumination. For imaging of mEos3.2: 5) briefly illuminate with a 405 nm laser (DL-405-100, CrystaLaser) at 2 W cm^−2^ to convert a subset of molecules to their red form. (7) Collect blinking data with a 561 nm laser at 1 kW cm^−2^ and a 405 nm laser at 2 W cm^−2^ to maintain the density of emission events. For single-optical section imaging, 20,000–180,000 frames were collected. For multi-optical section imaging, the cell was scanned axially by moving the stage at a step size of 500–800 nm from the bottom to the top of the cell for 10–20 cycles, 3–4 axial planes were imaged per cycle and 2000 frames were collected per axial plane in each cycle.

Bead data for testing PR-4Pi algorithm were collected by imaging a bead on the bottom coverslip at $z$ positions from −1 to 1 µm, with a step size of 100 nm, and taking 40 frames per axial position. The bead intensity of each dataset was adjusted by varying the excitation laser power and the exposure time.

### Fisher information content of contrast method

Contrast method in this work refers to the algorithm developed in ref. ^12^ that extracts the axial positions using the Gaussian-weighted 0th central moment (M0, Supplementary Note 4 and Supplementary Table 1). In the lateral dimension, the Fisher information content of contrast method^12^ is equivalent to the one using astigmatism method on a dual-objective system^14^ (Supplementary Figs. 8–9). In the axial dimension, the contrast method operates on the center lobe of each PSF, which was extracted by multiplying the PSF with a 2D Gaussian at a width of one pixel. After multiplication, the remaining information for axial localization is 20–60%.

### Statistics and reproducibility

For testing the performance of PR-4Pi on bead imaging, 17 bead data were collected at various photon count and lateral positions sampled from the full field of view. For testing the performance of PR-4Pi on cellular structures, at least three datasets ($n\, \ge \,3$) were analyzed for each type of demonstrated structures and similar performance was observed. Statistical analyses of the bead data and cellular structures (Supplementary Figs. 17, 18, 25) were analyzed using the function *boxplot* from MATLAB (MathWorks, Natick, MA), detailed number of measurements are given in the Main Text and the figure legends.

### Reporting summary

Further information on research design is available in the Nature Research Reporting Summary linked to this article.

## Supplementary information

Supplementary Information

Supplementary Software

Supplementary Data

Description of Additional Supplementary Files

Reporting Summary

Peer Review File

## Data Availability

Data underlying the plots in Figs. 2b–e, 3d,e and Fig. 4e, f are available as Excel files and via Figshare in Supplementary Data. Example test data for using PR-4Pi algorithm are also available in the Supplementary Software packages. Other data that support the findings of this study are available from the corresponding author upon request

## Code availability

The analyzing algorithms for 4Pi-SMSN data are implemented as a MATLAB class, named *SR4pi_demo*, which provides localization methods using PR-4Pi algorithm and contrast algorithm. An example script on using *SR4pi_demo* class is available in Supplementary Software and further updates will be made available at https://github.com/HuanglabPurdue/PR-4Pi.

## References

[1] Hell S, Stelzer EHK (1992). Properties of a 4Pi confocal fluorescence microscope. J. Opt. Soc. Am. A.

[2] Gugel H (2004). Cooperative 4Pi excitation and detection yields sevenfold sharper optical sections in live-cell microscopy. Biophys. J..

[3] Bewersdorf J, Bennett BT, Knight KL (2006). H2AX chromatin structures and their response to DNA damage revealed by 4Pi microscopy. Proc. Natl Acad. Sci. USA..

[4] Gustafsson, M. G. L., Agard, D. A. & Sedat, J. W. Sevenfold improvement of axial resolution in 3D wide-field microscopy using two objective lenses. in *Three-Dimensional Microscopy: Image 454 Acquisition and Processing II* **2412**, 147–156 (1995).

[5] Gustafsson MGL, Agard DA, Sedat JW (1999). I5M: 3D widefield light microscopy with better than 100 nm axial resolution. J. Microsc..

[6] Shao L (2008). I5S: Wide-field light microscopy with 100-nm-scale resolution in three dimensions. Biophys. J..

[7] Nagorni M, Hell SW (2001). Coherent use of opposing lenses for axial resolution increase II Power and limitation of nonlinear image restoration. J. Opt. Soc. Am. A.

[8] Shtengel G (2009). Interferometric fluorescent super-resolution microscopy resolves 3D cellular ultrastructure. Proc. Natl Acad. Sci. USA..

[9] Brown TA (2011). Superresolution fluorescence imaging of mitochondrial nucleoids reveals their spatial range, limits, and membrane interaction. Mol. Cell. Biol..

[10] Aquino D (2011). Two-color nanoscopy of three-dimensional volumes by 4Pi detection of stochastically switched fluorophores. Nat. Methods.

[11] v. Middendorff C, Egner A, Geisler C, Hell SW, Schönle A (2008). Isotropic 3D nanoscopy based on single emitter switching. Opt. Express.

[12] Huang F (2016). Ultra-high resolution 3D imaging of whole cells. Cell.

[13] Backlund MP, Shechtman Y, Walsworth RL (2018). Fundamental precision bounds for three-dimensional optical localization microscopy with poisson statistics. Phys. Rev. Lett..

[14] Xu K, Babcock HP, Zhuang X (2012). Dual-objective STORM reveals three-dimensional filament organization in the actin cytoskeleton. Nat. Methods.

[15] Hanser BM, Gustafsson MGL, Agard DA, Sedat JW (2004). Phase-retrieved pupil functions in wide-field fluorescence microscopy. J. Microsc..

[16] Liu S, Kromann EB, Krueger WD, Bewersdorf J, Lidke KA (2013). Three dimensional single molecule localization using a phase retrieved pupil function. Opt. Express.

[17] Zhang P (2018). Analyzing complex single-molecule emission patterns with deep learning. Nat. Methods.

[18] Sengupta, S. K. & Kay, S. M. *Fundamentals of Statistical Signal Processing: Estimation Theory*. *Technometrics* **37** (Prentice Hall, 1995).

[19] Ober RJ, Ram S, Ward ES (2004). Localization accuracy in single-molecule microscopy. Biophys. J..

[20] Huang F (2013). Video-rate nanoscopy using sCMOS camera-specific single-molecule localization algorithms. Nat. Methods.

[21] Von Diezmann A, Shechtman Y, Moerner WE (2017). Three-dimensional localization of single molecules for super-resolution imaging and single-particle tracking. Chem. Rev..

[22] Aristov A, Lelandais B, Rensen E, Zimmer C (2018). ZOLA-3D allows flexible 3D localization microscopy over an adjustable axial range. Nat. Commun.

[23] Descloux A, Grußmayer KS, Radenovic A (2019). Parameter-free image resolution estimation based on decorrelation analysis. Nat. Methods.

[24] Marsh RJ (2018). Artifact-free high-density localization microscopy analysis. Nat. Methods.

[25] Grimm JB (2016). Bright photoactivatable fluorophores for single-molecule imaging. Nat. Methods.

[26] Uno SN (2014). A spontaneously blinking fluorophore based on intramolecular spirocyclization for live-cell super-resolution imaging. Nat. Chem..

[27] Takakura H (2017). Long time-lapse nanoscopy with spontaneously blinking membrane probes. Nat. Biotechnol..

[28] Chen F, Tillberg PW, Boyden ES (2015). Expansion micrsocopy. Science.

[29] Chozinski TJ (2016). Expansion microscopy with conventional antibodies and fluorescent proteins. Nat. Methods.

[30] Jungmann R (2014). Multiplexed 3D cellular super-resolution imaging with DNA-PAINT and exchange-PAINT. Nat. Methods.

[31] Pallikkuth S (2018). Sequential super-resolution imaging using DNA strand displacement. PLos ONE.

[32] Shechtman Y, Weiss LE, Backer AS, Sahl SJ, Moerner WE (2015). Precise three-dimensional scan-free multiple-particle tracking over large axial ranges with tetrapod point spread functions. Nano Lett..

[33] Wyant JC, Creath K (1992). Basic wavefront aberration theory. Appl. Opt. Opt. Eng..

