# Author Correction: Enhanced 4Pi single-molecule localization microscopy with coherent pupil based localization

**DOI:** 10.1038/s42003-020-1020-3

**Published:** 2020-05-29

**Authors:** Sheng Liu, Fang Huang

**Affiliations:** 1 Weldon School of Biomedical Engineering, Purdue University, West Lafayette, IN USA; 2 Purdue Institute for Integrative Neuroscience, Purdue University, West Lafayette, IN USA; 3 Purdue Institute of Inflammation, Immunology and Infectious Disease, Purdue University, West Lafayette, IN USA

**Keywords:** Optical imaging, Super-resolution microscopy

Correction to: *Communications Biology* 10.1038/s42003-020-0908-2, published online 8 May 2020.

In the original published version of the article, the link to the Figshare dataset referred to in the Data availability statement and the Supplementary Information was missing. The link is 10.6084/m9.figshare.11987448.v1. The errors have been corrected in the HTML and PDF versions of the article and in the ‘Description of Additional Supplementary Files’ PDF.

