# Supplementary material for: Enhanced 4Pi single-molecule localization microscopy with coherent pupil based localization
Source: Commun Biol. 2020 May 8;3:220. doi: 10.1038/s42003-020-0908-2 (PMC7210115; doi:10.1038/s42003-020-0908-2)
Supplement: Supplementary file 1 — Supplementary Information [file 42003_2020_908_MOESM1_ESM.pdf]

Table of Contents

Supplementary Figures 3

Supplementary Figure 1. Flow chart of PR-4Pi localization algorithm on SMSN data. 3

Supplementary Figure 2. Effect of coherence strength and intensity ratio on lateral and axial localization precisions. 6

Supplementary Figure 3. Comparison of phase retrieved 4PiPSF and ideal 4PiPSF with acquired 4Pi emission patterns from a 40 nm bead. 7

Supplementary Figure 4. Comparison of localization results using various PSF models on experimental bead data with various photon counts. 8

Supplementary Figure 5. Comparisons of localization results using contrast and PR-4Pi methods on 9 different bead datasets. 9

Supplementary Figure 6. Comparison of various localization methods with CRLB on simulated 4PiPSF data *without* astigmatism modification. 10

Supplementary Figure 7. Comparison of various localization methods with CRLB on simulated 4PiPSF data with astigmatism modification. 11

Supplementary Figure 8. Lateral distributions of the Fisher information content per pixel in x, y and z estimations based on PSF models with or without astigmatism modifications. 12

Supplementary Figure 9. Comparison of Fisher information and theoretical precisions from CRLB on various PSF models. 13

Supplementary Figure 10. Comparison of theoretical localization precisions from CRLB calculation of PR-4PiPSF models with and without astigmatism modifications. 14

Supplementary Figure 11. Effect of initial guess on axial localization using PR-4Pi method. 15

Supplementary Figure 12. Effect of the misalignment between the two objectives on localization deviations using PR-4Pi algorithm. 16

Supplementary Figure 13. Convergence of PR-4Pi algorithm on localization of experimental bead data with various photon count. 17

Supplementary Figure 14. PR-4PiPSF models considering index mismatch aberration (IMM). 18

Supplementary Figure 15. Comparison of linear and cubic interpolation methods on localizations of bead data. 19

Supplementary Figure 16. Localization precision at different photon and background levels. 20

Supplementary Figure 17. Ratios of absolute localization deviations (bias) between contrast and PR-4Pi methods on experimental bead data. 21

Supplementary Figure 18. Ratios of localization precision between contrast and PR-4Pi methods on experimental bead data. 22

Supplementary Figure 19. Correlation of temperature fluctuation and cavity phase drift. 23

Supplementary Figure 20. Effect of cavity phase drift on axial resolution. 24

Supplementary Figure 21. Limitations of phase unwrapping method (ridge-finding algorithm) in contrast algorithm. 25

Supplementary Figure 22. Effect of cavity phase bias on the percentage of ghost localizations. 26

Supplementary Figure 23. Localization results after drift correction with color scaled in time (batch number). 27
Supplementary Figure 24. SMSN reconstruction of mEos3.2 on coverslip. 29
Supplementary Figure 25. Cluster profile quantifications of PR-4Pi and contrast methods on various biological specimens. 30
Supplementary Figure 26. Resolution quantification of PR-4Pi on various biological specimens using decorrelation analysis 31
Supplementary Tables 32
Supplementary Table 1. Comparison of interferometric single-molecule localization microscopies 32
Supplementary Table 2. Run time of PR-4Pi algorithm. 33
Supplementary Notes 34
1. Estimations of hyper parameters in PR-4PiPSF model 34
2. Effect of intensity ratio and coherence strength 35
3. Increase of information content using interferometric PSF 35
4. PR-4Pi localization algorithm 36
4.1 Calibration of cavity phase 37
4.2 Estimation of cavity phase 38
4.3 Regression 38
4.4 Ghost reduction 40
4.5 Removal of mislocalizations 42
4.6 Drift correction 42
4.7 Rejection 43
4.8 SMSN reconstruction 43
4.9 Astigmatism modification of 4PiPSF 43
4.10 Phase retrieval 44
4.11 Quadrant alignment 44
5. Linear and cubic interpolations in PR-4Pi algorithm 45
5.1 Linear interpolation 45
5.2 Cubic interpolation 47
6. Effect of index mismatch aberration 49
7. Temperature modulation on 4PiPSFs 51
References 52

Supplementary Figures

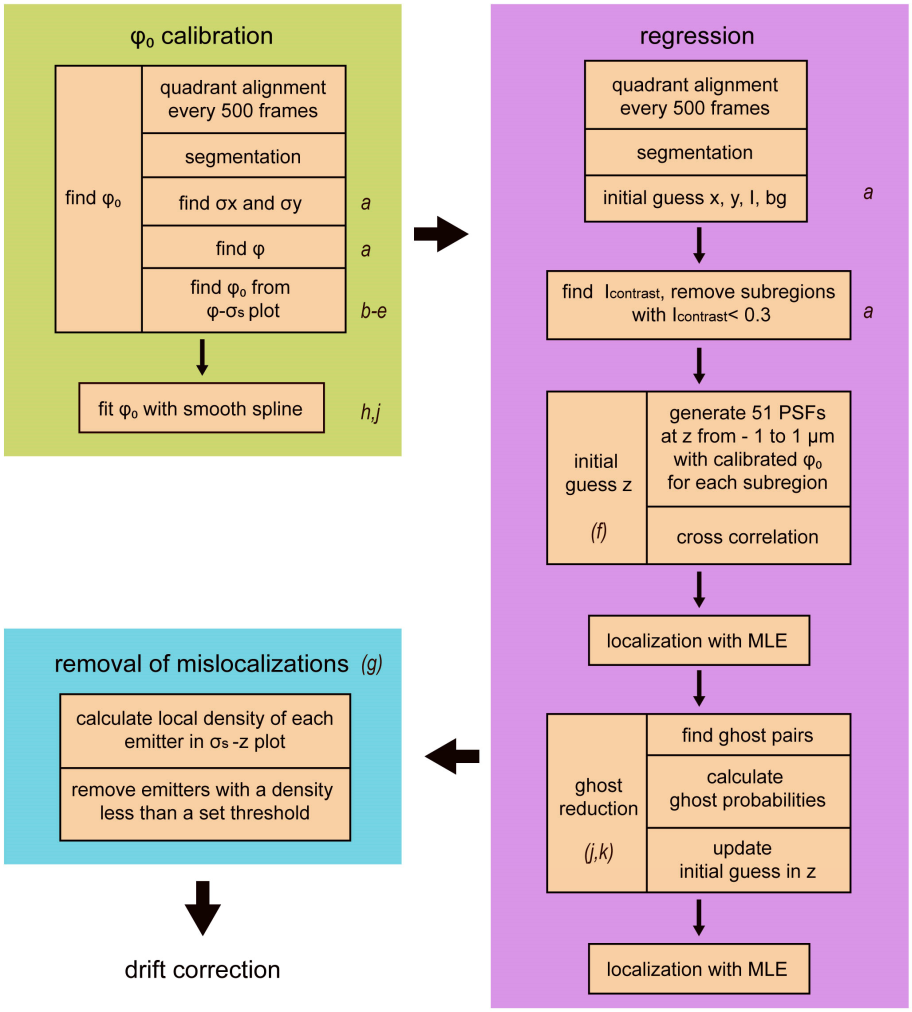

Supplementary Figure 1. Flow chart of PR-4Pi localization algorithm on SMSN data.
Steps marked with italic letters are accompanied with corresponding graphic illustrations following the flow chart.

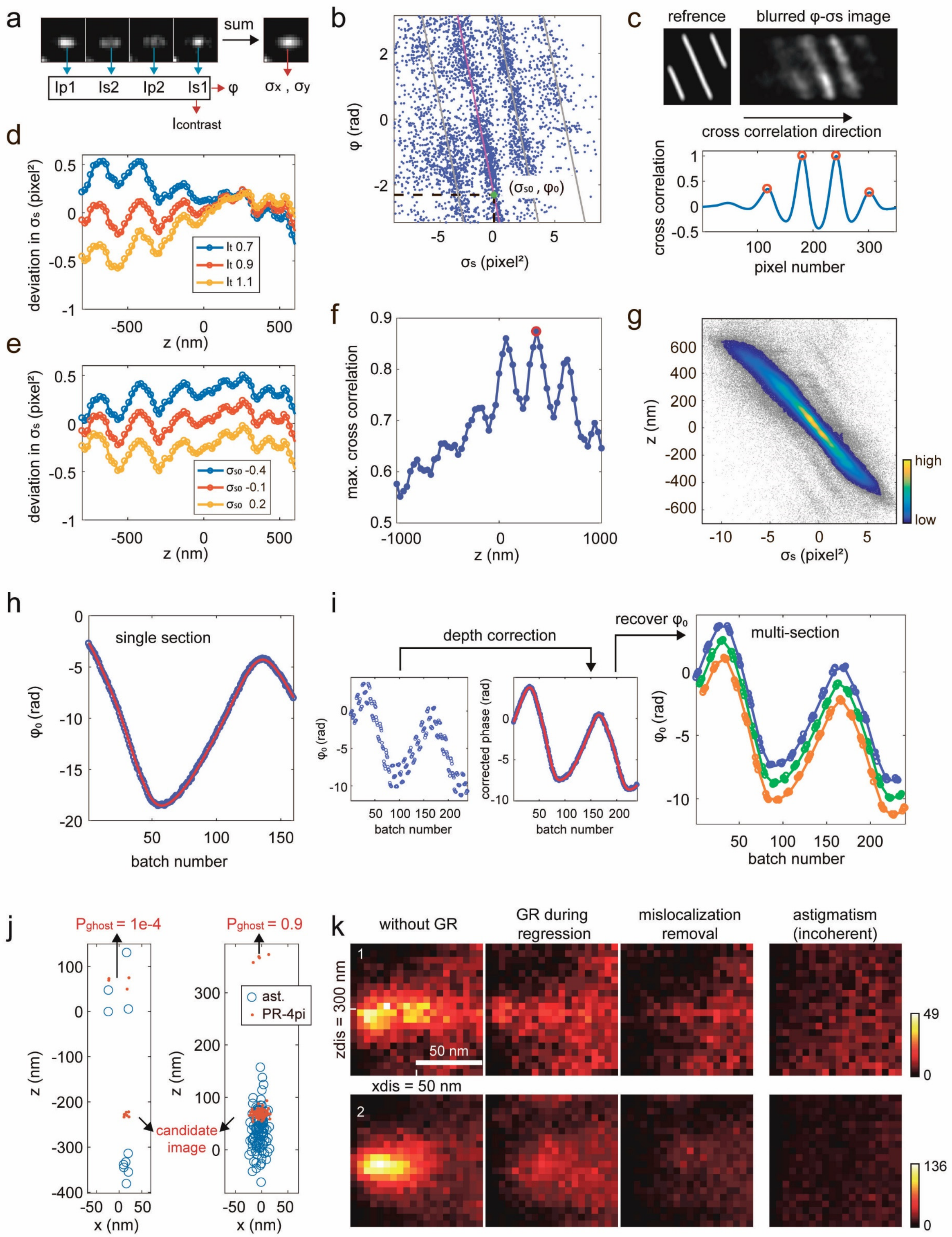
a
sum
Ip1
Is2
Ip2
Is1
φ
σx , σy
Icontrast
b
φ (rad)
(σs0 , φ0)
σs (pixel²)
c
refrence
blurred φ-σs image
cross correlation direction
cross correlation
pixel number
d
deviation in σs (pixel²)
It 0.7
It 0.9
It 1.1
z (nm)
e
deviation in σs (pixel²)
σs0 -0.4
σs0 -0.1
σs0 0.2
z (nm)
f
max. cross correlation
z (nm)
g
z (nm)
high
low
σs (pixel²)
h
φ0 (rad)
single section
batch number
i
depth correction
recover φ0
φ0 (rad)
corrected phase (rad)
batch number
multi-section
j
Pghost = 1e-4
Pghost = 0.9
ast.
PR-4pi
candidate image
z (nm)
x (nm)
k
without GR
GR during regression
mislocalization removal
astigmatism (incoherent)
zdis = 300 nm
50 nm
xdis = 50 nm
49
136

(**a**) An example of 4PiPSF-image of a single emitter, it contains PSFs from four channels (P1, S2, P2 and S1), from which the interference phase (φ) and the modulation contrast (I_{contrast}) can be estimated. And the sum of the PSFs from the four channels was used to estimate the PSF widths σ_x and σ_y (Supplementary Note 4). (**b**) Estimation of the cavity phase, φ_0. It was estimated from the line fit (magenta line) of the stripe with the highest emitter density, selected from the highest peak of the cross correlation result in (c). (**c**) Cross correlation of a reference image and a Gaussian blurred φ-σ_s image along the σ_s dimension. Peaks above a set threshold were selected (red circles) to determine the fitted line positions in the φ-σ_s plot (b). (**d**) Selection of intensity ratio (I_t). The intensity ratio was selected so that the slopes of z-σ_s plots match between PR-4PiPSF models and measured PSFs. (**e**) Selection of infocus shape metric (σ_{s0}). The infocus shape metric was selected to minimize the offset of σ_s values of PR-4PiPSF models and measured PSFs. (**f**) Cross correlation of a 4Pi bead-image with a set of library 4PiPSFs. At each axial position, first the maximum cross correlation value between the bead-image and the library 4PiPSF from each of the four channels was calculated, then the mean of the obtained four values was used to generate the curve. The optimum z position (red circle) was used as the initial z position for maximum likelihood estimation (MLE). (**g**) Removal of mislocalizations. The localizations with a local density in the σ_s-z plot higher than a set threshold were accepted (within the color stripe). The discarded localizations (gray points) were considered as mislocalizations. (**h**) Cavity phase calibration from single-optical section imaging of mitochondria (TOM20) in COS-7 cell. Cavity phase φ_0 for every data batch (10 s each) were extracted and subsequently its time evolution was obtained through interpolation (Supplementary Note 4). (**i**) Cavity phase calibration from multi-optical section imaging of mitochondria (TOM20) in COS-7 cell. The initial estimates of φ_0 were depth corrected to form a smooth phase-evolution and fitted with a smooth spline function. The actual evolution of φ_0 for each optical section was then recovered from the fitted spline. (**j**) Examples of ghost pairs after regression. Not all ghost pairs are from the same emitter, only the ghost images with high ghost probabilities (P_{ghost}) were updated or removed (Supplementary Note 4). (**k**) Density of ghost pairs before and after ghost reduction (GR) and mislocalization removal, where zdis and xdis represent the pairwise distances of localized positions in axial and lateral dimensions respectively (Supplementary Note 4). Ghost images were largely reduced by GR during the regression step and further diminished by mislocalization removal after regression. The resulting ghost density is comparable with the one under incoherent detection, which is ghost free. Incoherent PSFs were generated from the sum of the 4PiPSFs of the four channels and were localized with astigmatism method based on a Gaussian PSF model.

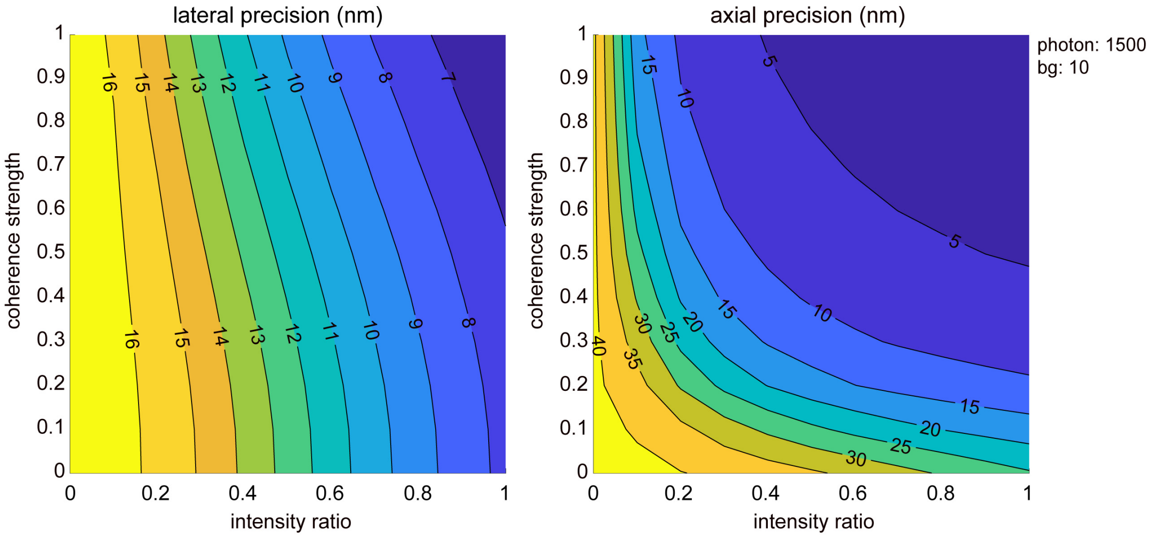

Supplementary Figure 2. Effect of coherence strength and intensity ratio on lateral and axial localization precisions.

Theoretical precisions based on CRLB (Main Text) were calculated from 4PiPSF models with various coherence strength and intensity ratios (Supplementary Note 2). The precision value under each condition was the average of 19 calculations at *z* positions uniformly distributed from -600 nm to 600 nm. The total photon and background photon used were 1500 per objective and 10 per pixel. PR-4PiPSF models were generated based on the phase retrieved pupil functions by imaging a 40 nm bead.

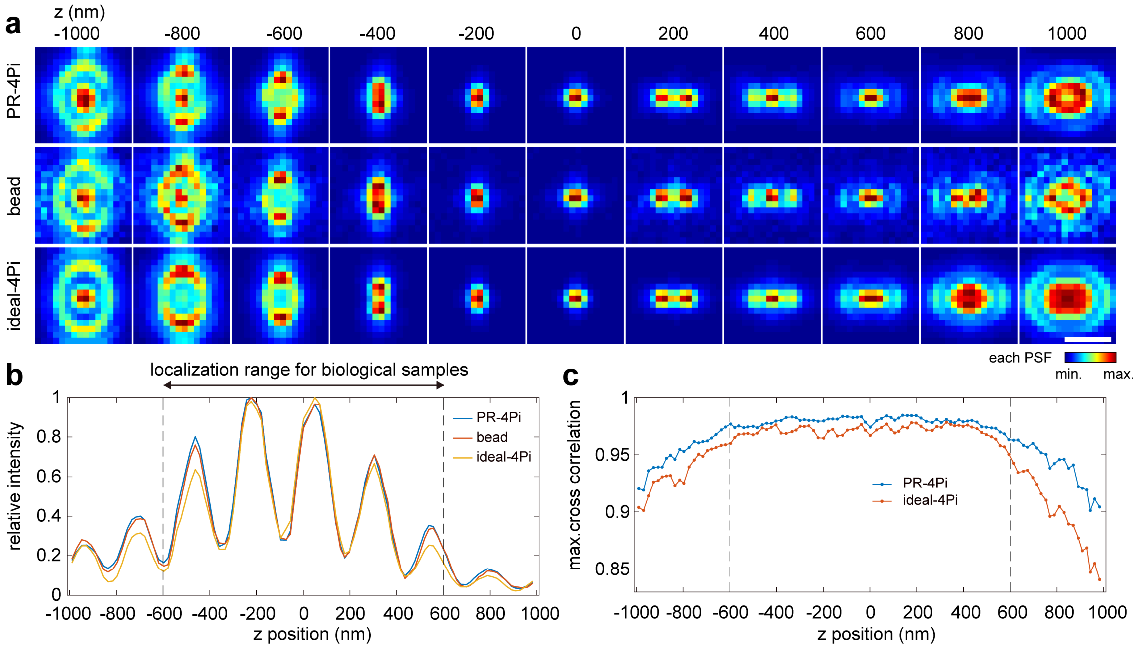

Supplementary Figure 3. Comparison of phase retrieved 4PiPSF and ideal 4PiPSF with acquired 4Pi emission patterns from a 40 nm bead.

(**a**) PSFs at channel P2 from experimental bead data, phase retrieved (PR) and theoretical ideal PSF models. Ideal-4PiPSF model assumes no aberration, transmission loss and beam splitting inequality present in the imaging system. The simulated PSF models were generated based on the localization results (x, y, z) of the bead data using corresponding PSF models to find the best match in both PR-4Pi and ideal-4Pi cases. (**b**) Modulation intensity of the center lobes (Gaussian-weighted 0th central moment) of various 4PiPSFs shown in (a) (Supplementary Note 4). (**c**) Model accuracies quantified by cross correlations of PR- and ideal-4PiPSFs with the experimentally acquired PSFs from beads. At each axial position, first the maximum cross correlation value between the PR/ideal-4PiPSF and the bead-image from each of the four channels (P1, S2, P2 and S1) was calculated, then the mean of the obtained four values was used to generate the curve. The bead data were collected with high signal to noise ratio (SNR) at z positions from -1 µm to 1 µm, with a step size of 10 nm (Methods). Scale bar: 1 µm.

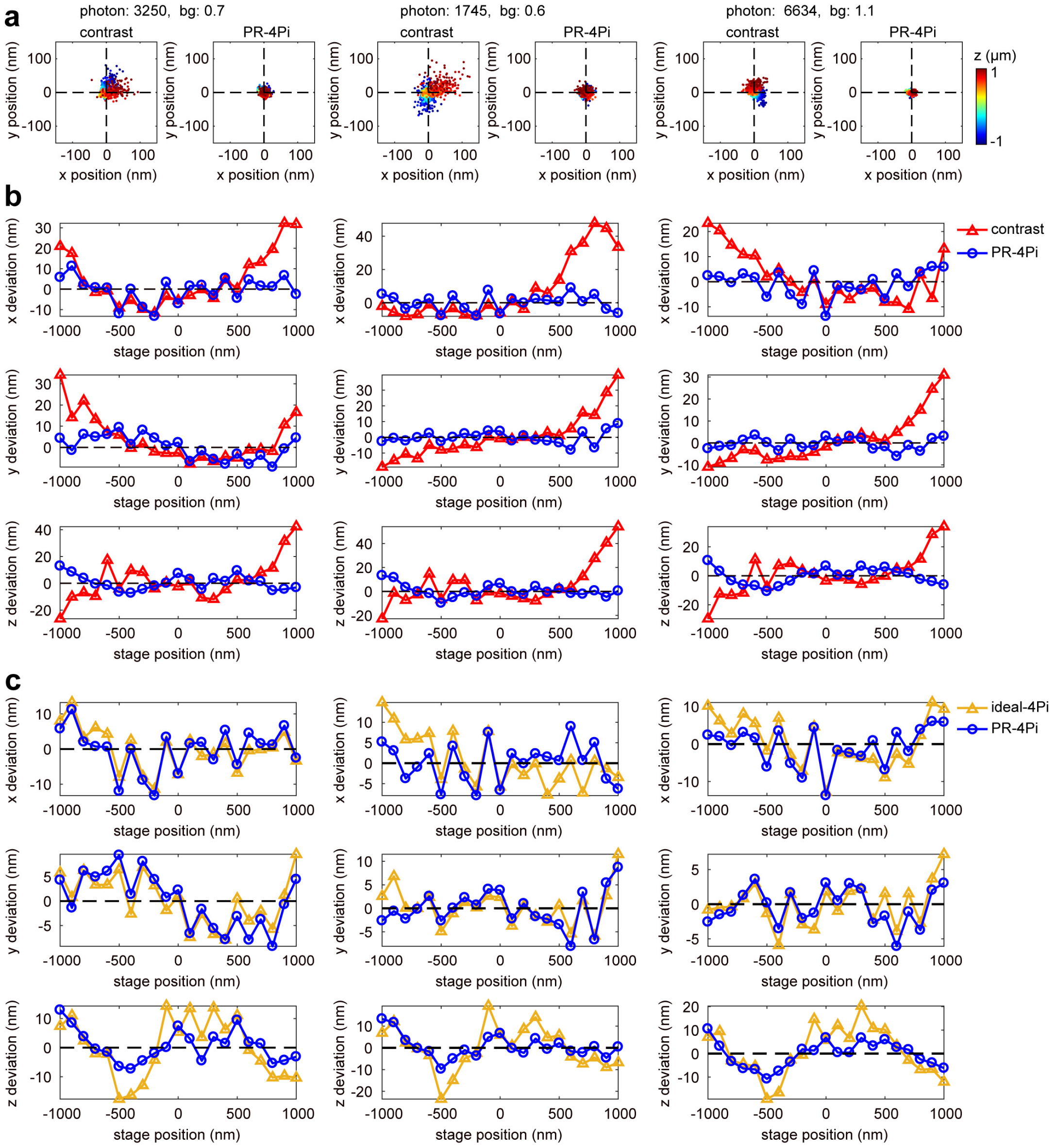

Supplementary Figure 4. Comparison of localization results using various PSF models on experimental bead data with various photon counts.
(**a**) Scatter plot of lateral localizations from an axial scan of 40 nm dark red bead from -1 μm to 1 μm relative to the common focus of two 4Pi objectives. Estimations with PR-4Pi p11 (11 parameters with independent intensity/background between four quadrants) and contrast methods were compared. Color scaled with axial positions. (**b**) Localization deviations in x, y and z from PR-4Pi p11 and contrast methods. (**c**) Localization deviations in x, y and z from PR-4Pi p11 method and ideal-4Pi p11 method that is based on ideal-4PiPSF models used unaberrated pupil functions and assumed no transmission loss and beam splitting inequality presented in the imaging system. Bead data for localization were analyzed using the same PR-4PiPSF model. Bead data for phase retrieval and localizations were collected from different beads. All bead data were collected during the same experiment and from the same bead sample. photon: median photon per objective (n=819), bg: median background per pixel (n=819).

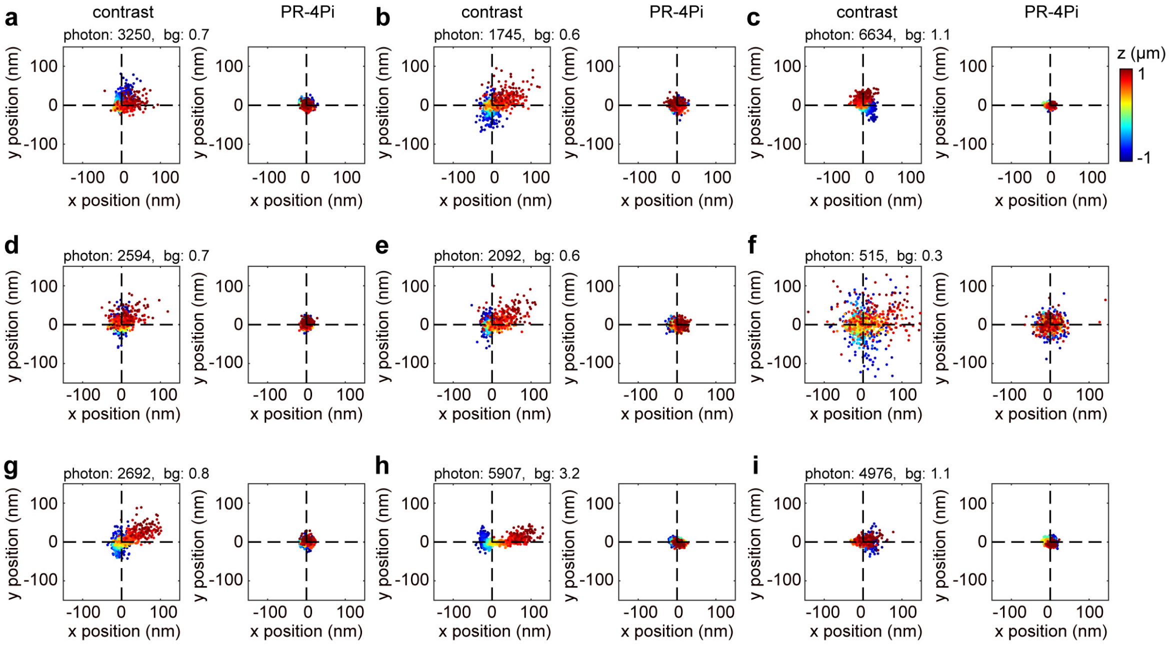

Supplementary Figure 5. Comparisons of localization results using contrast and PR-4Pi methods on 9 different bead datasets.
Estimations with PR-4Pi p11 (11 parameters with independent intensity/background between four quadrants) and contrast methods were compared. Color scaled with axial positions. Each dataset was collected by imaging a 40 nm bead at axial positions from -1 µm to 1 µm, 100 nm step size and 39 frames per step. All bead data were collected during the same experiment and from the same bead sample. photon: median photon per objective (n=819), bg: median background per pixel (n=819).

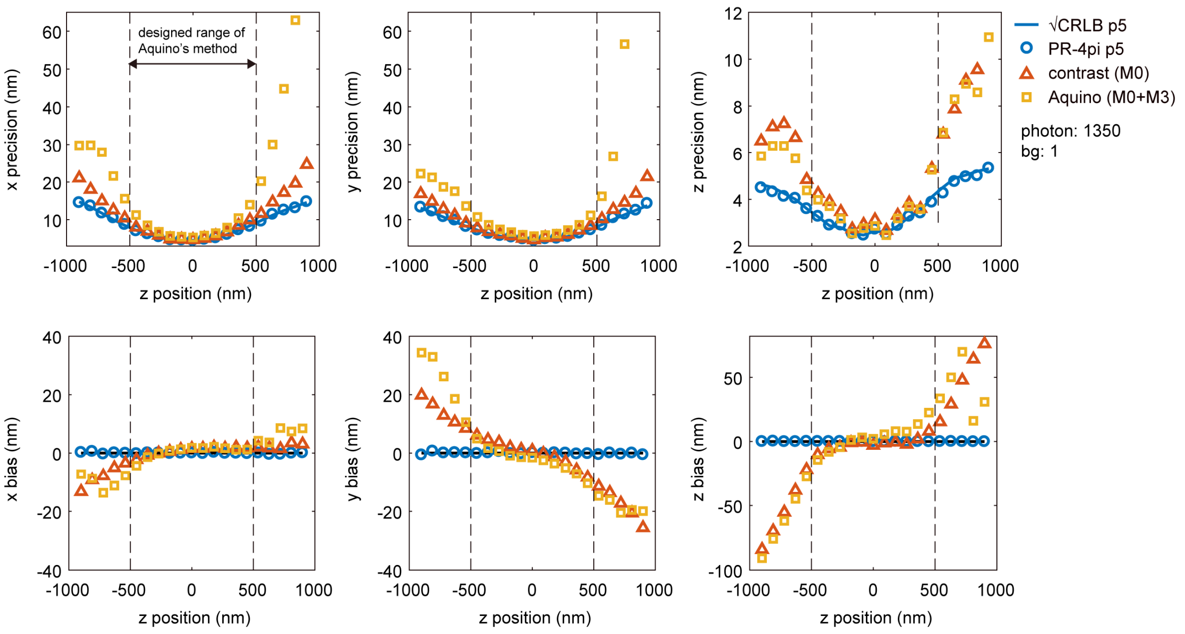

Supplementary Figure 6. Comparison of various localization methods with CRLB on simulated 4PiPSF data *without* astigmatism modification.

PR-4Pi p5 refers to the PR-4pi algorithms with fixed intensity and background between four quadrants. Contrast method refers to the algorithm developed in ref. [1] that extracts the axial positions using the Gaussian-weighted 0th central moment (M0, Supplementary Note 4) and the lateral position from a Gaussian PSF model on incoherent PSFs that are the sum of the 4PiPSFs of the four channels (Fig. 1). Aquino's method refers to the algorithm developed in ref. [2] that extracts the axial positions using a combination of Gaussian-weighted 0th and 3rd central moments (M0+M3, Supplementary Note 4) and the lateral positions using a Gaussian-mask fitting algorithm on incoherent PSFs. Gaussian-mask fitting algorithm on lateral localization shows comparable performance within the designed axial range (-500 nm to 500 nm) of Aquino's method, but quickly deteriorates at out of focus region. Data were simulated from phase retrieved pupil functions without astigmatism modification and contain 1000 PSFs at each axial position in the range of -900 nm to 900 nm, with a step size of 100 nm. The total photon and background photon used were 1350 per objective and 1 per pixel. Please note that here we simulated the PSF data without astigmatism and with a photon count comparable with the bead data demonstrated in ref. [2] (Supplementary Table 1), in order to have a fair comparison with Aquino's method. And we applied the Gaussian mask fitting on incoherent PSFs rather than on 4PiPSFs of the four channels to improve localization precision and bias, because the complex features of 4PiPSFs cannot be modeled by a Gaussian function or an Airy disk. Here we used 'bias' for simulated data as the ground truth is known and 'deviation' for bead data. In all figures, without specification, PR-4Pi denotes the algorithm using 5 estimation parameters.

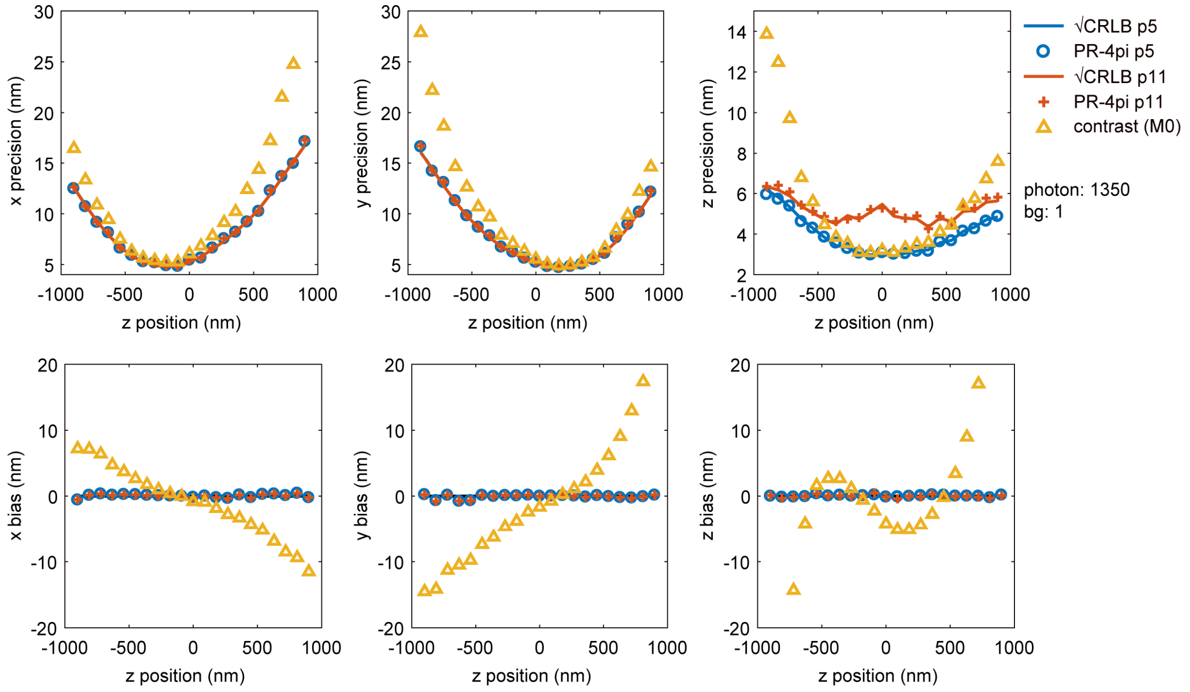

Supplementary Figure 7. Comparison of various localization methods with CRLB on simulated 4PiPSF data with astigmatism modification.

PR-4Pi algorithms with fixed (PR-4Pi p5, 5 parameters) and independent (PR-4Pi p11, 11 parameters) intensity and background between four quadrants were compared. Contrast method refers to the algorithm developed in ref. [1] that extracts the axial positions focusing on the Gaussian-weighted 0th central moment (M0, Supplementary Note 4). Data were simulated from phase retrieved pupil functions with enhanced astigmatism aberration and contain 1000 PSFs at each axial position in the range of -900 nm to 900 nm, with a step size of 100 nm. The total photon and background photon used were 1350 per objective and 1 per pixel.

Note that, for Fig. 2 and Supplementary Figs. 4-7, in order to generate localization results that are information limited without the artifact caused by ghost imaging, initial guesses that are close to global minimums were used for PR-4Pi algorithms. And for methods using Gaussian-weighted central moments, since the actual axial position increases monotonically with the frame number, the obtained phases can be easily unwrapped with MATLAB function *unwrap* and therefore eliminated ghost images, which however is not possible for cell data.

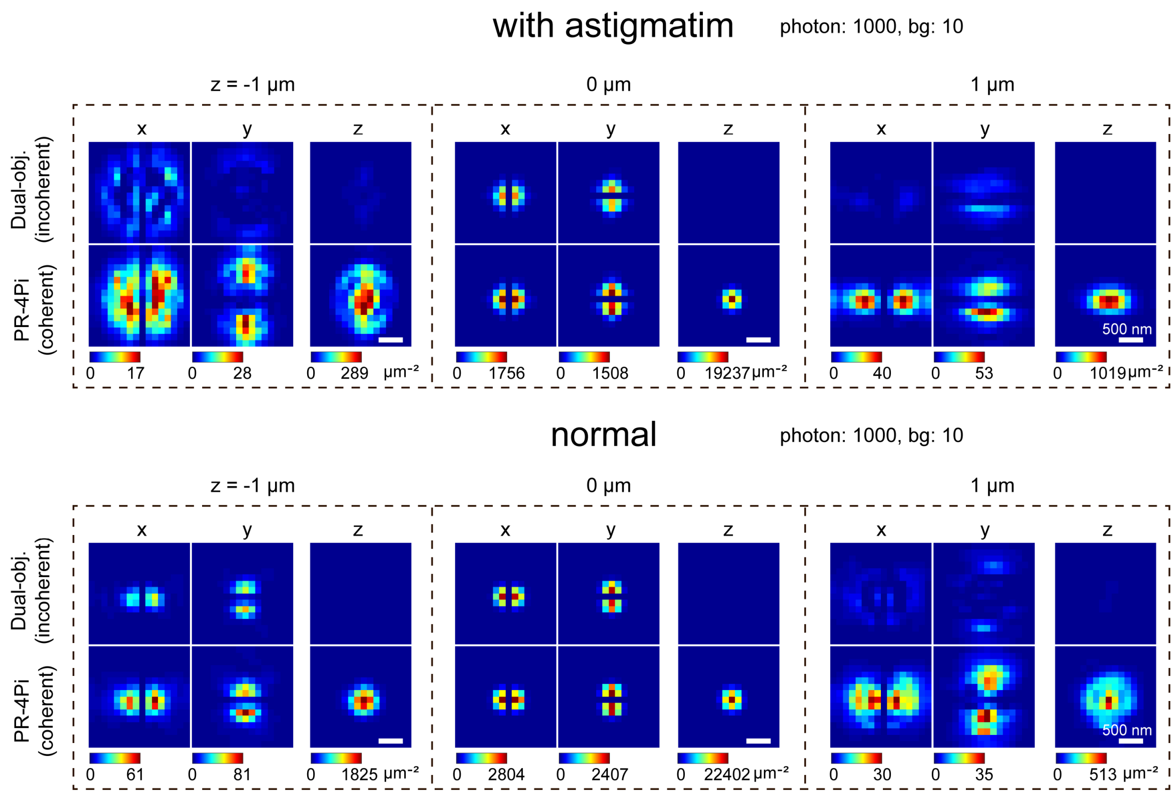

Supplementary Figure 8. Lateral distributions of the Fisher information content per pixel in x, y and z estimations based on PSF models with or without astigmatism modifications.
Same parameters were used for both PR-4Pi and dual-objective configurations, except that the dual-objective configuration uses incoherent detection (Supplementary Note 3). A total photon of 1000 per objective and a background (bg) photon of 10 per pixel were used for the PSF model generation.

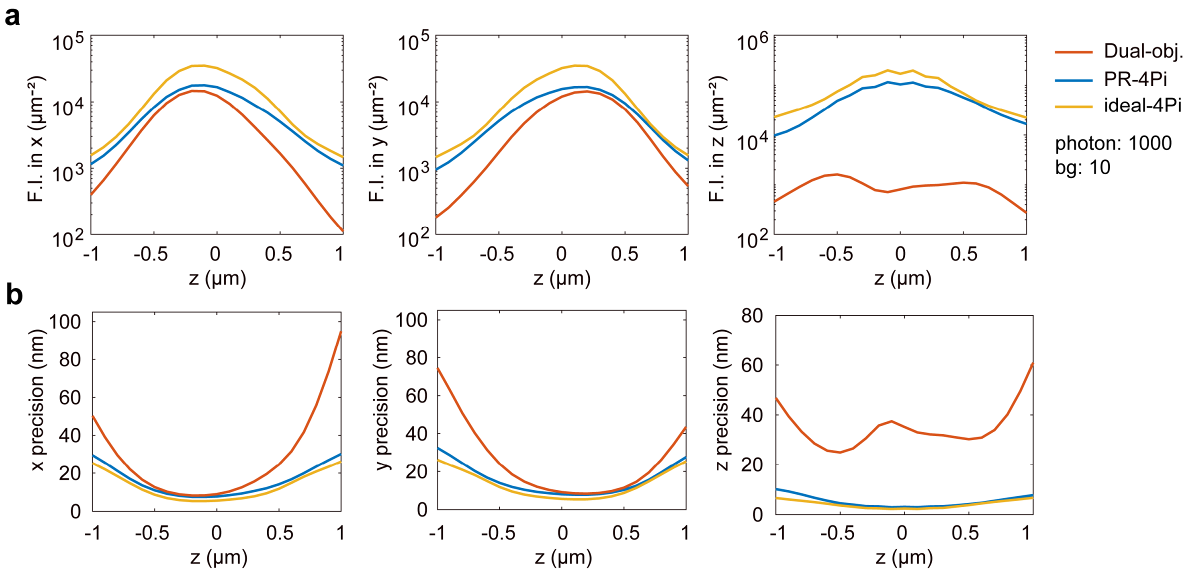

Supplementary Figure 9. Comparison of Fisher information and theoretical precisions from CRLB on various PSF models.

(**a**) Fisher information (F.I.) content in x, y and z estimations. (**b**) Theoretical localization precisions in x, y and z. A total photon of 1000 per objective and a background (bg) photon of 10 per pixel were used for the PSF model generation. Phase retrieved pupil functions were used for both PR-4PiPSF ($I_t = 0.7$, $\alpha = 0.7$, Eqs. 3 and 4 in Main Text) and dual-objective PSF. Ideal-4PiPSF models used unaberrated pupil functions and assumed no transmission loss and beam splitting inequality presented in the imaging system ($I_t = 1$, $\alpha = 1$).

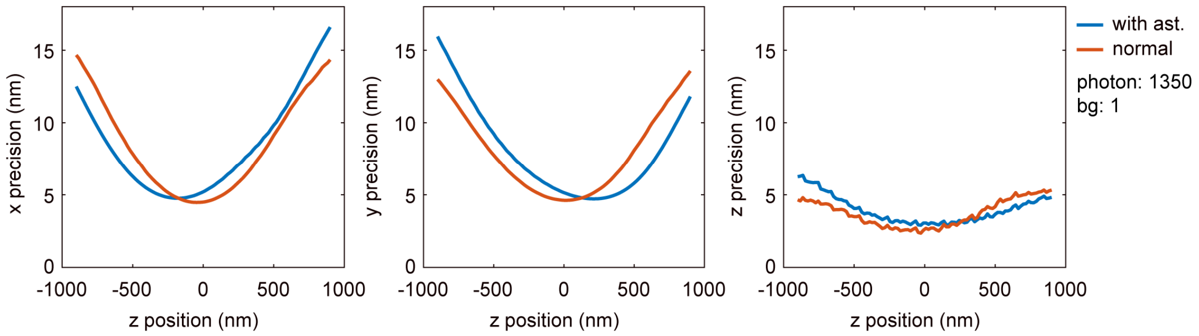

Supplementary Figure 10. Comparison of theoretical localization precisions from CRLB calculation of PR-4PiPSF models with and without astigmatism modifications.

It demonstrates comparable localization precisions between the two types of PR-4PiPSF models. PR-4PiPSF models were generated from phase retrieved pupil functions at axial positions from -900 nm to 900 nm with a step size of 100 nm. The total photon and background photon used were 1350 per objective and 1 per pixel.

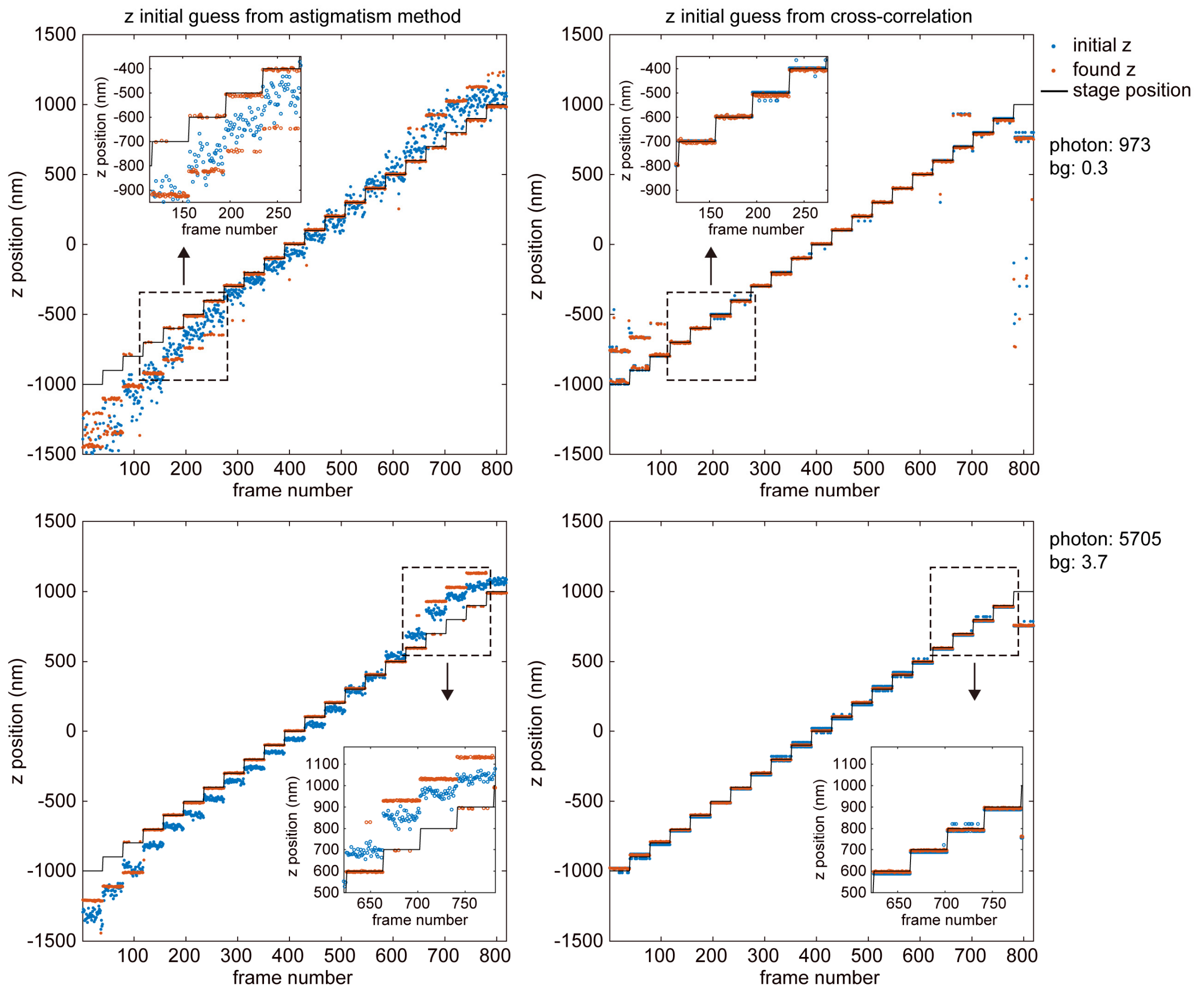

Supplementary Figure 11. Effect of initial guess on axial localization using PR-4Pi method. Initial guesses of axial positions of bead data from MLE fitting of incoherent PSFs with a Gaussian PSF model (astigmatism method) and from cross-correlations with a stack of PR-4PiPSFs (cross correlation method, Supplementary Note 4) were compared. Due to multiple local minimums in the likelihood function of 4PiPSFs (Supplementary Fig. 1), initial guess from astigmatism method results in more mislocalizations at out of focus region independent of photon count. Bead data were collected by imaging a 40 nm dark red bead at *z* positions from -1 μm to 1 μm, with a step size of 100 nm, 39 frames were captured at each axial position. photon: median total photon per objective (n=819), bg: median background photon per pixel (n=819). Photon and background were estimated from PR-4Pi method.

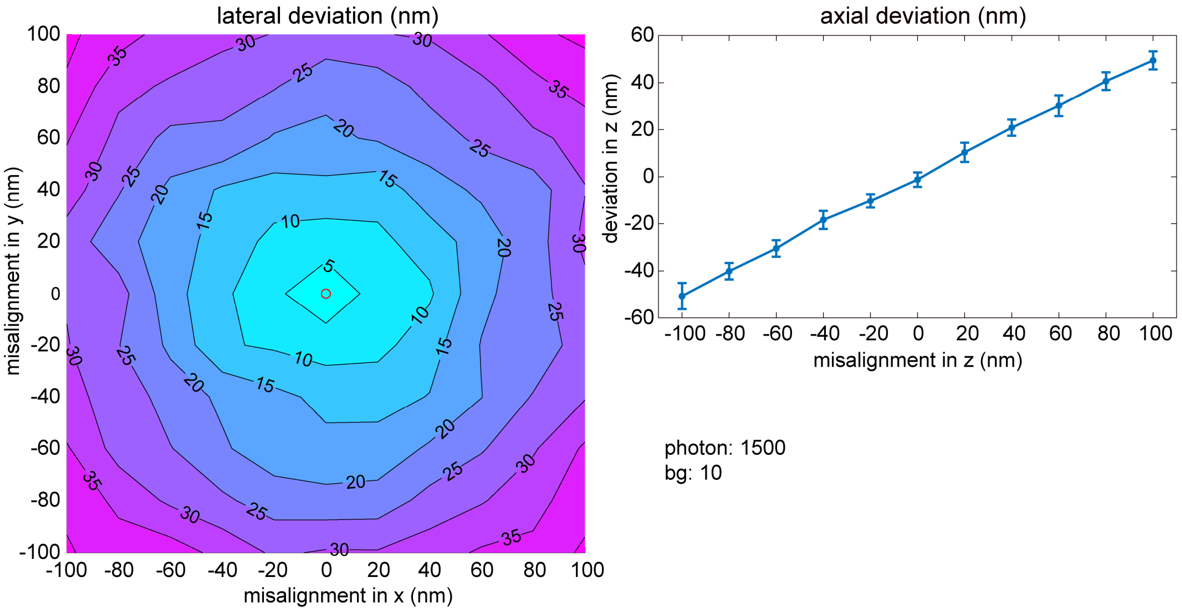

Supplementary Figure 12. Effect of the misalignment between the two objectives on localization deviations using PR-4Pi algorithm.

For each condition, 19 PSFs at *z* positions uniformly distributed from -600 nm to 600 nm were simulated with a total photon of 1500 per objective and a background of 10 per pixel. PR-4PiPSF models were generated based on the phase retrieved pupil functions by imaging a 40 nm bead. Contour plot: mean (n=19), error bar plot: mean±s.d. (n=19).

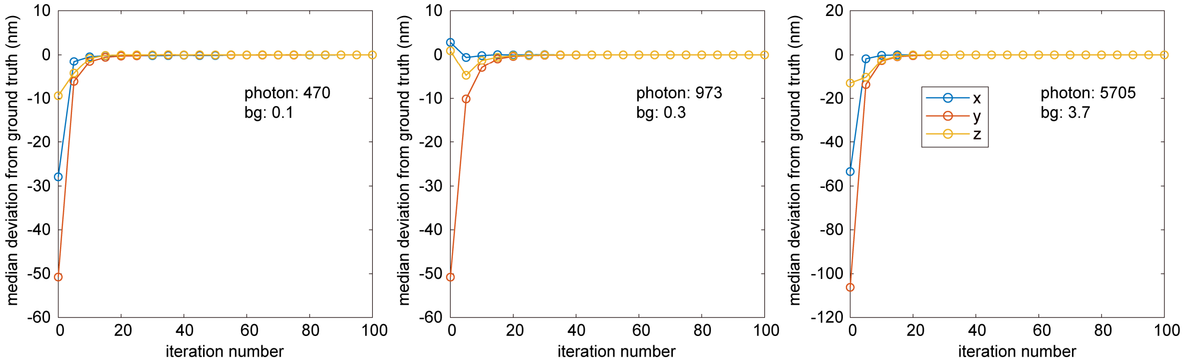

Supplementary Figure 13. Convergence of PR-4Pi algorithm on localization of experimental bead data with various photon count.

Bead data were collected by imaging a 40 nm bead at axial positions from -1 µm to 1 µm, 100 nm step size and 40 frames per step. The ground truth for x and y positions were calculated as the median of the localization results after 100 iterations and the ground truth for z positions were stage positions offset by a constant value to minimize the deviations after 100 iterations. photon: median total photon per objective (n=819), bg: median background photon per pixel (n=819).

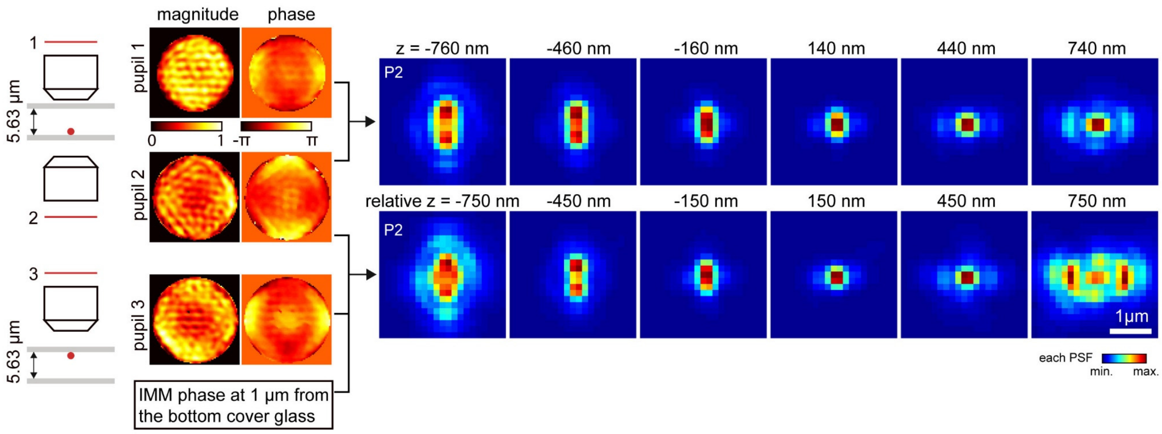

Supplementary Figure 14. PR-4PiPSF models considering index mismatch aberration (IMM). Two methods are presented here (Supplementary Note 6). Method one uses pupil 1 and pupil 2, retrieved independently through the upper and lower objectives by imaging a bead on the bottom cover glass. Pupil 1 includes the index mismatch aberration caused by imaging through the entire sample medium (e.g. 5.63 µm thick). Method two uses pupil 2 and pupil 3, where pupil 3 is retrieved by imaging a bead on the top cover glass through the upper objective. Method 1 is suitable for small imaging depth, less than 4 µm, while method 2 is designed to model emission patterns at arbitrary axial locations inside the sample medium given an imaging depth (e.g. 1 µm) measured from the bottom cover glass and the thickness of the sample medium.

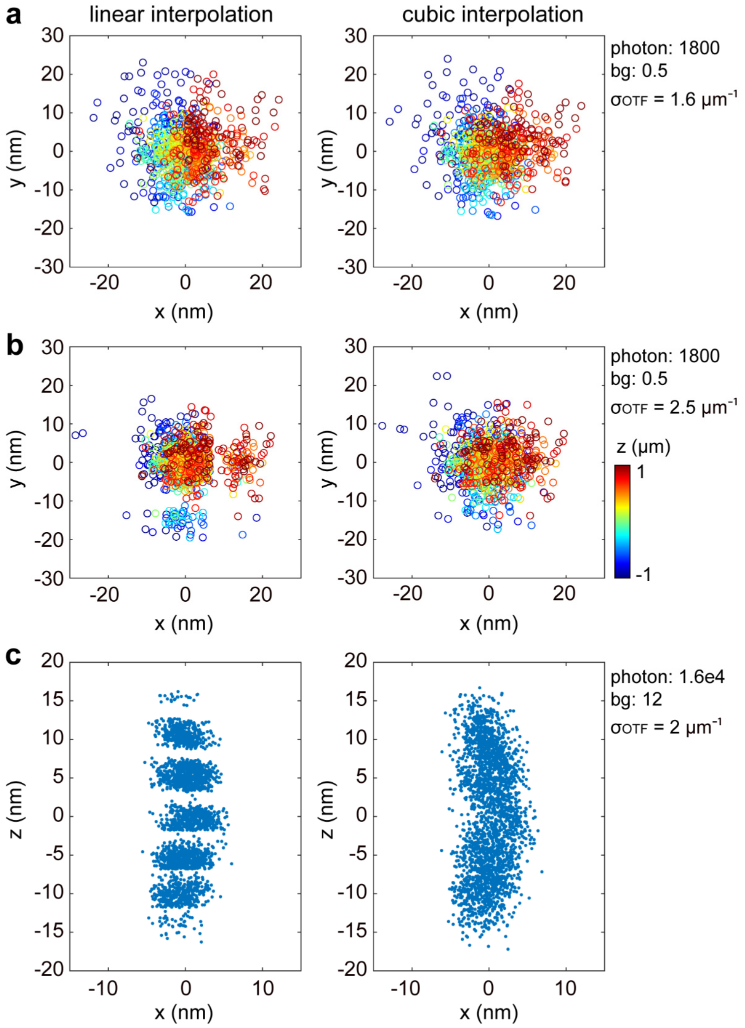

Supplementary Figure 15. Comparison of linear and cubic interpolation methods on localizations of bead data.

(**a, b**) Scatter plots of lateral localizations with linear and cubic interpolated PSF models, smoothed by an OTF rescaling factor (σ_{OTF} equals to $\sigma_{kx} = \sigma_{ky}$, Supplementary Note 1) of 1.6 μm^{-1} and 2.5 μm^{-1} respectively. Individual localization result is color coded according to its axial positions. (**c**) Scatter plots of axial localizations with linear and cubic interpolated PSF models, smoothed by an OTF rescaling factor of 2. We found linear interpolation tends to produce artifacts that are σ_{OTF} dependent in both lateral and axial localizations. Data for testing lateral localizations were acquired by imaging a fluorescence bead at axial positions from -1 to 1 μm by translating the sample stage with a step size of 100 nm, 40 frames were captured at each axial position. Data for testing axial localizations were acquired by imaging an in-focused fluorescence bead for 25 s at 10 Hz. No cavity-phase drift correction was applied for the localization results.

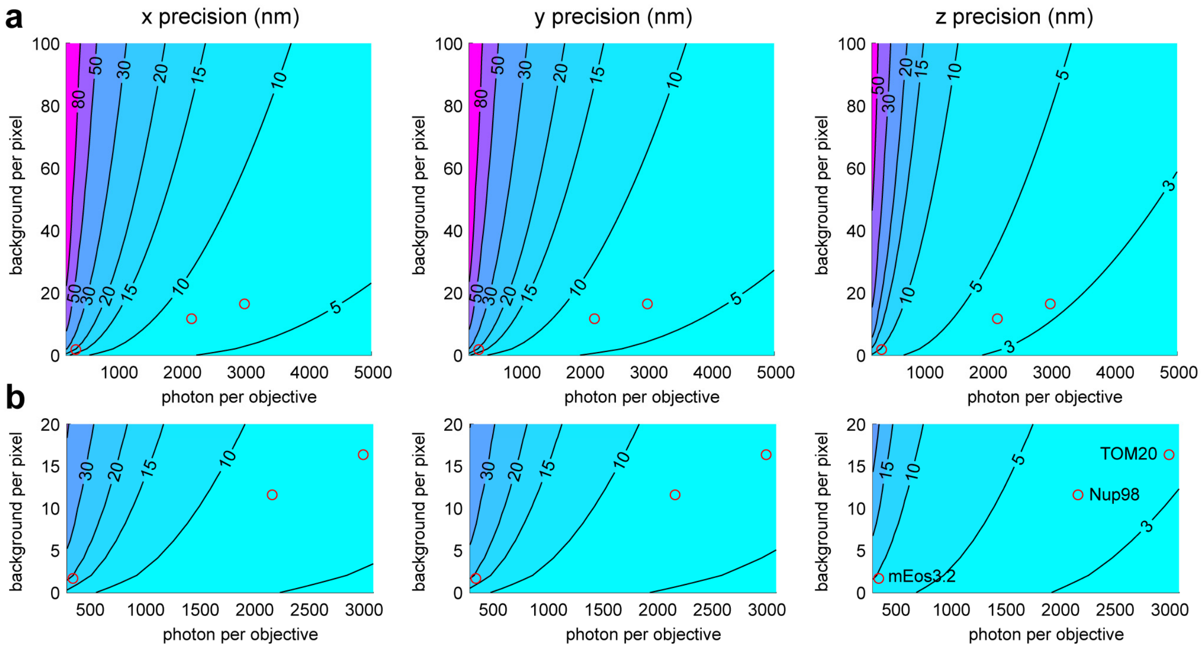

Supplementary Figure 16. Localization precision at different photon and background levels. Theoretical precisions based on CRLB were calculated from 4PiPSF models with various photon count per objective and background count per pixel (Main Text). The precision value under each condition was the average of 19 calculations at *z* positions uniformly distributed from -600 nm to 600 nm. Red circles indicate the achievable precisions given the medians of the estimated photon and background counts of the localized emission events from various demonstrated biological specimens (Figs. 3,4 and Supplementary Fig. 24).

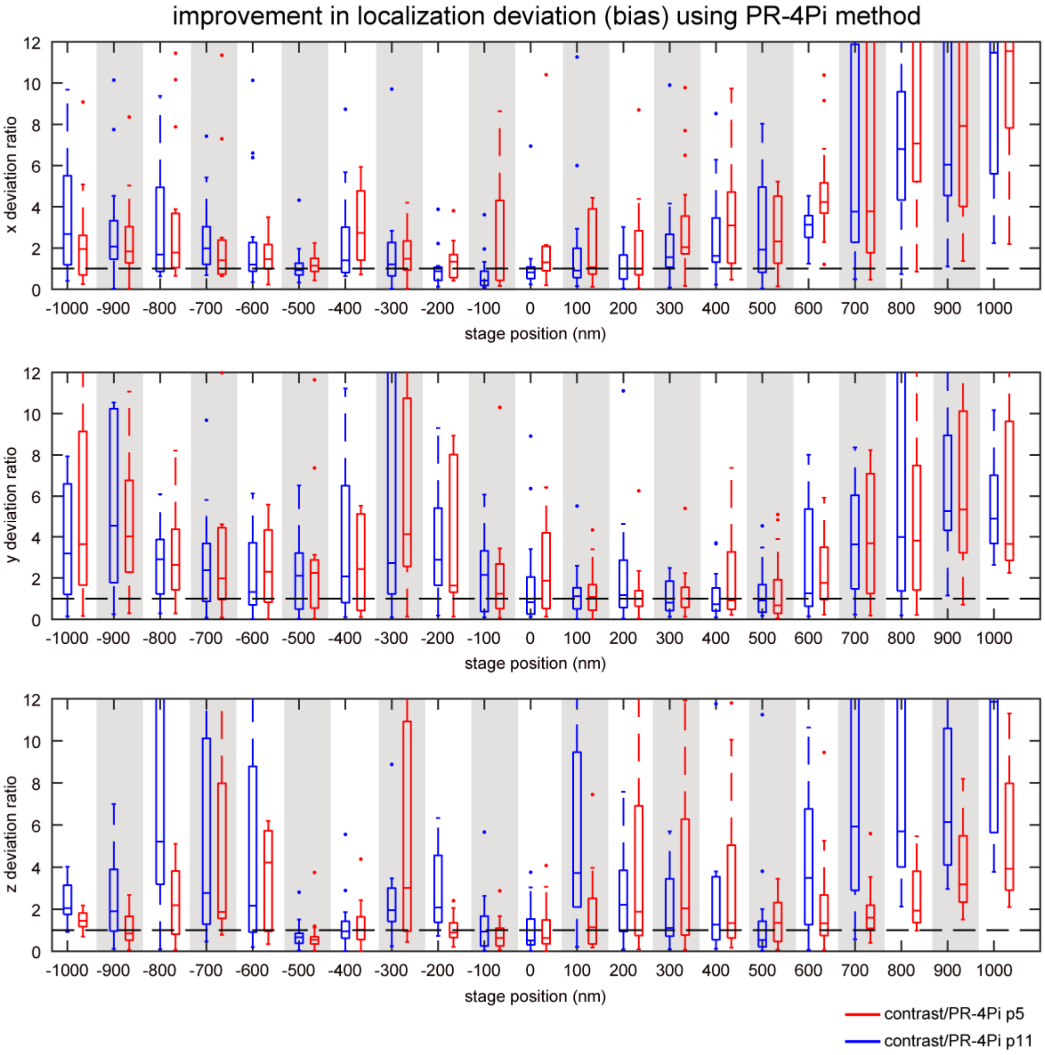

Supplementary Figure 17. Ratios of absolute localization deviations (bias) between contrast and PR-4Pi methods on experimental bead data.

PR-4Pi with fixed (p5, 5 parameters, red) and independent (p11, 11 parameters, blue) intensity/background between four quadrants were compared. At each axial position, one box plot was generated from absolute deviation ratios of localizing 17 bead data (median photon of 470 to 5705 (515 to 7091) per objective reported from PR-4Pi p5 (PR-4Pi p11)) using contrast method and PR-4Pi p11 (or PR-4Pi p5) method, using the MATLAB function *boxplot*. On each box, the central mark indicates the median, and the bottom and top edges of the box indicate the 25th and 75th percentiles, respectively. The whiskers extend to the most extreme data points not considered outliers. The outliers (blue and red points) were defined as the data points lie outside $[q_1 - 1.5w, q_2 + 1.5w]$, where q_1 and q_2 are the values at 25th and 75th percentiles respectively and w is the length of the box. The ratio plots show improved localization bias by using PR-4Pi methods comparing with contrast method, especially at out of focus region. And in the axial dimension, PR-4Pi p11 out performs PR-4Pi p5 at out of focus region. Bead data for localization were analyzed using the same PR-4PiPSF models which were generated from phase retrieved pupil functions. Bead data for phase retrieval and localizations were collected from different beads. All bead data were collected during the same experiment and from the same bead sample.

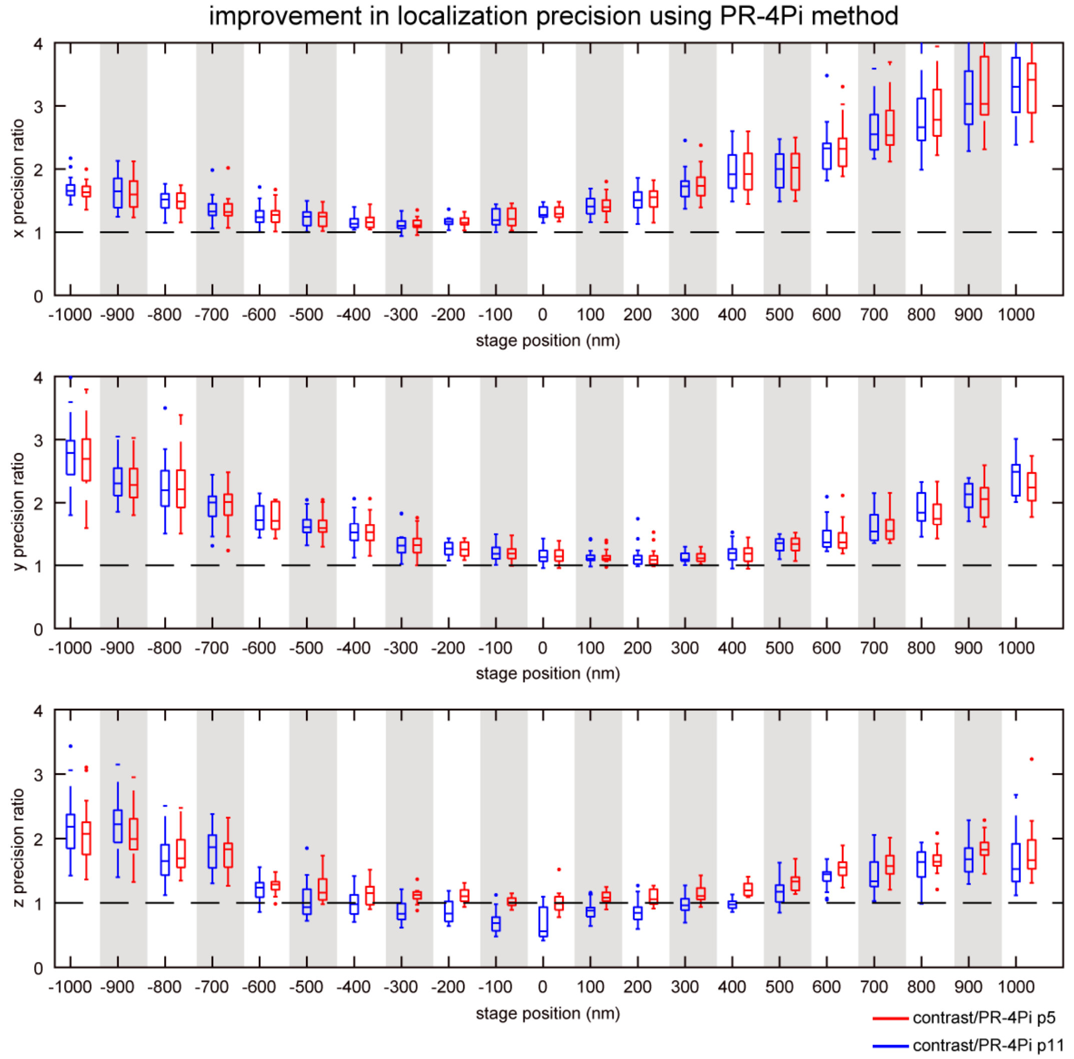

Supplementary Figure 18. Ratios of localization precision between contrast and PR-4Pi methods on experimental bead data.

PR-4Pi with fixed (p5, 5 parameters, red) and independent (p11, 11 parameters, blue) intensity/background between four detection channels were compared. At each axial position, one box plot was generated from precision ratios of localizing 17 bead data (median photon of 470 to 5705 (515 to 7091) per objective reported from PR-4Pi p5 (PR-4Pi p11)) using contrast method and PR-4Pi p11 (or PR-4Pi p5) method, using the MATLAB function *boxplot*. See Supplementary Fig. 17 for the definition of each box. The ratio plots show improved localization precisions by using PR-4Pi methods comparing with contrast method at out of focus region. And in the axial dimension, PR-4Pi p11 shows worsened precision than contrast method at near focus region. Bead data for localization were analyzed using the same PR-4PiPSF models which were generated from phase retrieved pupil functions. Bead data for phase retrieval and localizations were collected from different beads. All bead data were collected during the same experiment and from the same bead sample

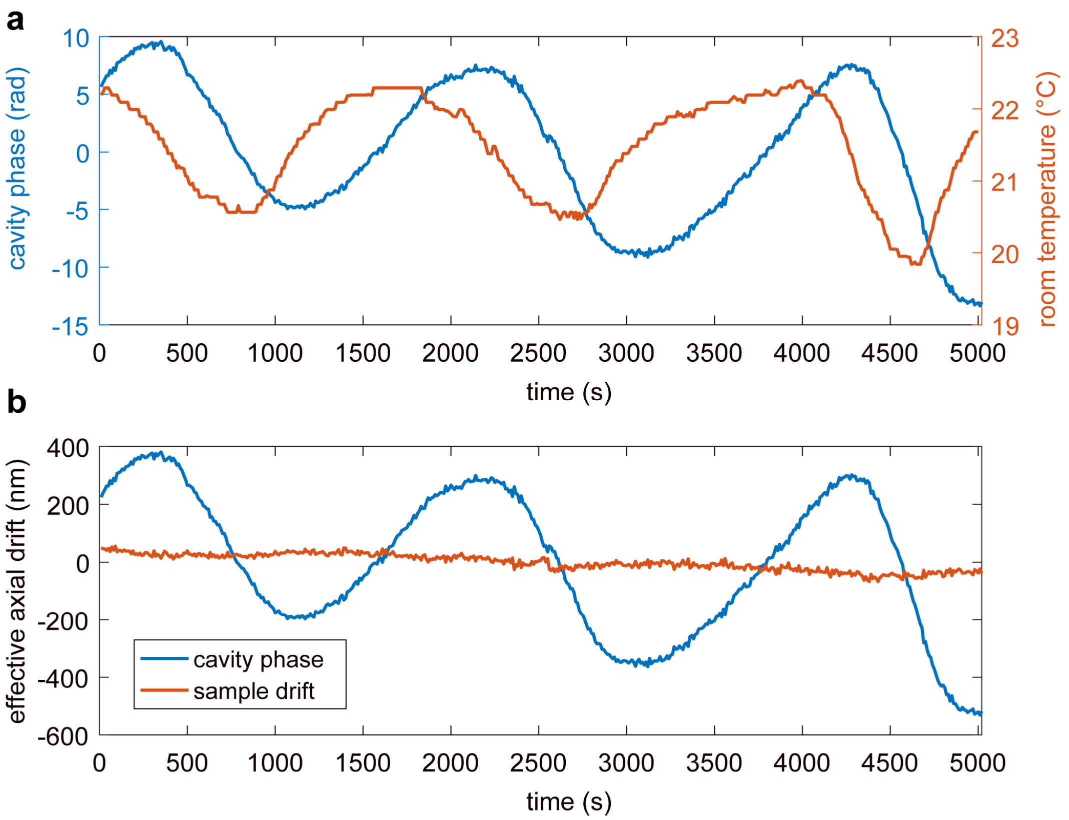

Supplementary Figure 19. Correlation of temperature fluctuation and cavity phase drift.
The room temperature was measured using a temperature sensor (TSP01, Thorlabs, resolution at 0.05 °C) at a sequence of 500 time points with 10 s intervals, and at the same time the cavity phase drift was measured by imaging a fluorescence bead (Supplementary Note 7). (**a**) Fluctuations of cavity phase and room temperature show strong correlations. (**b**) The effective axial-drift measurements from cavity phase drift and the sample drift. The cavity-phase induced axial-drift is the main factor that deteriorates the axial resolution of 4Pi-SMSN systems.

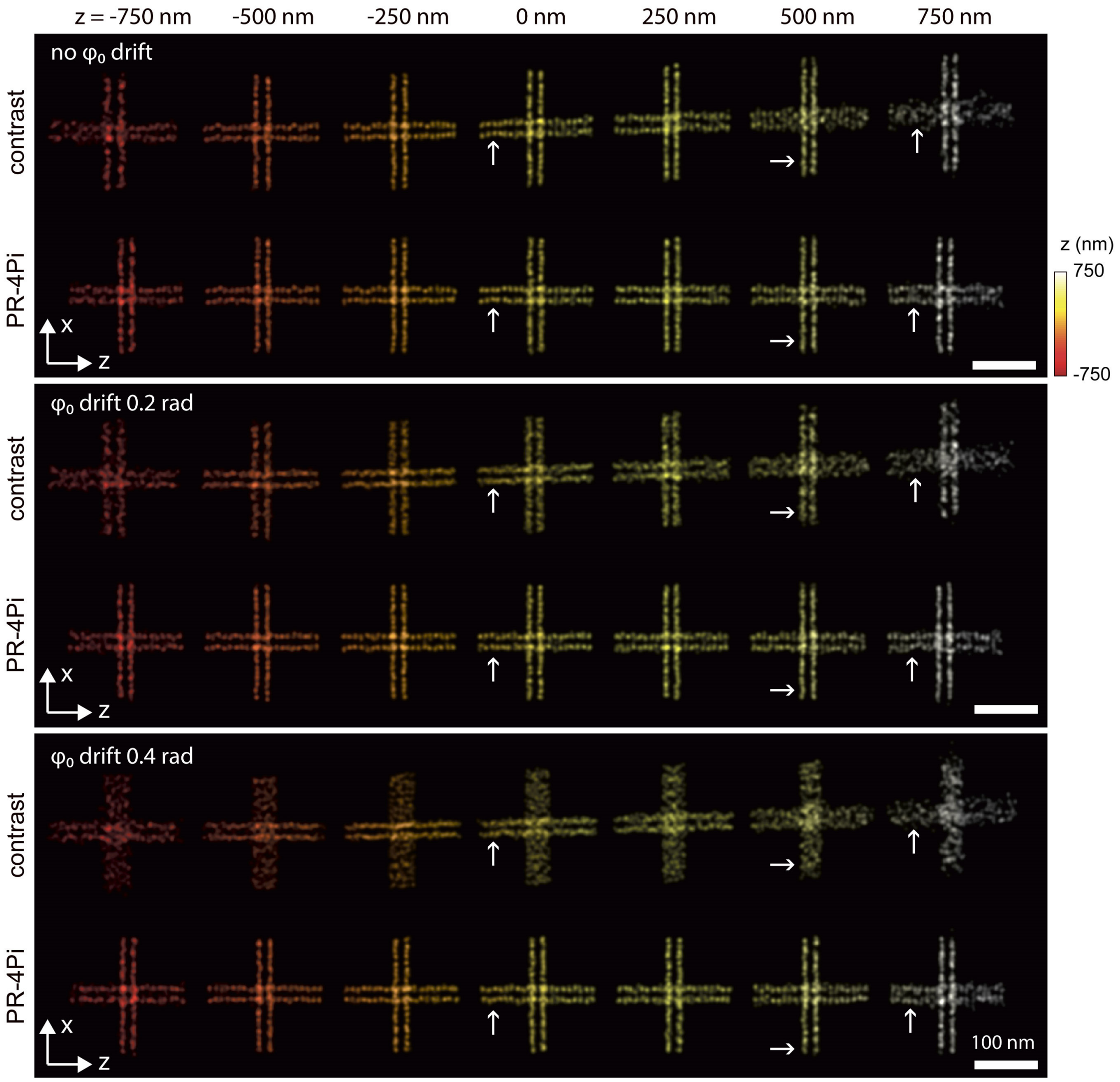

Supplementary Figure 20. Effect of cavity phase drift on axial resolution.

A double-line cross structure at the x-z plane was simulated at various axial positions. Each line measures 200 nm in length and contains 20 points spaced uniformly, and the double lines are separated by 20 nm. Five PSFs with cavity phase (φ_0) equally spaced between 0 up to 0.4 rad were generated at each point of the simulated structures using phase retrieved pupil functions. We found that in most cases, PR-4Pi succeeded in precise localization of these 20 emitters per line resulting in almost equally spaced and distinct dots along the structure. In comparison, contrast based method manages to resolve these ultra-structures when it is close to the common focus with 0 cavity phase drift (first row, *z* at 0 nm), but fails to resolve these ultra-structures in most cases. In lateral dimensions, the contrast based method results in larger deviations and worse resolutions at out-of-focus positions. In the axial dimension, the contrast based method fails to resolve the double lines under a φ_0 drift of 0.4 rad, which is below the average cavity phase drift (0.45 rad, measured from our system) within 40 s, the shortest time segment for post drift correction. Data were simulated from phase retrieved pupil functions, and the total photon and background photon used were 5000 per objective and 10 per pixel.

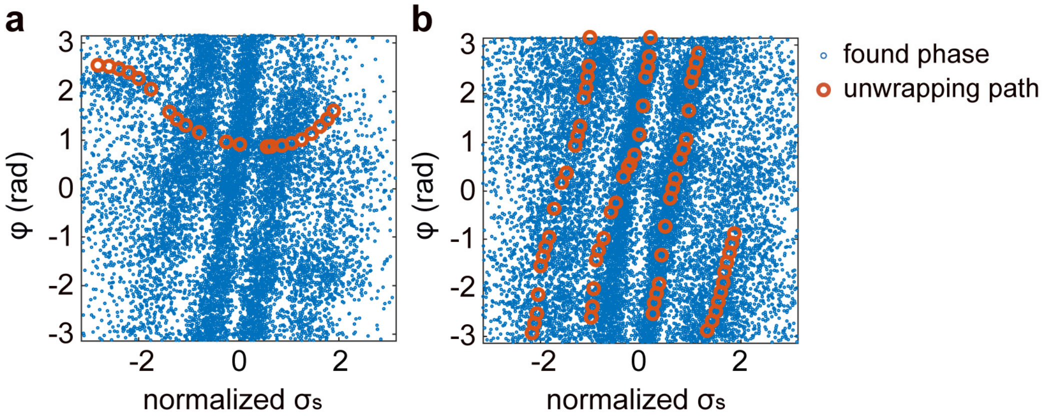

Supplementary Figure 21. Limitations of phase unwrapping method (ridge-finding algorithm) in contrast algorithm.

(**a, b**) Scatter plots of phase vs. sigma metric of found emitters in 2000 frames from Nup98 imaging in COS-7 cells, which shows the unwrapping path (red circle) is either completely off or deviates from the stripe patterns (blue dots). Such failures occur occasionally within the whole dataset of Nup98 and TOM20 imaging.

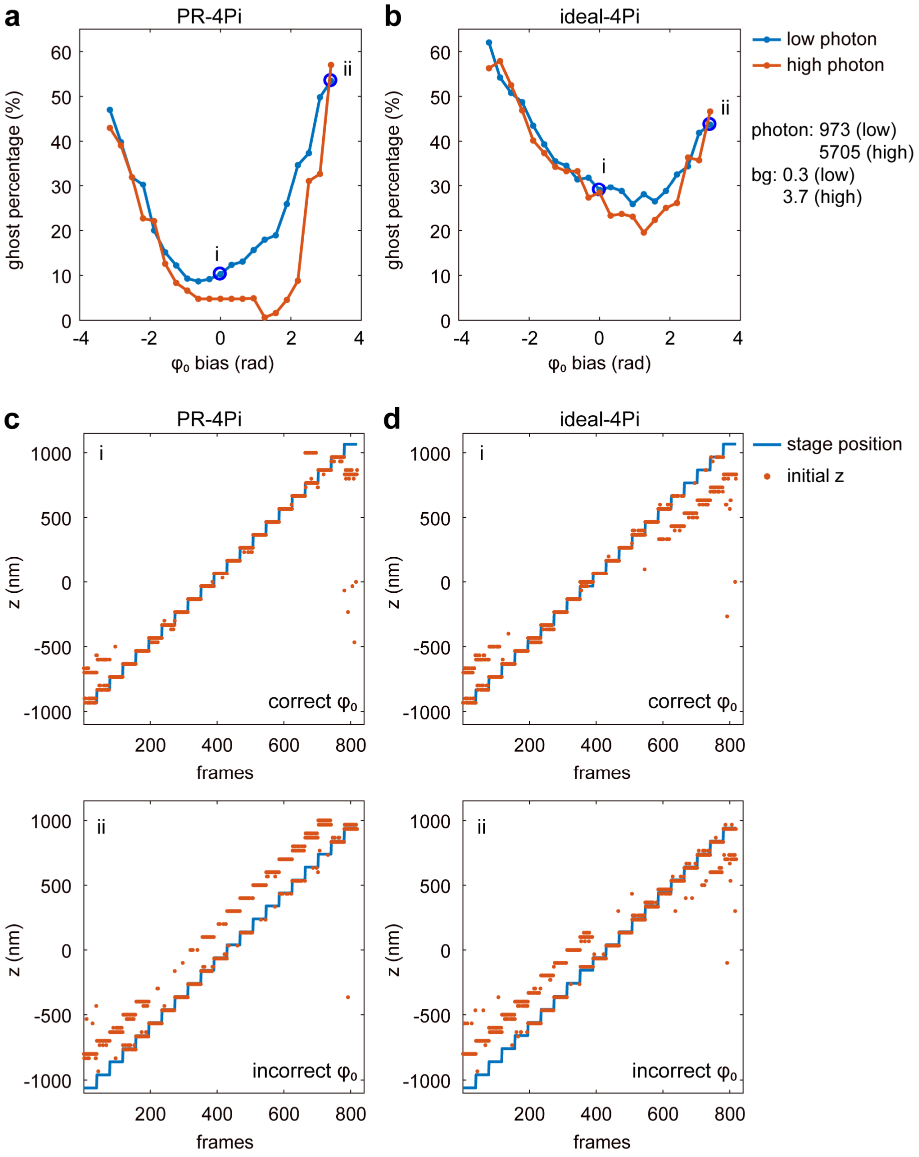

Supplementary Figure 22. Effect of cavity phase bias on the percentage of ghost localizations. The PR-4PiPSF models were generated from phase retrieved pupil functions and the ideal-4PiPSF models used unaberrated pupil functions and assumed no transmission loss and beam splitting inequality presented in the imaging system. (**a, b**) Ghost percentage from PR- and ideal-4PiPSF models with different cavity phase (φ_0) biases. (**c, d**) z initial guessed positions of the bead data using the PR- and ideal-4PiPSF models at small and large cavity phase biases (blue circles in a and b). The ghost percentage is defined as the percentage of the initial z positions (red dots) at a distance greater than 200 nm from the corresponding stage positions (blue lines). Data were acquired by imaging a dark red bead at axial positions from -1 to 1 µm by translating the sample stage with a step size of 100 nm, 39 frames were captured at each axial position (Methods). The estimation results from PR-4Pi method yielded a mean total photon of 973 (5705) (n=819) per objective and a background (bg) photon of 0.3 (3.7) (n=819) per pixel for bead data with low (high) signal to noise ratio (SNR).

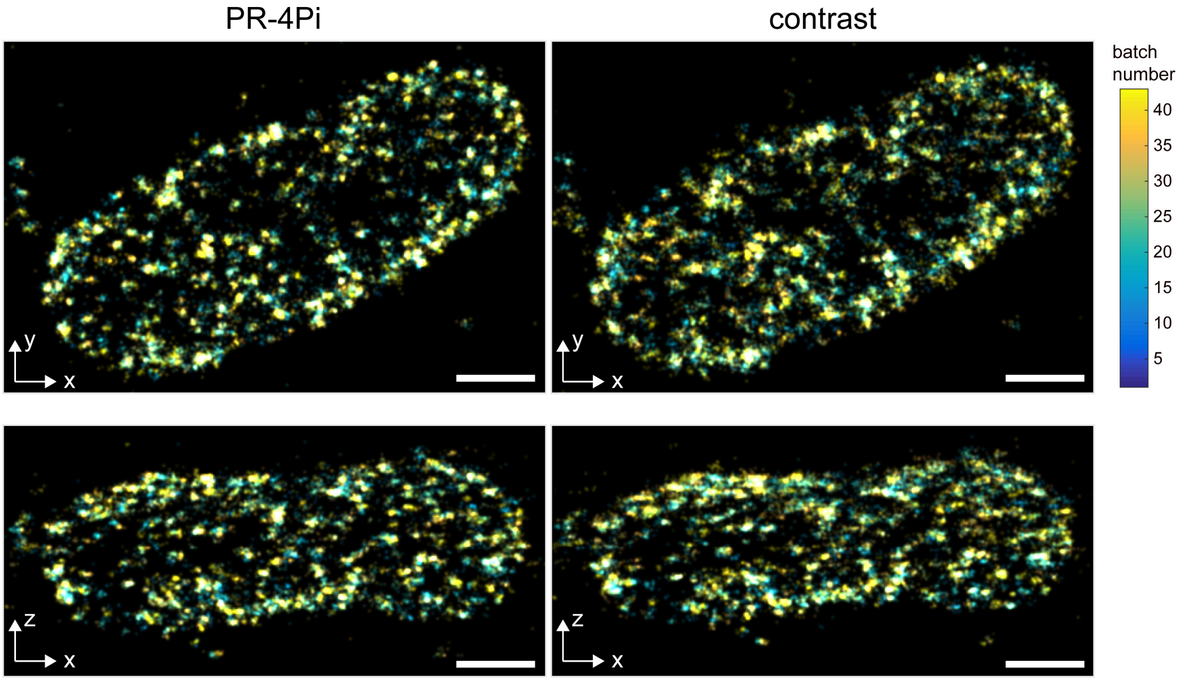

Supplementary Figure 23. Localization results after drift correction with color scaled in time (batch number).
The selected mitochondria (white box) in Fig. 4a was used as a demonstration. The x, y and z shifts between each batch were estimated using a redundancy-based drift correction method (Supplementary Note 4) and the number of frames in each batch is 2000 (Fig. 3) and 4000 (Fig. 4 and Supplementary Fig. 24). Scale bar: 200 nm.

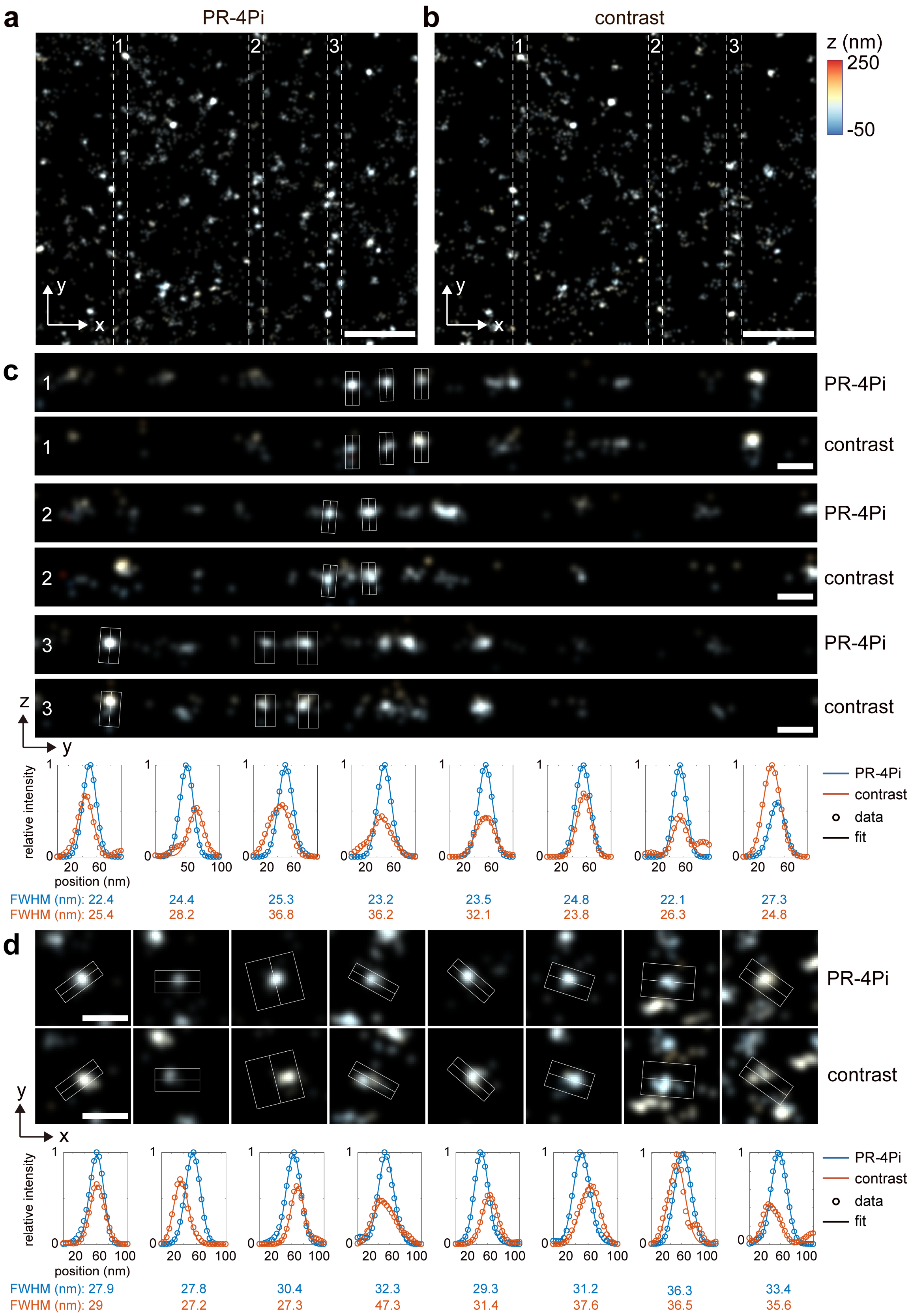

a
PR-4Pi
b
contrast
z (nm)
250
-50
1
2
3
y
x
c
PR-4Pi
contrast
z
y
relative intensity
position (nm)
PR-4Pi
contrast
data
fit
FWHM (nm): 22.4 24.4 25.3 23.2 23.5 24.8 22.1 27.3
FWHM (nm): 25.4 28.2 36.8 36.2 32.1 23.8 26.3 24.8
d
PR-4Pi
contrast
y
x
relative intensity
position (nm)
FWHM (nm): 27.9 27.8 30.4 32.3 29.3 31.2 36.3 33.4
FWHM (nm): 29 27.2 27.3 47.3 31.4 37.6 36.5 35.6

Supplementary Figure 24. SMSN reconstruction of mEos3.2 on coverslip.
(**a, b**) Lateral view of the SMSN reconstruction with PR-4Pi and contrast methods. (**c**) Axial cross sections (y-z plane) of the dash-line regions in (a) and (b) and profiles of selected single clusters in the y-z plane. (**d**) Selected single clusters in the x-y plane and their profiles. Each profile was measured along the central line by averaging the pixel values within the white box along the orthogonal dimension of the central line. Each profile was fitted by a 1D Gaussian with a width of σ, and the FWHM was calculated from 2.355σ. Scale bars: 500 nm in (a,b), 100 nm in (c,d).

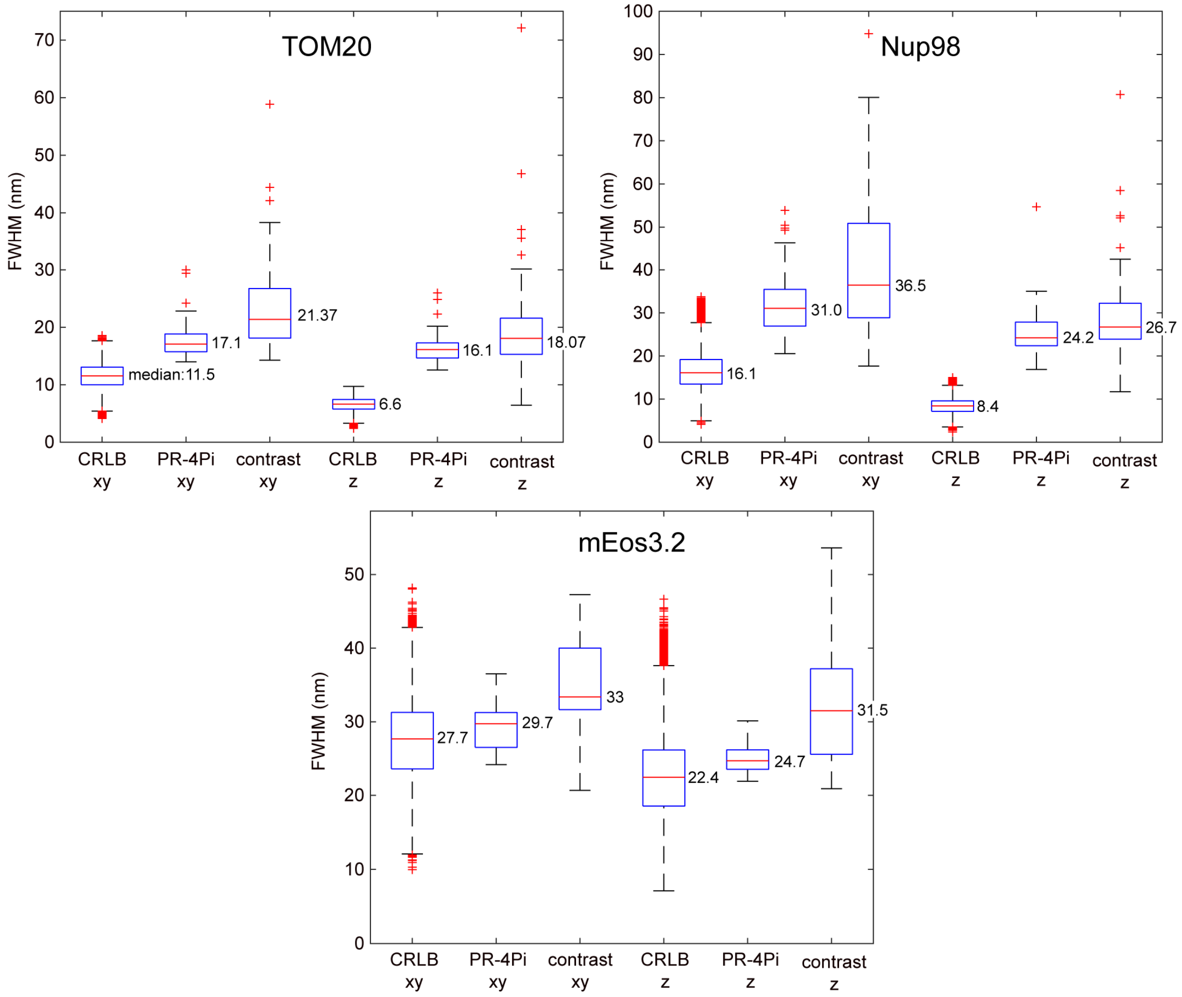

Supplementary Figure 25. Cluster profile quantifications of PR-4Pi and contrast methods on various biological specimens.

Cluster profiles of resolved TOM20 and mEos3.2 were quantified by measuring the width of selected single clusters in x-y and y-z planes. Profiles of Nup98 was quantified by measuring the edge width of selected nucleus pore complexes. The width was defined as the FWHM of the profile fitted with a 1D Gaussian (Figs. 3,4 and Supplementary Fig. 24). The FWHM from CRLB was defined as $2.355\sigma_{CRLB}$, where σ_{CRLB} is the precision based on CRLB given the estimated photon and background of one emission event. For FWHM of cluster profiles, each box summarizes 30-70 profile measurements (TOM20: 58~66, Nup98: 44~66, mEos3.2: 28~40) using the MATLAB function *boxplot*. For FWHM from CRLB, each box was generated from all accepted localization results (TOM20: 1.3×10^6, Nup98: 1.8×10^5, mEos3.2: 8.8×10^4). On each box, the central mark indicates the median, and the bottom and top edges of the box indicate the 25th and 75th percentiles, respectively. The whiskers extend to the most extreme data points not considered outliers. The outliers (red crosses) were defined as the data points lie outside $[q_1 - 1.5w, q_2 + 1.5w]$, where q_1 and q_2 are the values at 25th and 75th percentiles respectively and w is the length of the box.

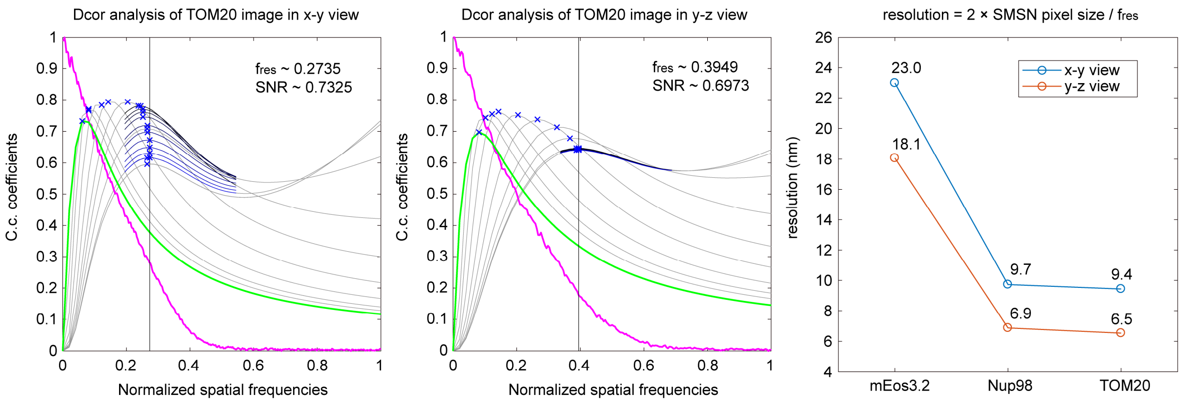

Supplementary Figure 26. Resolution quantification of PR-4Pi on various biological specimens using decorrelation analysis.

SMSN reconstructions in x-y and y-z views were used as the input images for decorrelation (Dcor) analysis [3]. Result plots from Dcor analysis on TOM20 reconstructions in x-y and y-z views are shown. Sub-regions (region ii in Fig. 3a and a selected sub-region (white-box) in Fig. 4a) were used for Dcor analysis on Nup98 and TOM20 reconstructions. SMSN reconstructions were generated at a pixel size of 1.3~2.6 nm, and each localization was blurred by a normalized 2D Gaussian with its standard deviation at each dimension equal to the theoretical estimation precision (based on CRLB). Please note that we only used decorrelation analysis on reconstructed images from PR-4Pi localization results, because contrast method doesn't output CRLB information for each localized emitter. Therefore, in other demonstrations, to achieve a fair comparison between PR-4Pi and contrast methods, we generated each SMSN image using a fixed Gaussian blur for all localizations (Supplementary Note 4). For the plots of decorrelation analysis: green line, decorrelation functions before high-pass filtering; magenta line, radial average of log of absolute value of Fourier transform of the input image; gray lines, all high-pass filtered decorrelation functions; blue to black lines, decorrelation functions with refined mask radius and high-pass filtering range. Blue crosses, all local maxima. Dashed vertical line, cut-off frequency (f_{res}).

Supplementary Tables

	iPALM [4]	iPALM+Ast [5]	4Pi-SMSN [2,6]	W-4PiSMSN [1] (contrast method)	PR-4Pi
reference	Shtengel, *PNAS*, 2009	Brown, *Mol.Cell.Biol.*,2011	Aquino, *Nat.Methods*, 2011	Huang, *Cell*, 2016	this work
z precision /resolution[1] (cell)	15-30 nm (resolution, FWHM)	N.A.	~5-7 nm (precision)	8-22 nm (resolution, FSC)	~16-25 nm† (resolution, FWHM)
xy precision /resolution[1] (cell)	N.A.	N.A.	~10-14 nm (precision)		~17-31 nm† (resolution, FWHM)
axial range	225 nm	~750 nm	~650 nm, 1 µm for bead	~10 µm, ~1.2 µm for single section (bead)	~3 µm, 1.8 µm for single section (bead)
ghost percentage (bead)	N.A.	N.A.	~0-12%, 2% on average (1 µm z range)	N.A.	0~5%, 1.2% on average (1.8 µm z range)
ghost percentage (cell)	N.A.	N.A.	N.A.	N.A.	~2%
z fitting model	intensity contrast	intensity contrast +astigmatism	M0+M3 intensity contrast[2]	M0 intensity contrast[2] +astigmatism	PR-4PiPSF
xy fitting model	Airy function	2D Gaussian	Gaussian mask fitting	2D Gaussian	PR-4PiPSF
structure type	<225 nm thick sample	500 nm thick sample	~650 nm thick sample	~10 µm thick sample with continuous z distribution	~3 µm thick sample
cavity phase correction	N.A.	N.A.	N.A.	corrected per 3000-5000 frames	corrected per 500 frames
objective stabilization	N.A.	N.A.	Integrated with a stabilization module [6]	real-time stabilization	N.A.[3]
Sample drift correction	fiducial marker	N.A.	post-drift correction per 10,000 frames	post-drift correction per 3000-5000 frames	post-drift correction per 2000-4000 frames

Supplementary Table 1. Comparison of interferometric single-molecule localization microscopies
[1]Precision is calculated from the standard deviation of repeated measurements of the same position. FWHM is a measure of resolution and equivalent to 2.355×precison.
[2]M0 and M3 represent the Gaussian-weighted 0th and 3rd central moment of the PSF pattern [2] (Supplementary Note 4).
[3]For the presented data in this work, we didn't observe large misalignment of the two objectives during the entire data acquisition process. A potential compensation of objective misalignment can be achieved by real time measurement of misalignments in x, y and z, and integrate the measurements in 4PiPSF model, which can minimize the error and vibration caused by the feedback system. Supplementary Fig. 12 demonstrates the effect of objective misalignment on localization deviations.
†The median of the FWHM of selected single clusters from the localization results of cell (protein) data (Supplementary Fig. 25).

Procedures of PR-4Pi algorithm	Run time for 500 frames		Percentage[2] of run time	Device
Cavity phase calibration	10.2 s		3%	CPU
Segmentation	27.6 s		8.1%	CPU
Initial guess (x,y,I,bg) and astigmatism fitting[1]	23.7 s	431 fits/s	6.9%	CPU
PR-4PiPSF library generation	21.4 s	104 fits/s	6.3%	CPU
Initial z guess	101.9 s		29.8%	CPU
1st MLE localization	41.1 s	258 fits/s	12%	GPU
find ghost	16 s		4.7%	CPU
2nd MLE localization	41.1 s	258 fits/s	12%	GPU
Mis-localization removal	3.6 s	2885 fits/s	1%	CPU
3D drift correction	55.5 s		16.2%	CPU
Total time	342.1 s		100%	

Supplementary Table 2. Run time of PR-4Pi algorithm.
[1]Astigmatism fitting results are used for ghost reduction (Supplementary Note 4)
[2]Time percentage of each procedure depends on the number of fits within the 500 frames and varies between different data segments and datasets. The run time and percentage here were obtained from the first 500 frames of the Nup98 dataset, containing 10598 fitting subregions (Fig. 3).
The most time consuming procedures (highlight in red) are initial z guess, MLE localization and drift correction (>10%).

Supplementary Notes

1. Estimations of hyper parameters in PR-4PiPSF model

In PR-4PiPSF model, besides the cavity phase φ_0 and the Zernike coefficients (generated from the phase retrieval process), which are used to form the shape of the pupil function, there are a few hyper parameters are also crucial to build an accurate 4PiPSF model. Those hyper parameters are constants during the localization process and include: the refractive indices of the immersion medium n_{imm} and the sample medium n_{med}, the emission wavelength λ, the quartz induced phase difference between s- and p-polarizations $\Delta\varphi_{\text{sp}}$, the OTF rescaling factors σ_{kx} and σ_{ky}, the intensity ratio between the two emission paths I_{t} and the coherence strength α.

The refractive indices were measured from an Abbe refractometer (334610, Thermo Spectronic), which are $n_{\text{imm}} = 1.516$ for the immersion oil (16241, Cargille) and $n_{\text{med}} = 1.351$ for the SMSN imaging buffer (Methods).

The emission wavelength was determined by imaging a dark red bead (40 nm, F8789, Invitrogen) for Alexa Fluor 647(A21245, Life Technologies) tagged sample or a red bead (40 nm, F8793, Invitrogen) for mEos3.2 sample, at z positions from -1 to 1 μm with a step size of 100 nm and taking 40 frames per z position. An optimum λ (675 nm for dark red bead and 595 nm for red bead), was found by minimizing the error of the estimated step size and the stage step size, which is 100 nm. Note that this wavelength is determined by moving the sample stage, which effectively changes the thickness of the immersion medium. However, for SMSN imaging, the change of the axial position is relative to the sample medium, therefore, to account for the index mismatch, a longer wavelength of $\lambda n_{\text{imm}}/n_{\text{med}}$ was used (Supplementary Note 6).

The phase difference $\Delta\varphi_{\text{sp}} = \varphi_{\text{s}} - \varphi_{\text{p}}$ was estimated from a stack of bead images at z positions from -1 to 1 μm with a step size of 10 nm. We first obtained the intensity modulations, r_{s} and r_{p}, which modulate at different phases (Supplementary Note 4) from the bead data. We then fitted the two modulation curves with cosine functions and calculated the phase difference from the fitting results as $\Delta\varphi_{\text{sp}}$, typically around -1.5 rad.

The OTF rescaling is to account for the PSF broadening effect from isotropic dipole emission of a single fluorophore [7,8]. It performs as a Gaussian blur and the factors, σ_{kx} and σ_{ky} (unit: μm^{-1}), are the widths of the blurring kernel in the Fourier space, which is equivalent to a Gaussian blurring kernel in real space with widths of $1/2\pi\sigma_{kx}$ and $1/2\pi\sigma_{ky}$ (unit: μm). They were estimated by minimizing the log-likelihood

ratio between simulated and measured PSFs, which contains PSF images at z positions from -1 to 1 μm with a step size of 100 nm. Here we used 1.6-2 μm^{-1} for both σ_{kx} and σ_{ky}. The intensity ratio (I_t) was selected by minimizing the deviations of σ_s values obtained from PR-4PiPSFs (from localization of measured PSFs) and measured PSFs for each experiment. We found that an incorrect I_t will change the slope of σ_s with respect to the axial positions (Supplementary Fig. 1). The coherence strength a was empirically set to 0.7 under which PR-4Pi achieves better convergence at near focus region. Further discussions on intensity ratio and coherence strength were detailed in Supplementary Note 2.

2. Effect of intensity ratio and coherence strength

As described in the PR-4PiPSF generation (Main Text), both the intensity ratio I_t and the coherence strength a can affect the modulation depth of the 4PiPSF and consequently influence the localization precision. We investigated the theoretical localization precision of 4PiPSFs with various coherence strength and intensity ratios based on CRLB (with 1500 photon per objective and 10 background photon per pixel, Supplementary Fig. 2): 1) When intensity ratio is zero, the system reduces into a single-objective microscope where the localization precisions are the worst; 2) When keeping the coherence strength at zero but increasing the intensity ratio, the precision can be ideally improved by a factor of $\sqrt{2}$ predicting the performance of an incoherent dual-objective microscope [9]; 3) Only when increasing both the intensity ratio and the coherence strength, the precision can be further improved. We found that the axial precision is more sensitive to the coherence strength and can be improved by a factor of 18 (maximum value divided by the minimum value, 41.1 nm/2.3 nm) comparing with single objective cases. We also noticed that, the increasing speed of the axial precision slows down when the intensity ratio and coherence strength are sufficiently large, such as within the dark blue region bounded by an axial precision of 10 nm. This indicates that 4PiPSFs, in terms of precision, have adequate tolerance with these two factors. For example, with high intensity ratio, a moderate drop in coherence strength will not drastically deteriorate the resolution, which is especially important for thick sample imaging, where the coherence strength is depth dependent.

3. Increase of information content using interferometric PSF

Here we provide a generic proof on the increase of the information content by using an interferometric PSF. Consider a simple interferometric system that has two channels, the formation of its PSF can be considered as a redistribution of the emitted photons of a single emitter into two channels. The sum of the PSF patterns from the two channels produces a conventional incoherent PSF,

$$\mu = \mu_1 + \mu_2, \tag{35}$$

where the PSF of each channel is

$$\mu_1 = g(x,y,z)\mu,$$
$$\mu_2 = [1 - g(x,y,z)]\mu, \quad [36]$$

where $g(x,y,z)$, the same size of μ, is a function of the emitter's x, y and z positions with pixel values at 0 to 1. This function can be considered as a redistribution function of the emitted photons. To quantify the information content of a PSF pattern, we use the Fisher information matrix [10]. Under the assumption that the pixel values follow a Poisson process and are independent, the Fisher information matrix of an incoherent PSF is [10]

$$F_{\mathrm{W}ij} = \sum_q \frac{1}{\mu_q}\frac{\partial \mu_q}{\partial \theta_i}\frac{\partial \mu_q}{\partial \theta_j}, \theta = (x,y,z), \quad [37]$$

where q is the pixel index. And the Fisher information matrix of a two-channel interferometric PSF is [8]

$$F_{\mathrm{I}ij} = \sum_q \frac{1}{\mu_{1q}}\frac{\partial \mu_{1q}}{\partial \theta_i}\frac{\partial \mu_{1q}}{\partial \theta_j} + \frac{1}{\mu_{2q}}\frac{\partial \mu_{2q}}{\partial \theta_i}\frac{\partial \mu_{2q}}{\partial \theta_j}. \quad [38]$$

Considering only the diagonal terms of the Fisher information matrix, which are the dominant factors when all estimation parameters in θ are independent, the Fisher information of pixel q in F_{I} is

$$F_{\mathrm{I}qii} = \frac{1}{\mu_{1q}}\left(\frac{\partial \mu_{1q}}{\partial \theta_i}\right)^2 + \frac{1}{\mu_{2q}}\left(\frac{\partial \mu_{2q}}{\partial \theta_i}\right)^2, \quad [39]$$

substitute Eq. 36 to above equation, we have,

$$F_{\mathrm{I}qii} = \frac{1}{\mu_q}\left(\frac{\partial \mu_q}{\partial \theta_i}\right)^2 + \frac{\mu_q}{g_q(1-g_q)}\left(\frac{\partial g_q}{\partial \theta_i}\right)^2, \quad [40]$$

where the first term represents the information of the incoherent PSF and the second term, always greater than zero, is the extra information from the interferometric PSF. From this expression, the larger the gradient of the redistribution function $g(x,y,z)$ along a certain dimension, the higher the increase of the information associated with that dimension.

4. PR-4Pi localization algorithm

The PR-4Pi localization algorithm consists of four major steps: calibration of cavity phase φ_0, regression, ghost reduction and removal of mislocalizations.

4.1 Calibration of cavity phase

First, the obtained blinking dataset from the camera were segmented into short time series (data batches) each contains 500 frames (10 s). For each data batch, cavity phase φ_0 was obtained as described in Supplementary Note 4.2. From these time-dependent φ_0 measurements, a continuous cavity phase function, $\varphi_0(t)$, was obtained through interpolation which was used in generating the time-dependent PR-4PiPSF model at any specific data acquisition period and therefore compensates the φ_0 drift with high accuracy.

The detailed calibration procedure is as follows: For every data batch (500 frames), 1) Sub-regions of 16 × 16 pixels around the centers of isolated emitters in each frame were cropped [11] after quadrant alignment (Supplementary Note 4.11). Each isolated emitter has four sub-quadrants; 2) Sum of the four sub-quadrants generates a conventional astigmatism PSF from which PSF widths σ_x and σ_y were estimated through a 2D Gaussian fitting [12,13]; 3) The four sub-quadrants of an isolated emitter were then used to find the interference phase φ, which includes both cavity phase φ_0 and the phase shift introduced by emitter's axial position within the specimen (Supplementary Note 4.3); 4) From a scatter plot of φ vs. σ_s obtained from a large number of isolated emitters within the data batch, where $\sigma_\mathrm{s} = \sigma_x^2 - \sigma_y^2$, a single valued φ_0 was estimated for the specific data batch (Supplementary Note 4.2 and Supplementary Fig. 1). The entire dataset may contain up to 200 data batches each contains its own estimated φ_0. This time-dependent sequence of φ_0 values were then unwrapped and interpolated using a smooth spline method through MATLAB (MathWorks, Natick, MA) function *fit* (Supplementary Fig. 1). The obtained spline function represents the time-dependent cavity phase drift during the data acquisition and was used to generate a time-dependent PSF model during the regression step.

For volumetric imaging including multiple optical sections, the calibration process also need to consider the phase changes introduced by axial positions of optical sections, denoted as φ_d. Assuming the step size between two optical sections is a constant during the data acquisition and denoted as d, then φ_d can be calculated from,

$$\varphi_d = \frac{4\pi n_\mathrm{imm} d N_\mathrm{step}}{\lambda_0}\left(1 - \frac{n_\mathrm{med}^2}{n_\mathrm{imm}^2}\right), \qquad [1]$$

where λ_0 is the emission wavelength in air, and N_step is the step number, which is indexed from 0. The estimated φ_0 for each axial plane were subtracted by the corresponding φ_d and the resulting phases again form a continuous curve as in the single-optical section imaging and were fitted with a smooth spline to reduce the estimation noise. The generated spline curve represents the cavity phase evolution throughout the acquisition period excluding the extra phase difference introduced by optical sections' axial positions.

By adding back the expected φ_d for each optical section to above obtained spline curve, each optical section then carried its own time-dependent cavity phase evolution curve (Supplementary Fig. 1). Note that the φ_0 were obtained based on a precalibrated σ_{s0} - the shape metric value σ_s when a PSF is in focus, obtained from imaging beads on the cover glass (Methods). But with index mismatch aberration, the width of the infocus PSF changes as the imaging depth increases. Therefore, the use of a constant σ_{s0} for different axial planes will result in an inaccurate estimation of φ_0. However, for the demonstrations in this work, the maximum imaging depth is ~3 µm, within which the effect of index mismatch aberration is small (Supplementary Note 6).

With calibrated cavity phase evolution ($\varphi_{0\text{calib}}$), cavity-phase induced axial-drift (z_{φ_0}) were automatically compensated every 10 s, within which an average of z_{φ_0} can be estimated from mean$(z_{\text{rate}}\Delta\varphi_{0\text{calib}}/2)$, where z_{rate} is the axial drift caused by 1 rad of cavity-phase drift and is 45 nm from simulation. We found that the averaged z_{φ_0} within 10 s was less than 5 nm for the demonstrations on biological specimens (Figs. 3,4, Supplementary Fig. 24).

4.2 Estimation of cavity phase

The cavity phase φ_0 for a data batch (Supplementary Note 4.1) was estimated as follows: due to wrapping of the estimated interference phase φ, the scatter plot φ-σ_s resembles multiple stripe patterns that repeats from 0 to 2π (Supplementary Fig. 1). These stripes were fit with multiple lines sharing a common slope. Subsequently, the linear fit along the stripe with the highest localization density was selected from the cross-correlation of a reference image and the Gaussian blurred φ-σ_s image along the σ_s dimension (Supplementary Fig. 1). Then, the selected linear fit was used to extract the cavity phase φ_0 at $\sigma_s = \sigma_{s0}$. The value of σ_{s0} was selected by minimizing the deviations of σ_s values obtained from PR-4PiPSFs and measured PSFs for each experiment. We found that an incorrect σ_{s0} will result in a constant offset in σ_s (Supplementary Fig. 1) along the axial positions.

4.3 Regression

The regression step is to obtain the estimation parameters such as 3D locations, total photons and background photons of single emitters. It operated on small batches of the acquired data sequence. The batch size, which we used is 500 frames, is determined by the duration in which the cavity phase is relatively stable. A particular φ_0 obtained from time-dependent cavity-phase calibration was used for each data batch (Supplementary Note 4.1).

First, the obtained four quadrants (from channels S1, S2, P1 and P2) in each camera frame were aligned and summed. The summation reduces the 4Pi emission patterns into conventional incoherent emission

patterns and the summation images were used for 2D Gaussian fitting or astigmatism localization based on a Gaussian PSF model [12]. Subsequently, from the summed image, subregions (each measures 16 × 16 pixels) containing isolated single-molecule emission events were cropped [11] and then were fitted with a 2D Gaussian to obtain the initial guesses of lateral positions (x and y), and the estimations of the PSF widths σ_x and σ_y [12]. For initial estimations of the total photon and the background photon counts, the subregions were cropped without the summation of the four quadrants, so that each isolated single-molecule emission event contains four sub-quadrants. The background, b_m, for each sub-quadrants, was estimated from the median value of all edge pixels in each sub-quadrant, and the total photon count, I_m, was the sum of all pixel values for each sub-quadrant after background (b_m) subtraction, where m denotes the four channels and $m \in$ (S1,S2,P1,P2).

Second, emission patterns with low interference contrast were removed by the following procedure: Modulation contrast values were calculated for each emitter. To this end, we first computed the Gaussian-weighted sum of each sub-quadrant [2], denoted as $G_m, m \in$ (S1,S2,P1,P2), which is the sum of all pixel values of each sub-quadrant after multiplication of a 2D Gaussian with a width of one pixel (Gaussian-weighted 0th moment (M0) as defined in [2], Supplementary Figs. 6-7). Then the modulation contrast was calculated from [1],

$$r_s = \frac{G_{\text{S1}} - G_{\text{S2}}}{G_{\text{S1}} + G_{\text{S2}}},$$
$$r_p = \frac{G_{\text{P1}} - G_{\text{P2}}}{G_{\text{P1}} + G_{\text{P2}}}, \qquad [2]$$
$$I_{\text{contrast}} = \sqrt{r_s^2 + r_p^2}\,.$$

And emitters with a modulation contrast lower than 0.3 were discarded, since they usually contain overlapping emitters or have low signal to noise ratios (SNR). Interference phase $\varphi = \arctan(r_p/r_s)$ was calculated for each emitter.

Third, we seek to find reasonable initial guesses of axial positions from the remaining emission patterns. To this end, a stack of 51 PR-4PiPSFs at z positions from -1 to 1 µm with a given φ_0 was generated and subsequently, initial axial position guesses were obtained through cross-correlations between each emitter's sub-quadrants with the pre-obtained 4PiPSF stack (Supplementary Figs. 1, 11).

At last, using the above obtained initial guesses on lateral and axial positions, total photon and background photon counts, a maximum likelihood estimation (MLE) was used to obtain estimates for all 11 parameters (considering independent intensity and background for each channel: x, y, z, I_m, b_m, $m =$ S1,S2,P1,P2) or 5 parameters (considering equal split of photon energy through the four detected

channels: x, y, z, I, b) (referred as θ) [14]. The likelihood function for each 4Pi emission pattern can be written as,

$$L(\theta|D) = \prod_m \prod_q \frac{(\mu_{mq} + \gamma_{mq})^{(D_{mq}+\gamma_{mq})} e^{-(\mu_{mq}+\gamma_{mq})}}{\Gamma(D_{mq} + \gamma_{mq} + 1)}, \theta \in (x, y, z, I_m, b_m), \quad [3]$$

which includes the Poisson noise and pixel-dependent sCMOS noise [14]. D represents the experimental data, q is the pixel index and γ equals to σ^2/g^2 where σ^2 is the variance of the readout noise of each pixel and g is the gain of each pixel. Note that the subregions (D) used for MLE were cropped from original data quadrants without transformation (Supplementary Note 4.11). An optimization routine based on a modified Levenberg-Marquadt method [15] was used to minimize the negative log-likelihood function, which is given by, using the Sterling approximation,

$$-\ln(L) = \sum_m \sum_q \mu_{mq} - (D_{mq} + \gamma_{mq}) \ln(\mu_{mq} + \gamma_{mq}). \quad [4]$$

Its first and second derivatives are

$$f = -\frac{\partial \ln(L)}{\partial \theta} = \sum_m \sum_q \left(1 - \frac{D_{mq} + \gamma_{mq}}{\mu_{mq} + \gamma_{mq}}\right) \frac{\partial \mu_{mq}}{\partial \theta}, \quad [5]$$

$$f' = \frac{\partial f}{\partial \theta} = \sum_m \sum_q \frac{D_{mq} + \gamma_{mq}}{(\mu_{mq} + \gamma_{mq})^2} \left(\frac{\partial \mu_{mq}}{\partial \theta}\right)^2 + \left(1 - \frac{D_{mq} + \gamma_{mq}}{\mu_{mq} + \gamma_{mq}}\right) \frac{\partial^2 \mu_{mq}}{\partial \theta^2}. \quad [6]$$

Here, we set the term with $\partial^2 \mu_{mq}/\partial \theta^2$ in Eq. 6 to zero [15]. Then the estimation parameters were updated from,

$$\theta_{n+1} = \theta_n - \frac{f}{f'(1+\beta)}, \quad [7]$$

where β is a damping factor that was used to adjust the convergence speed and percentage, and here we set β to 1. We used 40 iterations for analyzing biological sample data and 100 iterations for bead data. We found PR-4Pi usually converges at 40 iterations (Supplementary Fig. 13).

4.4 Ghost reduction

The likelihood function used for regression has many local minimums along the axial dimension, which come from the periodicity in the intensity modulation of the interferometric PSFs. Those local minimums are separated by a distance close to the modulation period, ~300 nm. With low SNR or overlapping emitters, the optimization process can be trapped at the local minimums and results in ghost images of a single emitter [2,4]. Here we developed a ghost reduction (GR) algorithm exploiting the periodicities of these local minimums to reduce the ghost images, which was applied during the regression step

(Supplementary Fig. 1). The GR algorithm consists of three steps: select a candidate image of the single emitter, calculate the ghost probabilities, update the determined ghost images.

selection of candidate image

1) Within each data batch of N frames, we calculate the lateral and axial pairwise distances d_{xy} and d_z for all localized emitters. 2) Emitters that are close in lateral dimension (d_{xy}< 50 nm) but with an axial separation (i.e. 270 < d_z < 320 nm for Alexa Fluor 647 tagged sample) near the modulation period are grouped together as ghost pairs. Each ghost pair can contain multiple clusters of points, which are mutual ghost images of each other (Supplementary Fig. 1). 3) Select one cluster from each ghost pair as the candidate image of the single emitter based on a selection criterion,

$$j_0 = \underset{j}{\operatorname{argmax}}\left[p_j = \max\left(\frac{I_{ij}}{LLR_{ij}}\right)\right], \quad [8]$$

where I is the sum of estimated photons (I_m, Main Text Eq. 5) of the four sub-quadrants, j the cluster index and i the index of each localization in the cluster. And LLR represents the log-likelihood ratio [11,16] of each localization,

$$\begin{aligned} LLR &= -2\ln\left(\frac{L(\mu|D)}{L(D|D)}\right) \quad [9] \\ &= \sum_m \sum_q 2\left[\mu_{mq} - D_{mq} + (D_{mq}+\gamma_{mq})\ln\left(D_{mq}+\gamma_{mq}\right) - (D_{mq}+\gamma_{mq})\ln\left(\mu_{mq}+\gamma_{mq}\right)\right], \end{aligned}$$

Where q is the pixel index and m is the channel index and $m \in$ (S1,S2,P1,P2). The selection method first finds the maximum ratio of I/LLR within each cluster, denoted as p, then select the cluster with the largest p as the candidate image (cluster j_0).

calculation of ghost probability

We assume the candidate image is the real image of the emitter, a naive GR algorithm is to remove all the rest images of that emitter. Apparently, this simple method will remove any periodic localizations, with a period near the modulation period (z_T), along the axial dimension and prone to change the sample structures. In fact, not all images in one ghost pair belong to a single emitter, some might be the real images of other emitters that happen to be ~z_T distance away. Therefore, we calculated the probability of each cluster being a ghost image of the candidate cluster, named as the ghost probability. The detailed calculation is as follows: 1) For the same data batch, obtain axial position estimations using the conventional astigmatism localization method [12], denoted as z_{ast}. The astigmatism method is ghost free, so that the real ghost localizations from PR-4Pi should collapse into one cluster when replacing the axial positions with z_{ast}. 2) For each ghost pair, calculate the standard deviations of the estimations in x, y and

z_{ast} of the candidate cluster, denoted as σ_{gx}, σ_{gy} and σ_{gz}, but restricted to a set range (10-30 nm for x and y and 40-100 nm for z_{ast}). 3) Calculate the center of the candidate cluster from the medians of the estimations in x, y and z_{ast}, denoted as x_0, y_0 and z_{ast0}. 4) Calculate the centers of the rest clusters in the same ghost pair, denoted as $(x_i, y_i, z_{\text{ast}i})$, then the normalized probability of cluster i belonging to the candidate cluster is

$$p = e^{-(x_i - x_0)^2/2\sigma_{gx}^2 - (y_i - y_0)^2/2\sigma_{gy}^2 - (z_{\text{ast}i} - z_{\text{ast0}})^2/2\sigma_{gz}^2}, \quad [10]$$

And the axial component of the above probability is

$$p_z = e^{-(z_{\text{ast}i} - z_{\text{ast0}})^2/2\sigma_{gz}^2}. \quad [11]$$

During regression, if either $p > 0.001$ or $p_z > 0.1$, we consider cluster i is a ghost image of the candidate cluster and set all the axial localizations in this cluster to z_0, which is the median of the axial localizations of the candidate cluster from PR-4Pi. Otherwise, we consider cluster i as the localizations of a different emitter and keep the original localization results. 5) Assign the updated axial localizations as the new initial guesses of the z positions, and relocalize the emitters with PR-4Pi.

4.5 Removal of mislocalizations

Theoretically, when noise and aberration are absent, all (σ_s, φ) pairs should lay on top of the linear fitted lines (Supplementary Fig. 1). Since φ is linearly related to the axial position (z), it is expected that all (σ_s, z) pairs should be concentrated along a line in the σ_s- z plot. Therefore, we computed the local density of each emitter in the σ_s- z plot and removed the emitters with a local density smaller than a set threshold. We found that after the removal of mislocalizations, the percentage of ghost images was further reduced to 2% and the resulting distributions of pairwise distances are comparable with the ones from astigmatism method (Supplementary Fig. 1).

4.6 Drift correction

A redundancy-based drift correction method [1] was used for 3D drift correction within a single optical section. Specifically, a 3D image stack was generated for each data batch (2000-4000 frames) with a voxel size of 32×32×32 nm^3 and each 2D image was smoothed with a 2D Gaussian with its standard deviation at each dimension equal to 64 nm. And the x, y and z shifts between every two data batches (with redundant pairs) were obtained from 3D cross-correlations of the two image stacks. A least-square optimization algorithm was used to estimate the shifts between consecutive data batches from obtained redundant shifts. Data batches were then realigned based the optimized shifts. For data with multiple optical sections, redundancy-based drift correction first operated on each optical section and then realign

the whole dataset with each optical section as one data batch. In the latter step, an initial guess of the axial shift equal to the step size between consecutive optical sections multiplied with $n_{\mathrm{med}}/n_{\mathrm{imm}}$, where n_{med} and n_{imm} are the refractive indices of the sample medium and the immersion oil respectively, was used to obtain the final axial shift from 3D cross-correlation between two optical sections.

4.7 Rejection

To improve the image resolution, localizations with low photon count, high background and high LLR were rejected. Photon threshold was calculated at the peak of the photon distribution from all localizations and localizations with a photon count below the threshold were removed. Background threshold was estimated at $\mu + 3\sigma$ from a Gaussian fitting (with mean at μ and standard deviation at σ) of the background distribution from all localizations and localizations with a background above the threshold were removed. Localizations with an LLR larger than 2000 (PR-4Pi) and 300 (contrast) were removed.

4.8 SMSN reconstruction

Except for decorrelation analysis [3] (Supplementary Fig. 26), SMSN reconstructions were generated at a pixel size of 3.2~4.3 nm, and all localizations in each image were blurred equally with a fixed 2D Gaussian of its standard deviation at each dimension equal to 4.8~8.6 nm (according to the median of CRLB-predicated localization precision, Supplementary Fig. 25). A 3-digit color index was assigned to each localization representing its axial position and colors within one pixel were summed in the reconstructed image. For decorrelation analysis, in order to reconstruct an image that reflects the actual localization precisions, SMSN reconstructions were generated at a pixel size of 1.3~2.6 nm and each localization was blurred by a normalized 2D Gaussian with its standard deviation at each dimension equal to the theoretical estimation precision (based on CRLB) at the corresponding dimension. Gray scale images of selected subregions were used for decorrelation analysis.

4.9 Astigmatism modification of 4PiPSF

To introduce asymmetry of the 4PiPSF patterns above and below the focal plane and extend the axial localization range, we modified the 4PiPSFs by adding astigmatism aberration to the upper and lower emission paths (Methods). We found that astigmatism modification has little effect on the localization precisions comparing with none-astigmatism PSFs (Supplementary Fig. 10). In fact, when we examine the information content of 4PiPSF at single pixel level, for both astigmatism and none-astigmatism cases, substantial information on lateral molecular position locates outside the central peak due to the non-smooth and concentric ring pattern when the molecule is out of focus as shown in Supplementary Fig. 8.

Importantly, 4PiPSF localization benefits from astigmatism by reducing pattern similarities above and below the focus plane. This, in turn, minimizes the percentage of localization artifacts/ghosts in the resulting 4Pi-SMSN reconstruction.

In addition, the possibility of extending axial localization range is beneficial for whole-cell imaging or reconstruction of a tissue specimen. The extended axial volume of 4Pi-SMSN could reduce wasteful photo-bleaching and allow the efficient use of emitted photons from single molecules located away from the common focal plane.

4.10 Phase retrieval

We used a previously developed phase retrieval algorithm to obtain the pupil functions of lower and upper emission paths [8,13]. In short, the measured PSF images from a 40 nm bead at axial positions from -1 to 1 μm with 400 nm step size were processed through a Gerchberg-Saxon algorithm to obtain the pupil function. The pupil function of lower emission path (through the lower objective) varies little between different experiments. However, the pupil function of the upper emission path (through the upper objective) may vary from one sample to another due to the variations of the sample chamber height, defined by the distance between top and bottom coverslips. In our experiments, we used the pupil functions retrieved from beads attached to the bottom coverslip inside the biological samples for localization of single molecules. In cases where beads cannot be found/attached in a particular cell specimen, we either retrieved the pupil functions from in vitro specimens or used the pupil functions from previous specimens with matching sample chamber height (with a tolerance of ±1 μm, typically 6-8 μm in our experiments).

4.11 Quadrant alignment

Data for quadrant alignment are images of sparse beads sample with 5-10 beads per field of view (FOV) or cell sample illuminated with a low laser power (Methods). Each of those datasets contains 40-100 frames collected from the lower emission path. A previously developed alignment procedure [1] was applied: 1) Four quadrants were cropped from the raw camera frames and each measures 168 × 168 pixels; 2) Select one quadrant as the reference, and find the linear transformations, including scaling, translation and rotation, from each quadrant to the reference quadrant using the Dipimage [17] functions *fmmatch* and *find_affine_trans* sequentially; 3) Align each quadrant to the reference quadrant using the Dipimage function *affine_trans* given the obtained transformation relations.

For PR-4Pi method, to achieve maximum likelihood estimation (MLE), the statistical behavior (mean and variance) of the pixel value should remain unchanged. Therefore, instead of transforming the raw data, we

directly incorporated the transformation relationships between quadrants inside the likelihood function during MLE. Specifically, during the segmentation procedure (Supplementary Fig. 1), we generated two sets of subregions, one was from aligned quadrants and used for cavity phase calibration and initial guess for the lateral positions, and another one was from unaligned quadrants and used for MLE. The segmentation procedure for unaligned quadrants was as follows: 1) Select candidate emitters from the sum of the aligned quadrants and record the pixel coordinates of the candidate emitters as *coords1*. 2) Transform *coords1* to quadrants 2 to 4, assume quadrant 1 is the reference, and record the coordinates as *coords2*, *coords3* and *coords4*. 3) Crop subregions in each unaligned quadrant with the nearest integer of the corresponding *coords* as the centers. The variance and gain maps for each subregion were also cropped during this step. 4) Record the local shift of each subregion as

$$s_{xy} = coords \; - \; \mathrm{round}(coords),$$

which will be applied to the likelihood function during MLE.

5. Linear and cubic interpolations in PR-4Pi algorithm

The speed of PR-4Pi localization method depends majorly on the speed of generating PR-4PiPSF models, each requiring multiple Fourier transforms. Therefore, we replaced the Fourier transform with interpolation and implemented it with MLE (Supplementary Note 4.3 and Supplementary Fig. 1) on GPU to speed up the regression process. The interpolation algorithm generates 4PiPSFs at arbitrary locations by interpolating a pre-generated PR-4PiPSF library, termed as PSFlib, which covers an axial range of -1.5 to 1.5 µm and a lateral extend of 25 × 25 camera pixels, and each voxel of the PSFlib measures 0.25 pixel × 0.25 pixel × 5 nm. Together with the generation of the 4PiPSF model, whose first and second derivatives were also calculated during the interpolation process.

We implemented both linear and cubic interpolation methods in the PR-4Pi algorithm and we found that linear interpolation tends to produce larger artifact when operating on complex PSF patterns such as 4PiPSFs (Supplementary Fig. 15). However, both methods achieve comparable resolutions on localizing either bead or SMSN data (not shown). The GPU implementations of linear and cubic interpolations achieve a localization speed of 1140 emitter/s and 260 emitters/s respectively, nearly 600 times faster than the corresponding CPU implementations using MATLAB (MathWorks, Natick, MA). In this work, cubic interpolation was used for all the demonstrations on PR-4Pi algorithm.

5.1 Linear interpolation

Consider a one-dimensional (1D) situation, a linear interpolation between sample positions x_1 and x_2 is,

$$f(x) = ax + b, x \in [x_1, x_2], \quad [17]$$

where a and b are interpolation coefficients. Given the function values, f_1 and f_2, at the sample positions, solve the following linear equations for a and b,

$$\begin{aligned} ax_1 + b &= f_1, \\ ax_2 + b &= f_2, \end{aligned} \quad [18]$$

we have

$$\begin{aligned} a &= \frac{f_2 - f_1}{\Delta x}, \\ b &= \frac{x_2 f_1 - x_1 f_2}{\Delta x}, \end{aligned} \quad [19]$$

where Δx is the distance between the sample positions and equal to $x_2 - x_1$. The first and second derivatives of the interpolation function $f(x)$ is a and 0 respectively.

To generalize above calculations to three dimensions, we used a chain of 1D interpolations to obtain the function value at any location inside a voxel defined by the sample positions (x_1, y_1, z_1) and (x_2, y_2, z_2). The 1D interpolation functions were calculated in the order of x dimension, followed by y and z dimensions,

$$\left.\begin{matrix} \left.\begin{matrix} f(x, y_1, z_1) \\ f(x, y_2, z_1) \end{matrix}\right\} \rightarrow f(x, y, z_1) \\ \left.\begin{matrix} f(x, y_1, z_2) \\ f(x, y_2, z_2) \end{matrix}\right\} \rightarrow f(x, y, z_2) \end{matrix}\right\} \rightarrow f(x, y, z), \quad [20]$$
$$x \in [x_1, x_2], y \in [y_1, y_2], z \in [z_1, z_2].$$

The interpolation functions at each dimension can be written as,

$$\begin{aligned} f(x, y_s, z_s) &= ax + b, \\ f(x, y, z_s) &= a'(x)y + b'(x), \\ f(x, y, z) &= a''(x, y)z + b''(x, y), \end{aligned} \quad [21]$$

where s denotes the sample positions. Therefore, the first derivative of $f(x, y, z)$ with respect to x, y and z can be also calculated from a chain of 1D interpolations in the same order,

$$\left.\begin{array}{l}\left.\begin{array}{l}\frac{\Delta f}{\Delta x}(x,y_1,z_1)\\ \frac{\Delta f}{\Delta x}(x,y_2,z_1)\end{array}\right\}\rightarrow\frac{\Delta f}{\Delta x}(x,y,z_1)\\ \left.\begin{array}{l}\frac{\Delta f}{\Delta x}(x,y_1,z_2)\\ \frac{\Delta f}{\Delta x}(x,y_2,z_2)\end{array}\right\}\rightarrow\frac{\Delta f}{\Delta x}(x,y,z_2)\end{array}\right\}\rightarrow\frac{\Delta f}{\Delta x}(x,y,z), \quad [22]$$

$$\left.\begin{array}{l}\frac{\Delta f}{\Delta y}(x,y,z_1)\\ \frac{\Delta f}{\Delta y}(x,y,z_2)\end{array}\right\}\rightarrow\frac{\Delta f}{\Delta y}(x,y,z), \quad [23]$$

$$\frac{\Delta f}{\Delta z}(x,y,z) = a''(x,y). \quad [24]$$

The second derivatives are zero. By evaluating a series of 1D linear interpolations, we can obtain the function value at any location inside a given voxel as well as its first and second derivatives with respect to each dimension.

5.2 Cubic interpolation

Consider a one-dimensional (1D) situation, a cubic interpolation between sample positions x_1 and x_2 is,

$$f(x) = ax^3 + bx^2 + cx + d, x \in [x_1, x_2], \quad [25]$$

where a, b, c and d are interpolation coefficients. To obtain those four coefficients, two more sample points are required, one preceding x_1 and one after x_2, which are x_0 and x_3. Given the function values at the four sample positions, f_0, f_1, f_2 and f_3, solve the following linear equations for a, b, c and d,

$$\begin{aligned} ax_1^3 + bx_1^2 + cx_1 + d &= f_1,\\ ax_2^3 + bx_2^2 + cx_2 + d &= f_2,\\ 3ax_1^2 + 2bx_1 + c &= f'(x_1),\\ 3ax_2^2 + 2bx_2 + c &= f'(x_2), \end{aligned} \quad [26]$$

we have

$$\begin{aligned} a &= \frac{f'(x_1) + f'(x_2)}{\Delta x^2} + \frac{2(f_1 - f_2)}{\Delta x^3},\\ b &= \frac{f'(x_2) - f'(x_1)}{2\Delta x} - \frac{3(x_1 + x_2)a}{2},\\ c &= f'(x_1) - 3ax_1^2 - 2bx_1,\\ d &= f_1 - ax_1^3 - bx_1^2 - cx_1, \end{aligned} \quad [27]$$

where Δx is the distance between adjacent sample points, and the first derivatives of $f(x)$ at x_1 and x_2 were calculated from the central discrete derivative,

$$f'(x_1) = \frac{f_2 - f_0}{2\Delta x}, \quad f'(x_2) = \frac{f_3 - f_1}{2\Delta x}. \tag{28}$$

Then the first and second derivatives of the interpolation function are,

$$f'(x) = ax^2 + bx + c, \quad f''(x) = ax + b. \tag{29}$$

To generalize above calculations to three dimensions, we used a chain of 1D interpolations to obtain the function value at any location inside a voxel defined by the sample positions (x_1, y_1, z_1) and (x_2, y_2, z_2). The 1D interpolation functions were calculated in the order of x dimension, followed by y and z dimensions,

$$\left.\begin{array}{l} \left.\begin{array}{l} f(x,y_1,z_1) \\ f(x,y_2,z_1) \end{array}\right\} \rightarrow f(x,y,z_1) \\ \left.\begin{array}{l} f(x,y_1,z_2) \\ f(x,y_2,z_2) \end{array}\right\} \rightarrow f(x,y,z_2) \end{array}\right\} \rightarrow f(x,y,z), \tag{30}$$
$$x \in [x_1, x_2], y \in [y_1, y_2], z \in [z_1, z_2].$$

The interpolation function at each dimension can be written as,

$$\begin{aligned} f(x, y_s, z_s) &= ax^3 + bx^2 + cx + d, \\ f(x, y, z_s) &= a'(x)y^3 + b'(x)y^2 + c'(x)y + d'(x), \\ f(x, y, z) &= a''(x,y)z^3 + b''(x,y)z^2 + c''(x,y)z + d''(x,y), \end{aligned} \tag{31}$$

where *s* denotes the sample positions. Therefore, the first derivative of $f(x,y,z)$ with respect to x, y and z can be also calculated from a chain of 1D cubic interpolations in the same order,

$$\left.\begin{array}{l} \left.\begin{array}{l} \frac{\Delta f}{\Delta x}(x,y_1,z_1) \\ \frac{\Delta f}{\Delta x}(x,y_2,z_1) \end{array}\right\} \rightarrow \frac{\Delta f}{\Delta x}(x,y,z_1) \\ \left.\begin{array}{l} \frac{\Delta f}{\Delta x}(x,y_1,z_2) \\ \frac{\Delta f}{\Delta x}(x,y_2,z_2) \end{array}\right\} \rightarrow \frac{\Delta f}{\Delta x}(x,y,z_2) \end{array}\right\} \rightarrow \frac{\Delta f}{\Delta x}(x,y,z), \tag{32}$$

$$\left.\begin{array}{l} \frac{\Delta f}{\Delta y}(x,y,z_1) \\ \frac{\Delta f}{\Delta y}(x,y,z_2) \end{array}\right\} \rightarrow \frac{\Delta f}{\Delta y}(x,y,z), \tag{33}$$

$$\frac{\Delta f}{\Delta z}(x,y,z) = a''(x,y)z^2 + b''(x,y)z + c''(x,y). \quad [34]$$

Similarly, we can obtain the second derivatives of $f(x,y,z)$. By evaluating a series of 1D cubic interpolations, we can obtain the function value at any location inside a given voxel as well as its first and second derivatives with respect to each dimension. With this method, there is no need to store a huge number of interpolation coefficients [18,19] (64 for each voxel) to generate PSFs at arbitrary locations. And the evaluation of a 1D cubic interpolation is sufficiently fast.

6. Effect of index mismatch aberration

For oil immersion objective, the refractive index of the sample medium, e.g. 1.351 for SMSN imaging, is usually smaller than the one of the immersion oil, which is 1.516 for our 4Pi-SMSN system. This index mismatch will result in aberrations for imaging emitters away from the cover glass and the distortion increases with the imaging depth. Two methods can be used to incorporate the index mismatch aberration into the PR-4PiPSF model.

The first method assumes that the imaging depth is small, less than 4 μm, and within this range, the differences of PSF patterns from the ones measured at the cover glass are small [8]. Therefore, the PSF model of emitters on the bottom cover glass was used for the entire imaging depth. To generate such a PSF model, the pupil functions, h_A and h_B, were retrieved independently through the upper and lower objectives by imaging a bead on the bottom cover glass. The upper emission pupil-function h_A includes the index mismatch aberration caused by imaging through the entire sample medium (Supplementary Fig. 14). Therefore, the PR-4PiPSF model based on this pair of coherent pupil functions can accurately represent the emission patterns of a bead on the cover glass (Supplementary Fig. 3). However, in SMSN imaging, the defocused PSFs were formed by emitters at different axial locations relative to the sample medium instead of by moving the sample stage, which changes the thickness of the immersion medium in the case of bead imaging. The modulation period of the emission patterns in the sample medium is larger than the one in the immersion medium. Therefore, a factor of $n_\mathrm{imm}/n_\mathrm{med}$ is multiplied to the estimated emission wavelength from bead imaging (Supplementary Note 1).

The second method is to build a PSF model that can model the emission patterns came from arbitrary axial locations inside the sample medium. To achieve this, we first excluded index mismatch aberration from the phase retrieved pupil functions: the upper emission pupil-function h_A was retrieved from imaging a bead on the top cover glass through the upper objective, while the lower emission pupil-function h_B was retrieved from imaging a bead on the bottom cover glass through the lower objective (Supplementary Fig. 14). Next, we derived a PSF model incorporating index mismatch aberration by

conducting a 'gedanken-experiment' as the following: first, let both objectives focus on the cover glass close to themselves and then move the upper objective a distance of H to focus on the bottom cover glass; second move the sample stage a distance of d to focus on the target image plane inside the specimen, then the upper and lower emission pupil functions describing emitters at this image plane can be modeled as,

$$h_{\mathrm{IMA}} = h_{\mathrm{A}} e^{i\varphi_{\mathrm{IM}}(H-d)},$$
$$h_{\mathrm{IMB}} = h_{\mathrm{B}} e^{i\varphi_{\mathrm{IM}} d}, \quad [12]$$

where φ_{IM} is the aberration phase caused by index mismatch [8],

$$\varphi_{\mathrm{IM}}(z_{\mathrm{stage}}) = \frac{2\pi}{\lambda}\left[z_{\mathrm{stage}} \frac{n_{\mathrm{med}}^2}{n_{\mathrm{imm}}} \cos(\theta_{\mathrm{med}}) - z_{\mathrm{stage}} n_{\mathrm{imm}} \cos(\theta_{\mathrm{imm}})\right] \quad [13]$$

and it varies with the stage position, z_{stage}, which is the distance of the stage moved to focus on a plane away from the designed focal plane of the objective. Note that the value of z_{stage} is relative to the immersion medium, in the case of 4Pi-SMSN system, there are two stage positions, d and $H - d$, where H is a constant during imaging and those two quantities were only varied by the distance d the sample stage moved. Therefore, at a particular d the index mismatch aberrations φ_{IM} are fixed, the emission patterns are only dependent on the emitters' axial locations z inside the sample medium,

$$z = d\frac{n_{\mathrm{med}}}{n_{\mathrm{imm}}} + z', \quad [14]$$

which is zero at the bottom cover glass, and z' is a relative position [8] to the image plane position $dn_{\mathrm{med}}/n_{\mathrm{imm}}$. Together by replacing h_{A} and h_{B} with h_{IMA} and h_{IMB}, and change the defocus term to

$$D(z) = e^{i\frac{2\pi}{\lambda} n_{\mathrm{med}} \cos(\theta_{\mathrm{med}}) z'}, \quad [15]$$

in Main Text Eq. 1, the 4PiPSF model at arbitrary axial positions can be calculated. The wavelength λ used here is the one measured from bead imaging, which is approximate to the emission wavelength in air (Supplementary Note 1).

We found that the PSF models generated from both methods are consistent in a large axial range (Supplementary Fig. 14). In this work, we used the first method for PSF modeling because all the presented data were acquired within a small imaging depth. For thick sample imaging, the second method could be used to account for the depth-dependent index mismatch aberration.

However, the second method requires an accurate measurement of H, apparent distance between the top and bottom coverslips, which varies between different experiments using current sample mounting technique. To simplify the data acquisition procedure, we used the first method for PSF modeling because all the presented data were acquired within a small imaging depth. For thick sample imaging, the second

method could be used to account for the depth-dependent index mismatch aberration, and sample mounting techniques with fixed chamber height could be explored to simplify its implementation.

7. Temperature modulation on 4PiPSFs

To quantify the effect of temperature fluctuation on 4Pi-SMSN system, we measured the interference phase φ from bead imaging (Supplementary Note 4.3) and simultaneously collected the current temperature using a temperature sensor (TSP01, Thorlabs) at a sequence of 500 time points with 10 s intervals. From the measured interference phase φ and together with the axial position z_{ast} of the emitter, localized with astigmatism method, the effective axial drift caused by the change of cavity phase φ_0 can be calculated from,

$$z_{\varphi_0} = \varphi z_T/2\pi - z_{\mathrm{ast}}, \qquad [16]$$

where z_T is the modulation period of the 4PiPSF with respect to the immersion medium and the cavity phase φ_0 can be estimated from $2\pi\, z_{\varphi_0}/z_T$. We found that the fluctuation of z_{φ_0} closely follows the temperature fluctuation, which indicates that the cavity temperature has noticeable effect on the emission pattern of 4Pi-SMSN systems (Supplementary Fig. 19).

References

1. Huang, F. *et al.* Ultra-High Resolution 3D Imaging of Whole Cells. *Cell* **166,** 1028–1040 (2016).

2. Aquino, D. *et al.* Two-color nanoscopy of three-dimensional volumes by 4Pi detection of stochastically switched fluorophores. *Nat. Methods* **8,** 353–359 (2011).

3. Descloux, A., Grußmayer, K. S. & Radenovic, A. Parameter-free image resolution estimation based on decorrelation analysis. *Nat. Methods* **16,** 918–924 (2019).

4. Shtengel, G. *et al.* Interferometric fluorescent super-resolution microscopy resolves 3D cellular ultrastructure. *Proc. Natl. Acad. Sci. U. S. A.* **106,** 3125–3130 (2009).

5. Brown, T. A. *et al.* Superresolution Fluorescence Imaging of Mitochondrial Nucleoids Reveals Their Spatial Range, Limits, and Membrane Interaction. *Mol. Cell. Biol.* **31,** 4994–5010 (2011).

6. Aquino Maier, D. Single marker switching nanoscopy with 4Pi detection: Superior nanometric 3D resolution. *Diss. Ruprecht-Karls-Universität Heidelb.* (2011). doi:10.1360/zd-2013-43-6-1064

7. Hanser, B. M., Gustafsson, M. G. L., Agard, D. A. & Sedat, J. W. Phase-retrieved pupil functions in wide-field fluorescence microscopy. *J. Microsc.* **216,** 32–48 (2004).

8. Liu, S., Kromann, E. B., Krueger, W. D., Bewersdorf, J. & Lidke, K. A. Three dimensional single molecule localization using a phase retrieved pupil function. *Opt. Express* **21,** 29462 (2013).

9. Xu, K., Babcock, H. P. & Zhuang, X. Dual-objective STORM reveals three-dimensional filament organization in the actin cytoskeleton. *Nat. Methods* **9,** 185–188 (2012).

10. Ober, R. J., Ram, S. & Ward, E. S. Localization Accuracy in Single-Molecule Microscopy. *Biophys. J.* **86,** 1185–1200 (2004).

11. Huang, F., Schwartz, S. L., Byars, J. M. & Lidke, K. A. Simultaneous multiple-emitter fitting for single molecule super-resolution imaging. *Biomed. Opt. Express* **2,** 1377 (2011).

12. Smith, C. S., Joseph, N., Rieger, B. & Lidke, K. A. Fast, single-molecule localization that achieves theoretically minimum uncertainty. *Nat. Methods* **7,** 373–375 (2010).

13. Zhang, P. *et al.* Analyzing complex single-molecule emission patterns with deep learning. *Nat. Methods* **15,** 913–916 (2018).

14. Huang, F. *et al.* Video-rate nanoscopy using sCMOS camera-specific single-molecule localization algorithms. *Nat. Methods* **10,** 653–8 (2013).

15. Laurence, T. A. & Chromy, B. A. Efficient maximum likelihood estimator fitting of histograms. *Nat. Methods* **7,** 338–339 (2010).

16. Wilks, S. S. The Large-Sample Distribution of the Likelihood Ratio for Testing Composite Hypotheses. *Ann. Math. Stat.* **9,** 60–62 (1938).

17. van Vliet, L., Luengo, C., Rieger, B., van Ginkel, M. & van Kempen, G. DIPimage: A Scientific Image Processing Toolbox for MATLAB. *Imaging* (2011). Available at: http://www.diplib.org/.

18. Babcock, H. P. & Zhuang, X. Analyzing Single Molecule Localization Microscopy Data Using Cubic Splines. *Sci. Rep.* **7,** 552 (2017).

19. Li, Y. *et al.* Real-time 3D single-molecule localization using experimental point spread functions. *Nat. Methods* **15,** 367–369 (2018).
