# Supplementary material for: Enhanced 4Pi single-molecule localization microscopy with coherent pupil based localization
Source: Commun Biol. 2020 May 8;3:220. doi: 10.1038/s42003-020-0908-2 (PMC7210115; doi:10.1038/s42003-020-0908-2)
Supplement: Supplementary file 2 — Supplementary Software [file 42003_2020_908_MOESM2_ESM.zip › PR-4Pi software/PSF Toolbox/user manual for PSF toolbox.pdf]

User Manual for PSF toolbox

This software is distributed as an accompanying software for the manuscript Sheng Liu, *et al., "Enhanced 4Pi single-molecule localization microscopy with coherent pupil based localization"*

The demo package consists of functions and scripts written in MATLAB (MathWorks, Natick, MA). The code has been tested in MATLAB version R2016a.

Required package:

DIP image toolbox (http://www.diplib.org/).

Content of PSF toolbox:

Matlab classes:

OptimPR_Ast	--	for phase retrieval
PRPSF	--	for phase retrieval
PSF_4pi	--	simulate 4PiPSFs
CalCRLB_4pi	--	calculation of CRLB for 4PiPSF model (11 parameters)
CalCRLB_4pi_consI	--	calculation of CRLB for 4PiPSF model (5 parameters)
Zernike_Polynomials	--	generation of Zernike polynomials
OTFrescale	--	OTF rescale the simulated PSFs

Example codes:

PR_example.m
PSF_4pi_example.m

Test data:

bead_bot_000_020.mat

How to run

1. Change current folder in Matlab to *PSF toolbox*.
2. Run each example code in *PSF toolbox/examples/.*

   Note: for PR_example.m, it requires user to select the center of the PSF in the pop up window.
3. Type *'help classname'* in Matlab command window for detailed help on each Matlab class.
