# Supplementary material for: Enhanced 4Pi single-molecule localization microscopy with coherent pupil based localization
Source: Commun Biol. 2020 May 8;3:220. doi: 10.1038/s42003-020-0908-2 (PMC7210115; doi:10.1038/s42003-020-0908-2)
Supplement: Supplementary file 2 — Supplementary Software [file 42003_2020_908_MOESM2_ESM.zip › PR-4Pi software/user manual for PR-4Pi software.pdf]

User Manual for PR-4Pi software

This software is distributed as an accompanying software for the manuscript Sheng Liu, *et al., "Enhanced 4Pi single-molecule localization microscopy with coherent pupil based localization"*

The demo package consists of functions and scripts written in MATLAB (MathWorks, Natick, MA). The code has been tested in MATLAB version R2016b. The current version is only supported on Windows platform with GPU equipped.

Required package:

DIP image toolbox (http://www.diplib.org/download).
NVIDIA GPU Computing Toolkit v7.5 (https://developer.nvidia.com/cuda-75-downloads-archive)
Parallel Computing Toolbox in MATLAB

Content of the software package:

SR4pi_demo	-	Matlab class for analyzing 4Pi-SMSN data
PSF Toolbox	-	A set of Matlab classes for phase retrieval (see manual for PSF Toolbox)
mex	-	mex-functions used in the software package
source code	-	c and cuda source code for major mex-functions
test data	-	test data for the demo script of the software
SR4pi_demo_example.m	-	demo script of the software

How to run

1. Change current folder in Matlab to *PR-4Pi software*.
2. Run demo script *SR4pi_demo_example.m*
   Note: there are a few steps requiring user interactions, please see the demo script for details.
3. Type *'help SR4pi_demo'* in Matlab command window for detailed help on *SR4pi_demo* class.
