# Supplementary material for: Enhanced 4Pi single-molecule localization microscopy with coherent pupil based localization
Source: Commun Biol. 2020 May 8;3:220. doi: 10.1038/s42003-020-0908-2 (PMC7210115; doi:10.1038/s42003-020-0908-2)
Supplement: Supplementary file 4 — Description of Additional Supplementary Files [file 42003_2020_908_MOESM4_ESM.pdf]

Description of Additional Supplementary Files

Supplementary Data 1: Data for generating Fig. 2b-e. Data are formatted in Excel (.xlsx).
206. S. Martin Jischke Dr. West Lafayette, IN, 47906 fanghuang@purdue.edu
liu1840@purdue.edu

Supplementary Data 2: Data for generating the profile plots in Fig. 3d,e. Data are formatted in Matlab (.mat) and the Matlab script (Fig3de_script.m) for generating the profile plots is included. Data can be accessed through Figshare.

Supplementary Data 3: Data for generating the profile plots in Fig. 4e,f. Data are formatted in Matlab (.mat) and the Matlab script (Fig4ef_script.m) for generating the profile plots is included. Data can be accessed through Figshare.

Supplementary Software: PR-4Pi software package for analyzing single molecule blinking dataset from 4Pi-SMSN systems.
