# Supplementary material for: Enhanced 4Pi single-molecule localization microscopy with coherent pupil based localization
Source: Commun Biol. 2020 May 8;3:220. doi: 10.1038/s42003-020-0908-2 (PMC7210115; doi:10.1038/s42003-020-0908-2)
Supplement: Supplementary file 5 — Reporting Summary [file 42003_2020_908_MOESM5_ESM.pdf]

nature research

Corresponding author(s): Sheng Liu and Fang Huang

Last updated by author(s): Mar 10, 2020

Reporting Summary

Nature Research wishes to improve the reproducibility of the work that we publish. This form provides structure for consistency and transparency in reporting. For further information on Nature Research policies, see Authors & Referees and the Editorial Policy Checklist.

Statistics

For all statistical analyses, confirm that the following items are present in the figure legend, table legend, main text, or Methods section.

n/a	Confirmed	
☐	☒	The exact sample size (*n*) for each experimental group/condition, given as a discrete number and unit of measurement
☐	☒	A statement on whether measurements were taken from distinct samples or whether the same sample was measured repeatedly
☒	☐	The statistical test(s) used AND whether they are one- or two-sided<br>*Only common tests should be described solely by name; describe more complex techniques in the Methods section.*
☒	☐	A description of all covariates tested
☐	☒	A description of any assumptions or corrections, such as tests of normality and adjustment for multiple comparisons
☐	☒	A full description of the statistical parameters including central tendency (e.g. means) or other basic estimates (e.g. regression coefficient) AND variation (e.g. standard deviation) or associated estimates of uncertainty (e.g. confidence intervals)
☒	☐	For null hypothesis testing, the test statistic (e.g. *F*, *t*, *r*) with confidence intervals, effect sizes, degrees of freedom and *P* value noted<br>*Give P values as exact values whenever suitable.*
☒	☐	For Bayesian analysis, information on the choice of priors and Markov chain Monte Carlo settings
☒	☐	For hierarchical and complex designs, identification of the appropriate level for tests and full reporting of outcomes
☒	☐	Estimates of effect sizes (e.g. Cohen's *d*, Pearson's *r*), indicating how they were calculated

Our web collection on statistics for biologists contains articles on many of the points above.

Software and code

Policy information about availability of computer code

Data collection	Data were collected using a custom-built 4PiSMSN instrument described in the manuscript, Methods and Supplementary Note sections. The instrument is controlled by a custom-designed LabView program.
Data analysis	Acquired single molecule datasets are analyzed using developed software 'PR-4Pi' described in Methods and Supplementary Note sections and the source codes are available as supplementary software package.

For manuscripts utilizing custom algorithms or software that are central to the research but not yet described in published literature, software must be made available to editors/reviewers. We strongly encourage code deposition in a community repository (e.g. GitHub). See the Nature Research guidelines for submitting code & software for further information.

Data

Policy information about availability of data

All manuscripts must include a data availability statement. This statement should provide the following information, where applicable:

- Accession codes, unique identifiers, or web links for publicly available datasets
- A list of figures that have associated raw data
- A description of any restrictions on data availability

The analyzing algorithms for 4Pi-SMSN data are implemented as a MATLAB class, named SR4pi_demo, which provides localization methods using PR-4Pi algorithm and contrast algorithm. An example script on using SR4pi_demo class is available in Supplementary Software and further updates will be made available at https://github.com/HuanglabPurdue/PR-4Pi.

Data underlying the plots in Fig. 2b-e, Fig. 3d,e and Fig. 4e,f are available as Excel files and via Figshare in Supplementary Data. Example test data for using PR-4Pi algorithm are also available in the Supplementary Software package. Other data that support the findings of this study are available from the corresponding author upon request.

Field-specific reporting

Please select the one below that is the best fit for your research. If you are not sure, read the appropriate sections before making your selection.

☒ Life sciences ☐ Behavioural & social sciences ☐ Ecological, evolutionary & environmental sciences

For a reference copy of the document with all sections, see nature.com/documents/nr-reporting-summary-flat.pdf

Life sciences study design

All studies must disclose on these points even when the disclosure is negative.

Sample size	For biological data, the number of sub-regions analyzed is determined by the number of emission events obtained from single molecule switching nanoscopy experiments. The sample sizes used to derive median, mean and standard deviation are reported as n = xx in the figure legends and the Main Text.
Data exclusions	No data were excluded from this study
Replication	All attempts at replication were successful
Randomization	Single molecule localizations are obtained from random subset of single emitters in fixed specimens.
Blinding	Blinding is not relevant to this study. Single molecule blinking events are stochastic emissions from single emitters within the the cell specimen.

Reporting for specific materials, systems and methods

We require information from authors about some types of materials, experimental systems and methods used in many studies. Here, indicate whether each material, system or method listed is relevant to your study. If you are not sure if a list item applies to your research, read the appropriate section before selecting a response.

Materials & experimental systems

n/a	Involved in the study	
☐	☒	Antibodies
☐	☒	Eukaryotic cell lines
☒	☐	Palaeontology
☒	☐	Animals and other organisms
☒	☐	Human research participants
☒	☐	Clinical data

Methods

n/a	Involved in the study	
☒	☐	ChIP-seq
☒	☐	Flow cytometry
☒	☐	MRI-based neuroimaging

Antibodies

Antibodies used	anti-TOM20 primary antibody;anti-Nup98 primary antibody; Alexa Fluor 647 conjugated secondary antibodies; Alexa Fluor 647 conjugated nanobodies;
Validation	anti-TOM20 primary antibody is commercially available from Santa Cruz Biotechnology (sc-11415) and has been reported previously (PMID: 27397506); anti-Nup98 primary antibody is commercially available from Cell Signaling Technology (2598) and has been reported previously (PMID: 27018580); Alexa Fluor 647 conjugated secondary antibodies are commercially available from Molecular Probes (A21245 and A21236) and has been reported previously (PMID: 27397506).

Eukaryotic cell lines

Policy information about cell lines

Cell line source(s)	COS-7 cells (CRL-1651 from ATCC)
Authentication	COS-7 cells were not authenticated
Mycoplasma contamination	Cell lines were not tested for mycoplasma contamination.
Commonly misidentified lines (See ICLAC register)	No commonly misidentified cell lines were used.
