# Supplementary material for: Enhanced 4Pi single-molecule localization microscopy with coherent pupil based localization
Source: Commun Biol. 2020 May 8;3:220. doi: 10.1038/s42003-020-0908-2 (PMC7210115; doi:10.1038/s42003-020-0908-2)
Supplement: Supplementary file 6 — Peer Review File [file 42003_2020_908_MOESM6_ESM.pdf]

Editorial Note: *This manuscript has been previously reviewed at another Nature Research journal. The manuscript was considered suitable for publication without further review at Communications Biology*
